# Impact of Reaction Conditions on Product Distribution in Oxidative Dehydrogenation of Ethane over MoVNbTeO_*x*_ Catalysts

**DOI:** 10.1021/acs.iecr.6c02345

**Published:** 2026-10-01

**Authors:** Yannick Weis, Martin Schubert, Andreas Meiswinkel, Sebastian Böcklein, Klaus Wanninger, Gerhard Mestl, Sebastian Rehfeldt, Harald Klein

**Affiliations:** † TUM School of Engineering and Design, Department of Energy and Process Engineering, Institute of Plant and Process Technology, 9184 Technical University of Munich, Garching 85748, Germany; ‡ 68213 Linde GmbH, Linde Engineering, Pullach 82049, Germany; § Clariant Produkte (Deutschland) GmbH, Heufeld 83052, Germany

## Abstract

Oxidative dehydrogenation of ethane over MoVNbTeO_*x*_ catalysts offers a less carbon-intensive alternative to steam cracking. This work systematically examines the effects of catalyst bed inert dilution, reactor inlet pressure, molar steam-to-ethane feed ratio, coolant temperature, and weight hourly space velocity on ethane conversion and product distribution under both kinetically oriented, pseudo-isothermal conditions and process-relevant, non-isothermal conditions. Comparative tests confirm that trends observed under pseudo-isothermal conditions persist under process-relevant operating conditions, validating their industrial significance. Increasing the reactor inlet pressure promotes acetic acid formation, whereas a higher weight hourly space velocity favors ethylene selectivity by limiting secondary oxidation. An optimal steam-to-ethane ratio prevents catalyst deactivation while maintaining a high catalytic activity. The ethylene yield reaches a maximum at intermediate steam-to-ethane ratios, whereas the acetic acid yield increases continuously throughout the investigated range. Apparent activation energies determined from quasi-isothermal experiments further indicate a stronger temperature dependence of ethylene formation than that of acetic acid formation. Notably, the steam partial pressure in the wet product gas is the key parameter determining the molar product ratio of ethylene to that of acetic acid. It modulates the degree of coverage of the catalyst surface with water and thus shifts the balance between active sites for ethylene or acetic acid production, enabling a robust basis for the process control strategies. Additional experiments comparing ethane and ethylene oxidation highlight the role of ethylene in the formation of trace products methane and acetylene, detected at ethane conversions above 20%. These findings provide a mechanistic basis for tuning catalytic activity and product distribution through targeted adjustment of key process variables under industrially relevant conditions.

## Graphical abstract

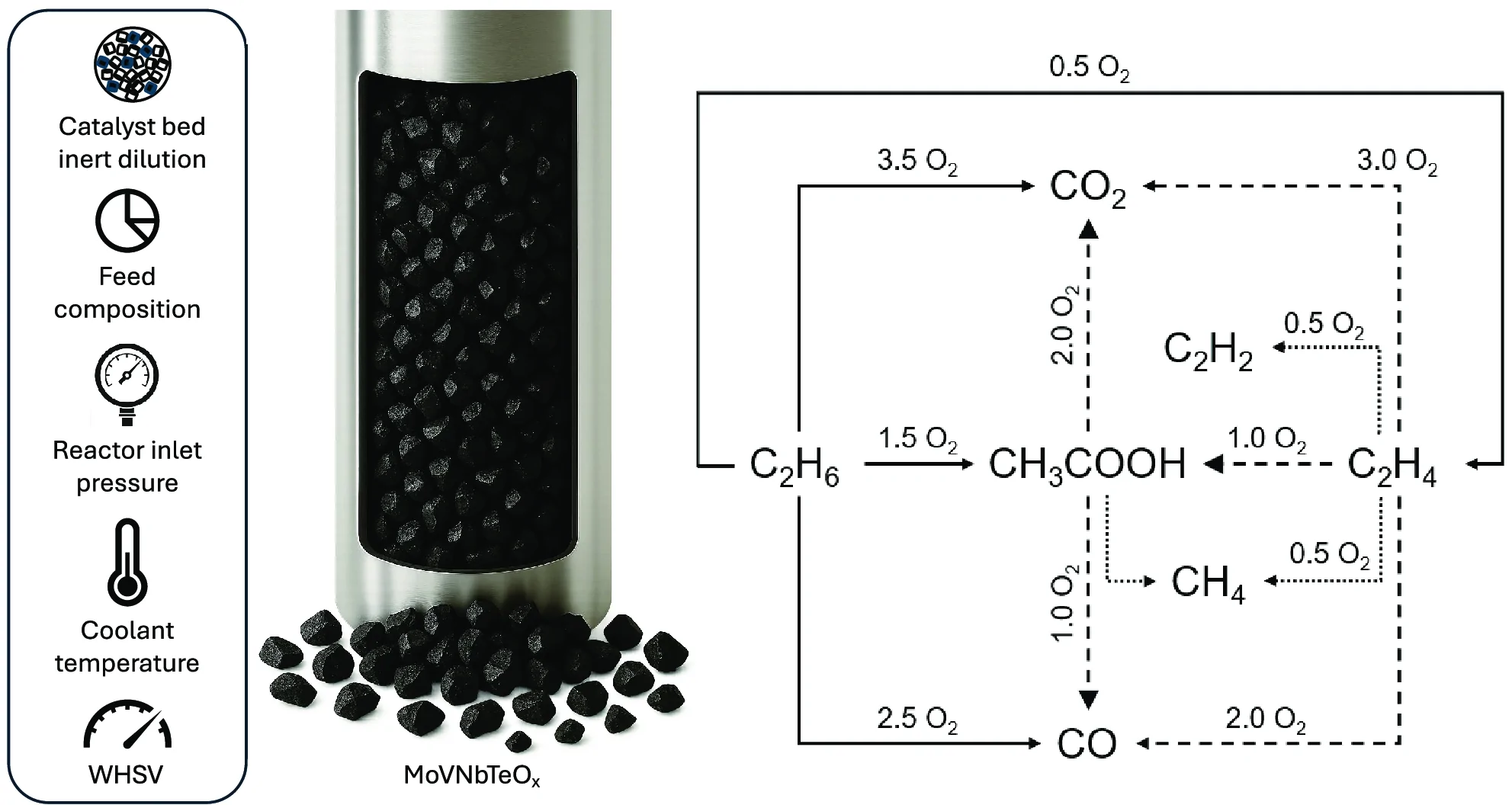

## 1 Introduction

The chemical industry is an energy-intensive sector with substantial greenhouse gas emissions.^1^ Ethylene (C_2_H_4_), with an annual production exceeding 228 million metric tons in 2023,^2^ is the most important building block in the petrochemical industry. It serves as a precursor for polymers such as polyethylene, and for chemicals including ethanol, ethylene oxide, and acetaldehyde.^3–6^ Currently, ethylene is predominantly produced via steam cracking of ethane, naphtha, or other fossil feedstock, a well-established process operating at temperatures above 800 °C.^7^

Despite decades of optimization, steam cracking remains constrained by two fundamental challenges: (i) substantial process-related greenhouse gas emissions and (ii) a wide product distribution with thermodynamically fixed ratios, limiting flexibility in ethylene production capacity.^8^ Consequently, alternative processes for ethylene production should offer both the possibility of more flexible production volumes and significant CO_2_ emission savings potential to become an attractive competitive or supporting side-by-side process to steam cracking.^9^

Oxidative dehydrogenation of ethane (ODHE), a promising alternative to steam cracking, operates at lower temperatures (300–400 °C). A wide range of heterogeneous catalyst systems has been explored for ODHE, each offering distinct advantages and limitations regarding activity, selectivity, operating temperature, and practical applicability. Reported systems span from rare-earth metal oxides^10–12^ and alkali-based catalysts^13^ to zeolites,^14^ carbon materials,^15^ and various transition-metal oxides,^16–24^ all exhibiting different mechanistic pathways and performance profiles.

Among these options, mixed metal oxides, particularly MoVNbTeO_*x*_ catalysts,^25–30^ stand out as the most promising system due to their combination of high ethylene selectivity, high activity at comparatively low operating temperature, and overall suitability for scalable operation.

ODHE over MoVNbTeO_*x*_ catalysts produces acetic acid (AcOH) alongside the main product ethylene as a valuable coproduct.^27–31^ Throughout this work, ethylene and acetic acid are referred to as commercial products (CP). Previous studies^32–34^ in scientific literature often neglected acetic acid, although it represents the second major product of ODHE.

In ODHE, the trace components methane (CH_4_), acetylene (C_2_H_2_), and hydrogen (H_2_) may also form under specific conditions and are considered in this work. While methane and acetylene are known byproducts of oxidative pyrolysis of ethane, their formation is primarily linked to homogeneous gas-phase reactions under harsh conditions at temperatures above 700 °C, with their selectivity increasing with temperature and ethane conversion.^35^ To the best of our knowledge, methane and acetylene have not been reported as trace products under process-relevant ODHE conditions in scientific literature, but are known in industry according to patent applications.^36–38^

The endothermic dehydrogenation of ethane under steam cracking conditions is shown in Reaction 1 in Table 1. The main selective and total oxidation reactions are summarized in Reactions R2–R5. Secondary oxidation pathways increase network complexity, as ethylene and acetic acid can undergo further oxidation, shown in Reactions R6–R10. Methane and acetylene can be formed via Reactions R11–R13 as trace products.

**Table 1:** Summary of Primary, Secondary, and Trace Reactions Relevant in ODHE Yielding Ethylene and Acetic Acid, Carbon Oxides as Side Products, Methane, and Acetylene as Trace Products

reaction		Δ*H*_*R*_^0^ ^a^in kJ mol^–1^
$C_{2}H_{6}\to C_{2}H_{4}+H_{2}$	(1)	+136
$C_{2}H_{6}+0.5O_{2}\to C_{2}H_{4}+H_{2}O$	(2)	–105
$C_{2}H_{6}+1.5O_{2}\to {\mathrm{CH}}_{3}\mathrm{COOH}+H_{2}O$	(3)	–590
$C_{2}H_{6}+2.5O_{2}\to 2\mathrm{CO}+3H_{2}O$	(4)	–862
$C_{2}H_{6}+3.5O_{2}\to 2{\mathrm{CO}}_{2}+3H_{2}O$	(5)	–1428
$C_{2}H_{4}+O_{2}\to {\mathrm{CH}}_{3}\mathrm{COOH}$	(6)	–485
$C_{2}H_{4}+2O_{2}\to 2\mathrm{CO}+2H_{2}O$	(7)	–757
$C_{2}H_{4}+3O_{2}\to 2{\mathrm{CO}}_{2}+2H_{2}O$	(8)	–1323
${\mathrm{CH}}_{3}\mathrm{COOH}+O_{2}\to 2\mathrm{CO}+2H_{2}O$	(9)	–272
${\mathrm{CH}}_{3}\mathrm{COOH}+2O_{2}\to 2{\mathrm{CO}}_{2}+2H_{2}O$	(10)	–838
$C_{2}H_{4}+0.5O_{2}\to {\mathrm{CH}}_{4}+\mathrm{CO}$	(11)	–238
${\mathrm{CH}}_{3}\mathrm{COOH}\to {\mathrm{CH}}_{4}+{\mathrm{CO}}_{2}$	(12)	–36
$C_{2}H_{4}+0.5O_{2}\to C_{2}H_{2}+H_{2}O$	(13)	–67

a Standard enthalpies of formation correspond to gaseous species; water and acetic acid were assumed to be in the gas phase for the reaction enthalpy calculations.

A state-of-the-art reaction network including primary and secondary pathways is available in the literature,^30,39^ however, trace reactions are not included. The large variety of possible reaction paths highlights the need for highly selective catalysts to achieve low-emission and competitive ODHE performance. Heterogeneous MoVNbTeO_*x*_ catalysts, enriched in M1 crystal phase, exhibit high activity and selectivity and allow adjustment of the molar product ratio *r*_CP_ of commercial products ethylene to acetic acid while minimizing CO_2_ formation.^32,40,41^ However, ODHE process operation is constrained by several factors: sufficient ratios of steam to ethane (*r*_H_2_O/C_2_H_6__) and oxygen to ethane (*r*_O_2_/C_2_H_6__) in the reactor feed to maintain catalyst stability, avoidance of excessive hot spot temperatures, and process limitations related to reactor pressure and weight hourly space velocity (WHSV).^42–44^

Recent efforts toward ODHE process development are pursued by various stakeholders, such as Nova Chemicals^45,46^ and Linde Engineering. At Linde Engineering, the ODHE technology is commercialized under the trademark EDHOX,^31,47^ employing a highly selective catalyst supplied by Clariant AG under the trademark OxyMax E.^48,49^

The objectives of this study are to provide a comprehensive analysis of important process parameters in ODHE under both kinetically oriented, pseudo-isothermal conditions (focused on intrinsic kinetic behavior) and process-relevant, non-isothermal conditions reaching technically relevant ethane conversions. While pseudo-isothermal conditions allow precise evaluation of intrinsic catalytic properties without thermal gradients, industrially relevant process conditions are inherently non-isothermal due to heat generation and transfer limitations. Therefore, investigating non-isothermal conditions is crucial to understanding catalyst performance under process-relevant conditions and to bridge the gap between laboratory studies and industrial applications.

The relative importance of intrinsic kinetics and transport limitations changes with reactor scale due to their different scaling behaviors. While laboratory-scale studies under pseudo-isothermal conditions primarily reflect intrinsic catalytic properties, transport effects such as heat and mass transfer increasingly influence reactor performance at larger scales. Multiscale modeling approaches are well-suited to capture this transition by linking phenomena across different length and time scales and by integrating experimental information obtained at different reactor scales into a unified reactor description.

In this context, the present experimental work focuses on process-relevant conditions to account for these scale-dependent effects and provide insights from an intermediate scale that is applicable to industrial reactor operation. In addition, operation under non-isothermal conditions at this scale enables efficient identification of relevant operating windows and sensitivities before conducting more resource-intensive pilot-scale campaigns.

Such experiments can therefore serve both to improve process understanding and to provide representative data sets for subsequent reactor modeling and scale-up studies by integrating experimental information obtained at different reactor scales into data-driven, physics-informed reactor models.

This work does not aim to derive intrinsic kinetics but investigates how key operating parameters affect ODHE performance under process-relevant conditions. Specifically, this study (i) evaluates similarities and differences between kinetically oriented, pseudo-isothermal conditions and process-relevant, non-isothermal conditions through catalyst bed inert dilution, (ii) systematically varies important process parameters to assess their impact on ethane conversion and product distribution, (iii) compares ethane and ethylene oxidation to clarify secondary reaction pathways, and (iv) analyzes trace products such as hydrogen, methane, and acetylene, which are formed under process-relevant conditions.

## 2 Experimental Section

The influence of key process parameters on the catalytic performance of MoVNbTeO_*x*_ catalysts in ODHE is investigated in a laboratory-scale reactor. Catalyst bed inert dilution, steam-to-ethane ratio *r*_H_2_O/C_2_H_6__, reactor inlet pressure *p*_in_, coolant temperature *T*_CM_, and WHSV were systematically varied. The following sections outline the catalyst preparation, experimental setup, reaction conditions, and data evaluation.

### 2.1 Catalyst Preparation

The investigated commercial MoVNbTeO_*x*_ catalyst tablets (OxyMax E) were provided by Clariant AG. The active catalyst materials were synthesized as described in respective patent applications.^48,50^ Prior to forming, MoVNbTeO_*x*_ was mixed with varied specific binder types and contents (based on aluminates and silicates) and small amounts of graphite, yielding three catalyst types (Catalyst A, B, C). Hollow cylinders were formed on a rotary tableting machine, followed by heat treatment of the tablets at a temperature >200 °C. For application in the laboratory-scale reactor, the catalyst tablets were crushed and sieved to a defined particle size fraction of 1.0–2.5 mm.

### 2.2 Experimental Setup

A simplified block flow diagram and a detailed flowsheet and description of the laboratory-scale ODHE setup, and information on equipment manufacturers and other experimental details, are reported in Section S1.1 of the Supporting Information (SI).

Ethane, oxygen, and nitrogen were supplied via mass flow controllers, while demineralized water was dosed using an HPLC pump. The water feed mass flow was monitored gravimetrically by placing the water container on a scale during operation. Nitrogen was only used as a dilutent for plant start-up and shut-down. The feed mixture was vaporized in a preheater before entering the stainless steel tubular reactor (SS321, inner tube: *D*_R_ = 10 mm, 2 mm wall thickness; outer tube: 25 mm diameter). The WHSV of the feed is calculated as the ratio of total feed mass flow rate *Ṁ*_feed_ and catalyst mass *M*_cat_ according to

1 $$\mathrm{WHSV}=\frac{{Ṁ}_{\mathrm{feed}}}{M_{\mathrm{cat}}}$$

On the ethane feed line, a continuous measurement of the reactor inlet pressure *p*_in_ is provided. Thermal oil circulated cocurrently in the outer tube for heating and heat removal and was temperature controlled by a thermal oil heater. The wet product gas (WPG) was cooled in a heat exchanger, and the two-phase product stream was separated into a liquid fraction (condensate, Cond) and a gaseous fraction (dry product gas, DPG). Condensate containers were weighed to determine the condensate mass flow rate *Ṁ*_cond_. The mass flow rate *Ṁ*_DPG_ of the dry product gas was quantified by a Coriolis flow meter, and its composition was analyzed online via gas chromatography.

A continuous measurement of the reactor outlet pressure *p*_out_ is provided on the product gas line. All pressure values reported in this work refer to absolute pressure. A temperature profile over the inert and catalyst bed is measured by multiple temperature sensors, with six uniformly distributed sensors in the catalyst bed, to indicate the maximum temperature increase Δ*T*_max_, which is calculated as the difference of hotspot temperature *T*_HS_ and coolant temperature *T*_CM_ according to

2 $$\Delta T_{\max}=T_{\mathrm{HS}}-T_{\mathrm{CM}}$$

The average catalyst bed temperature *T*_bed_ is calculated as the arithmetic mean of the temperatures measured by temperature sensors positioned within the catalyst bed. The temperature deviation Δ*T*_bed_ around the average catalyst bed temperature over the catalyst bed is determined via the difference in the highest temperature *T*_HS_ and the lowest temperature *T*_bed,min_ measured by the temperature sensors in the catalyst split bed according to

3 $$\Delta T_{\mathrm{bed}}=\frac{T_{\mathrm{HS}}-T_{\mathrm{bed},\min}}{2}$$

For each experiment, the laboratory plant was operated at steady-state conditions for a continuous two-hour period with all process variables continuously logged at a sampling rate of 0.5 s^–1^, and the representative steady-state values were obtained as the arithmetic mean over the two-hour period. The dry product gas composition was monitored by gas chromatography at regular intervals of five minutes.

### 2.3 Catalyst Bed Configuration

The catalyst bed in the tubular reactor consisted of a 54 cm layer of catalyst split (particle size fraction of 1.0–2.5 mm), enclosed by two inert layers of glass beads (2.0 mm diameter). Six temperature sensors are distributed uniformly over the catalyst bed and are introduced centrally into the reactor as a bundled assembly with different lengths from the bottom. The relevant instrumentation and the catalyst filling method of the tubular reactor are shown in Figure 1.

**Figure 1:**
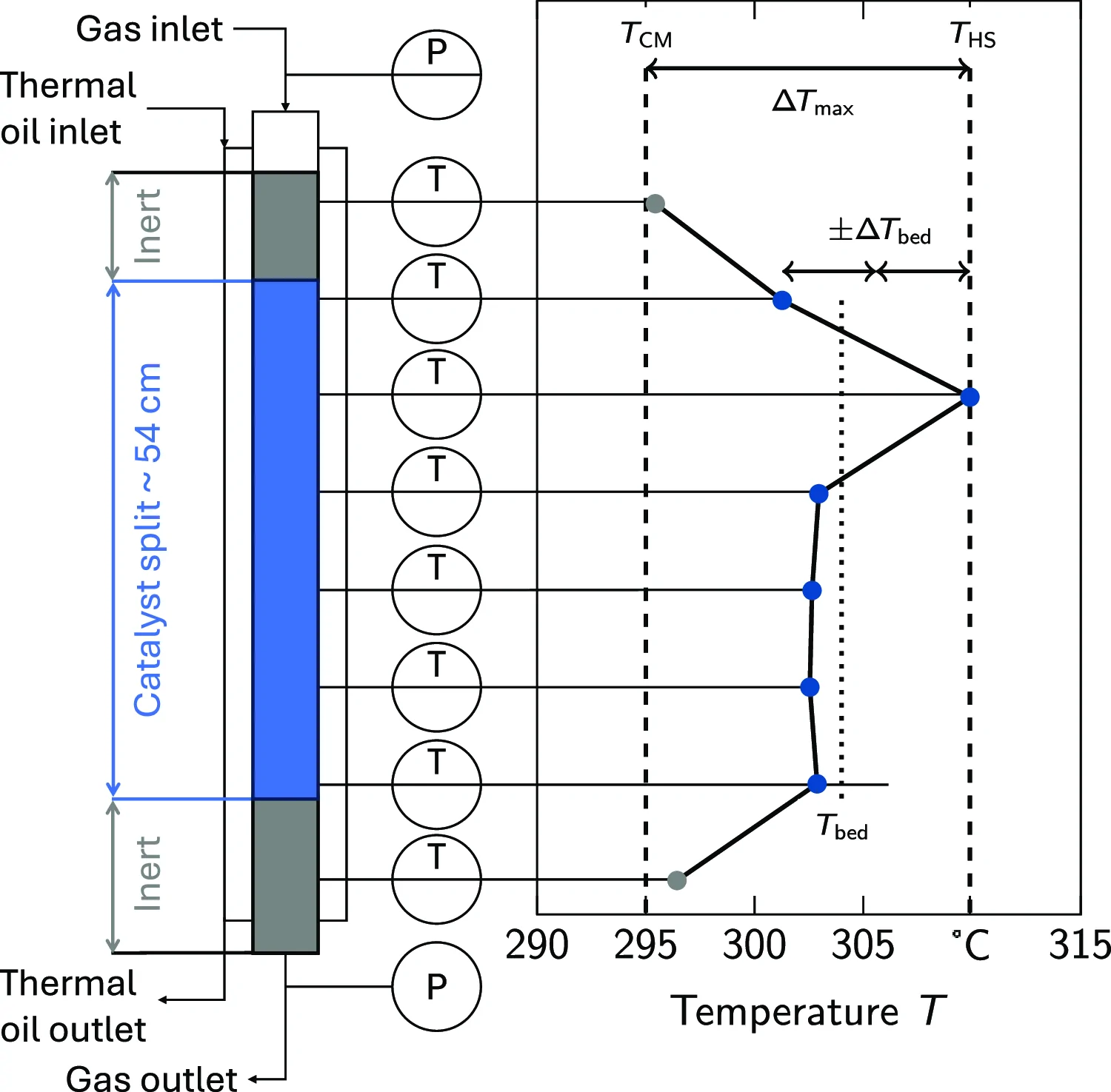
Reactor filling, instrumentation and exemplary temperature profile at *p*_in_ = 3.5 bar, *r*_O_2_/C_2_H_6__ = 0.373, *r*_H_2_O/C_2_H_6__ = 0.427, *T*_CM_ = 295 °C, WHSV = 1.5 h^–1^ and *p*_out_ = 3.2 bar for non-isothermal, process-relevant conditions and undiluted catalyst bed using Catalyst B.

The catalyst particle size fraction was selected based on hydrodynamic design considerations while maintaining an acceptable pressure drop under the investigated operating conditions. For the present reactor with an inner diameter *D*_R_ of 10 mm containing a catalyst sieve fraction with a maximum catalyst particle diameter *d*_p,max_ of 2.5 mm and a minimum catalyst particle diameter *d*_p,min_ of 1.0 mm, this corresponds to a reactor-to-particle diameter ratio in the range of

4 $$\frac{D_{R}}{d_{p,\max}}=4\leq \frac{D_{R}}{d_{p}}\leq 10=\frac{D_{R}}{d_{p,\min}}$$

Additional hydrodynamic considerations regarding catalyst particle size selection are provided in Section S1.5 (SI). In addition to the reactor-to-particle diameter ratio, the bed length-to-particle diameter ratio *L*_bed_/*d*_p_ ^52^ and particle Reynolds number *Re*_p_ ^53^ were evaluated under the representative reaction conditions. The resulting values satisfy commonly applied criteria for minimizing bed entrance and bed end effects and avoiding external convection limitations.

Although reactor-to-particle diameter ratios with the catalyst particle diameter *d*_p_ of

5 $$\frac{D_{R}}{d_{p}}\geq 8$$

are commonly preferred to minimize wall effects,^54^ the investigated particle size fraction spans reactor-to-particle diameter ratios both below and above this guideline. Consequently, a substantial fraction of the catalyst particles fulfils the recommended range. Combined with the favorable values obtained for *L*_bed_/*d*_p_ and *Re*_p_, the selected particle size fraction was considered to be acceptable for the present study.

The use of smaller catalyst particles would result in a substantial increase in pressure drop under the investigated operating conditions. Therefore, the selected particle size fraction represents a compromise between hydrodynamic performance and an acceptable pressure drop below 0.3–0.5 bar due to the pressure sensitivity of the ODHE reactions and the overall low reaction pressure regime.

It should further be noted that comparatively small reactor-to-particle diameter ratios are also encountered in industrial multitubular fixed-bed reactors. For example, reactor tube inner diameters of 15–25 mm in combination with cylindrical, hollow cylindrical, or multilobed catalyst pellets have been reported, resulting in tube-to-particle diameter ratios in the range of approximately 3–5.^55^

Consequently, the reactor-to-particle diameter ratio employed in the present laboratory reactor lies within the same order of magnitude as that found in industrial reactor designs. While deviations from ideal plug flow due to wall effects cannot be excluded, their influence was considered to be acceptable for the present study.

Depending on catalyst type and degree of inert dilution of the catalyst bed with glass beads, the respective catalyst mass was filled for each experimental series according to Table 2. With an inert dilution of 70 or 85 wt % glass beads (2.0 mm diameter), temperature deviations Δ*T*_bed_ over the catalyst bed were limited to a maximum of ±5 K or ±2 K, respectively. A temperature deviation Δ*T*_bed_ ≤ ±2 K was defined as pseudo-isothermal conditions.

**Table 2:** Reaction Conditions for Variation of Catalyst Bed Inert Dilution (Inert dil.), Steam-to-Ethane Ratio *r*_H_2_O/C_2_H_6__, Reactor Inlet Pressure *p*_in_, WHSV, and Comparison of Ethane and Ethylene Oxidation

variation	cat.	dil.	*r*_O_2_/C_2_H_n__ ^d^	*r*_H_2_O/C_2_H_n__ ^d^	*M*_cat_	*T*_CM_	*p*_in_	WHSV	Δ*T*_bed_ ^a^
		wt %			g	°C	bar	h^–1^	K
inert dil.	A	85	0.373	0.427	9	305–320	3.5	0.7–1.4	±2
		70			18	300–320		2.2–4.2	±4
		0			59	280–300		4.4–8.6	±5
*p*_in_	C	85	0.373	0.427	9	315	2.3–5.5	6.6	±2
*r*_H_2_O/C_2_H_6__	C	0	0.373	0–0.559	45.7	305	3.5	1.1–1.4^b^	±5
WHSV	B	0	0.373	0.427	39.5	275 – 305	3.5	0.8–2.1	Table 4
C_2_H_6_ ox.	A	85	0.373	0.427	9	310	3.5	6.6	±2
C_2_H_4_ ox.^c^	A	85	0.373	0.427	9	310	3.5	6.6	±1

a Pseudo-isothermal conditions: Δ*T*_bed_ ≤ ±2 K, process-relevant, non-isothermal conditions: Δ*T*_bed_ > ±2 K.

b WHSV changes implicitly with *r*_H_2_O/C_2_H_6__ since *Ṅ*_C_2_H_6__ was fixed.

c Ethylene oxidation is the only experimental series using ethylene instead of ethane as the hydrocarbon feed.

d Standard deviations are *r*_O_2_/C_2_H_n__ ± 7.5 × 10^–6^ and *r*_H_2_O/C_2_H_n__ ± 2.0 × 10^–3^.

In Figure 1, the hotspot temperature *T*_HS_ = 310 °C corresponds to a maximum temperature increase of Δ*T*_max_ = 15 K. An average catalyst bed temperature of *T*_bed_ = 303 °C and a temperature deviation over the catalyst bed of Δ*T*_bed_ = ±5 K are obtained.

### 2.4 Reaction Condition Variations

Catalyst bed inert dilution, molar steam-to-ethane ratio *r*_H_2_O/C_2_H_6__, reactor inlet pressure *p*_in_, coolant temperature *T*_CM_, and WHSV were systematically varied. The applied reaction conditions for variation of important process parameters are reported in Table 2.

A constant molar oxygen-to-hydrocarbon ratio of *r*_O_2_/C_2_H_n__ = 0.373 was applied. Reaction conditions preventing oxygen depletion of the catalyst^44^ at the end of the catalyst bed and avoiding excessively high oxygen-to-hydrocarbon ratios resulting in explosive mixtures and safety risks were applied.

The steam-to-ethane ratio *r*_H_2_O/C_2_H_6__ was varied by increasing the steam dilution stepwise at constant ethane feed flow *Ṅ*_C_2_H_6__. However, too low steam-to-ethane ratios lead to reversible catalyst deactivation (gradual decline in ethane conversion with increasing time-on-stream) due to steam depletion.^43^ For the comparison of ethane and ethylene oxidation, five runs of a standard experiment at the given conditions using ethane or ethylene as feed were performed.

### 2.5 Product Analysis

The dry product gas composition after condensation of water and acetic acid was analyzed with a two-channel microGC which separates the compounds H_2_, O_2_, N_2_, CH_4_, CO, CO_2_, C_2_H_4_, C_2_H_6_, and C_2_H_2_. Further experimental details on the microGC evaluation and calibration are given in Section S1.1 (SI).

Assuming ideal gas behavior at low pressure, ambient temperature, and high dilution with carrier gas, volume fractions φ_*i*_ obtained by gas chromatography are equivalent to the molar fractions *x*_*i*,dry_. Mass fractions *w*_*i*,dry_ of the dry product gas components *i* are calculated via molar fractions *x*_*i*,dry_ and molar masses *M̅*_*i*_ according to

6 $$w_{i,\mathrm{dry}}=\frac{x_{i,\mathrm{dry}}\cdot {\mathrm{M̅}}_{i}}{\sum_{i=0}^{m}\left(x_{i,\mathrm{dry}}\cdot {\mathrm{M̅}}_{i}\right)}$$

Acetic acid in the condensate was quantified by acid–base titration and validated by refractive index correlation. Titration was performed using 1 M sodium hydroxide solution (Titrisol, Merck Millipore) with phenolphthalein (Merck Millipore) as indicator. The refractive index *n* was measured using a digital refractometer (DR6300, Krüss) at 20 °C. The following linear correlation between refractive index *n* and acetic acid mass fraction *w*_AcOH_ was established for mass fractions *w*_AcOH_ ≤ 0.22, based on 42 calibration samples, with *R* ^2^ exceeding 0.99

7 $$n=1.333+w_{\mathrm{AcOH}}\cdot 6.591\times 10^{-4}$$

The mass flow rates *Ṁ*_*i*_ of the gaseous components *i* in the dry product gas are calculated via mass fractions *w*_*i*,dry_ resulting from the normalized dry product gas composition (eq 19) and the total dry product gas mass flow rate *Ṁ*_DPG_ according to

8 $${Ṁ}_{i}=w_{i,\mathrm{dry}}\cdot {Ṁ}_{\mathrm{DPG}}$$

The product mass flow rate *Ṁ*_AcOH_ of acetic acid is calculated via acetic acid mass fraction *w*_AcOH_ in the condensate and total condensate mass flow rate *Ṁ*_cond_ according to

9 $${Ṁ}_{\mathrm{AcOH}}=w_{\mathrm{AcOH}}\cdot {Ṁ}_{\mathrm{cond}}$$

Molar flow rates *Ṅ*_*i*_ of all components *i* in the wet product gas are calculated via mass flow rates *Ṁ*_*i*_ in dry product gas and condensate and molar masses *M̅*_*i*_ according to

10 $${Ṅ}_{i}=\frac{{Ṁ}_{i}}{{\mathrm{M̅}}_{i}}$$

Molar fractions *x*_*i*_ of the wet product gas components *i* are calculated via molar flow rates *Ṅ*_*i*_ in the wet product gas according to

11 $$x_{i}=\frac{\dot{N_{i}}}{\sum_{i=0}^{m}\dot{N_{i}}}$$

The partial pressure *p*_*i*,effluent_ of component *i* in the wet product gas is calculated via reactor outlet pressure *p*_out_ and molar fraction *x*_*i*_ in the wet product gas according to

12 $$p_{i,\mathrm{effluent}}=x_{i}\cdot p_{\mathrm{out}}$$

Molar balances and mass balances were verified for each experiment to ensure data consistency. Mass balances and molar carbon, oxygen, and hydrogen balances closed within a maximum deviation of 2%. The mass balance is calculated using the feed mass flow rates *Ṁ*_*m*_ of feed components *m* and product mass flow rates *Ṁ*_*l*_ of product components *l* according to

13 $$\Delta M=\frac{\sum_{i=0}^{l}{Ṁ}_{l}}{\sum_{i=0}^{m}{Ṁ}_{m}}$$

Molar balances Δ*N*_*e*_ of element *e* are calculated as the ratio of feed molar flow rates *Ṅ*_*m*_ of feed components *m* and product molar flow rates *Ṅ*_*l*_ of product components *l* containing element *e* with the corresponding stoichiometry factor ν_*e*_ according to

14 $$\Delta N_{e}=\frac{\sum_{i=0}^{l}\left({Ṅ}_{l}\cdot \nu_{e,l}\right)}{\sum_{i=0}^{m}\left({Ṅ}_{m}\cdot \nu_{e,m}\right)}$$

The hydrocarbon conversion *X*_C_2_H_n__ is calculated using molar flow rates *Ṅ*_*i*_ of all wet product gas compounds *i* (including unreacted hydrocarbon) and their corresponding carbon stoichiometry factors ν_c,_ *_i_* according to

15 $$X_{C_{2}H_{n}}=\frac{\sum_{i=0}^{m}\left({Ṅ}_{i}\cdot \nu_{c,i}\right)-{Ṅ}_{C_{2}H_{n}}\cdot \nu_{c,C_{2}H_{n}}}{\sum_{i=0}^{m}\left({Ṅ}_{i}\cdot \nu_{c,i}\right)}$$

The selectivity of product *j* is calculated using molar flow rates *Ṅ*_*i*_ of all wet product gas compounds *i* (excluding unreacted hydrocarbon) and their corresponding carbon stoichiometry factors ν_c,*i*_ according to

16 $$S_{j}=\frac{{Ṅ}_{j}\cdot \nu_{c,j}}{\sum_{i=0}^{m}\left({Ṅ}_{i}\cdot \nu_{c,i}\right)-{Ṅ}_{C_{2}H_{n}}\cdot \nu_{c,C_{2}H_{n}}}$$

The combination of ethylene selectivity *S*_C_2_H_4__ and acetic acid selectivity *S*_AcOH_ to the commercial product selectivity *S*_CP_ yields another important catalytic performance indicator for the ODHE process and is calculated according to

17 $$S_{\mathrm{CP}}=S_{C_{2}H_{4}}+S_{\mathrm{AcOH}}$$

The carbon oxide selectivity *S*_CO_*x*__ is calculated as a combination of carbon monoxide selectivity *S*_CO_ and carbon dioxide selectivity *S*_CO_2__ according to

18 $$S_{{\mathrm{CO}}_{x}}=S_{\mathrm{CO}}+S_{{\mathrm{CO}}_{2}}$$

Product yields *Y*_*i*_ are calculated from the corresponding product selectivities *S*_*i*_ and hydrocarbon conversion *X*_C_2_H_n__ according to

19 $$Y_{i}=X_{C_{2}H_{n}}\cdot S_{i}$$

The molar product ratio *r*_CP_ of ethylene to acetic acid can be seen as an additional crucial catalytic performance indicator in combination with the commercial product selectivity *S*_CP_. The molar product ratio *r*_CP_ is the ratio of the individual main product selectivities *S*_C_2_H_4__ and *S*_AcOH_ at a specific balance point according to

20 $$r_{\mathrm{CP}}=\frac{{Ṅ}_{C_{2}H_{4}}}{{Ṅ}_{\mathrm{AcOH}}}=\frac{S_{C_{2}H_{4}}}{S_{\mathrm{AcOH}}}$$

The ratio of carbon oxide selectivities *S*_CO_ and *S*_CO_2__ yields the molar product ratio *r*_CO_*x*__ according to

21 $$r_{{\mathrm{CO}}_{x}}=\frac{{Ṅ}_{\mathrm{CO}}}{{Ṅ}_{{\mathrm{CO}}_{2}}}=\frac{S_{\mathrm{CO}}}{S_{{\mathrm{CO}}_{2}}}$$

Details on the calculation of conversion and selectivity are provided in Section S1.2 (SI), the methodology for assessing measurement accuracy is described in Section S1.4 (SI).

## 3 Results

This section presents the results of process parameter variations conducted to assess the influences on catalytic performance in ODHE. The results combine experiments under pseudo-isothermal and non-isothermal conditions to bridge the gap between laboratory-scale investigations and industrial applications. Error bars are determined according to Section S1.4 (SI).

### 3.1 Comparison of Pseudo-Isothermal and Process-Relevant Conditions

To demonstrate that the observed trends in product selectivities *S*_*i*_, product yields *Y*_*i*_ and molar product ratios *r*_*i*_ are independent of the thermal regime, a variation of the catalyst bed inert dilution was performed using Catalyst A. This approach enabled systematic adjustment of the temperature deviation Δ*T*_bed_ over the catalyst bed and covered a broad range of WHSV values by varying catalyst mass *M*_cat_ and feed mass flow rate *Ṁ*_feed_.

Low values of WHSV ≤ 1.4 h^–1^ correspond to process-relevant, non-isothermal conditions, and high values of WHSV ≥4.4 h^–1^ correspond to pseudo-isothermal conditions. Intermediate values of WHSV in the range of 2.2–4.2 h^–1^ correspond to non-isothermal conditions with lower temperature deviation over the catalyst bed than for process-relevant, non-isothermal conditions, according to Table 2 (variation of Inert dil.).

The commercial product selectivity *S*_CP_ and commercial product yield *Y*_CP_ over varied ethane conversion *X*_C_2_H_6__ are shown in Figure 2. Different marker shapes indicate catalyst bed dilution (temperature deviation over the catalyst bed), while color and filling represent variations in the WHSV.

**Figure 2:**
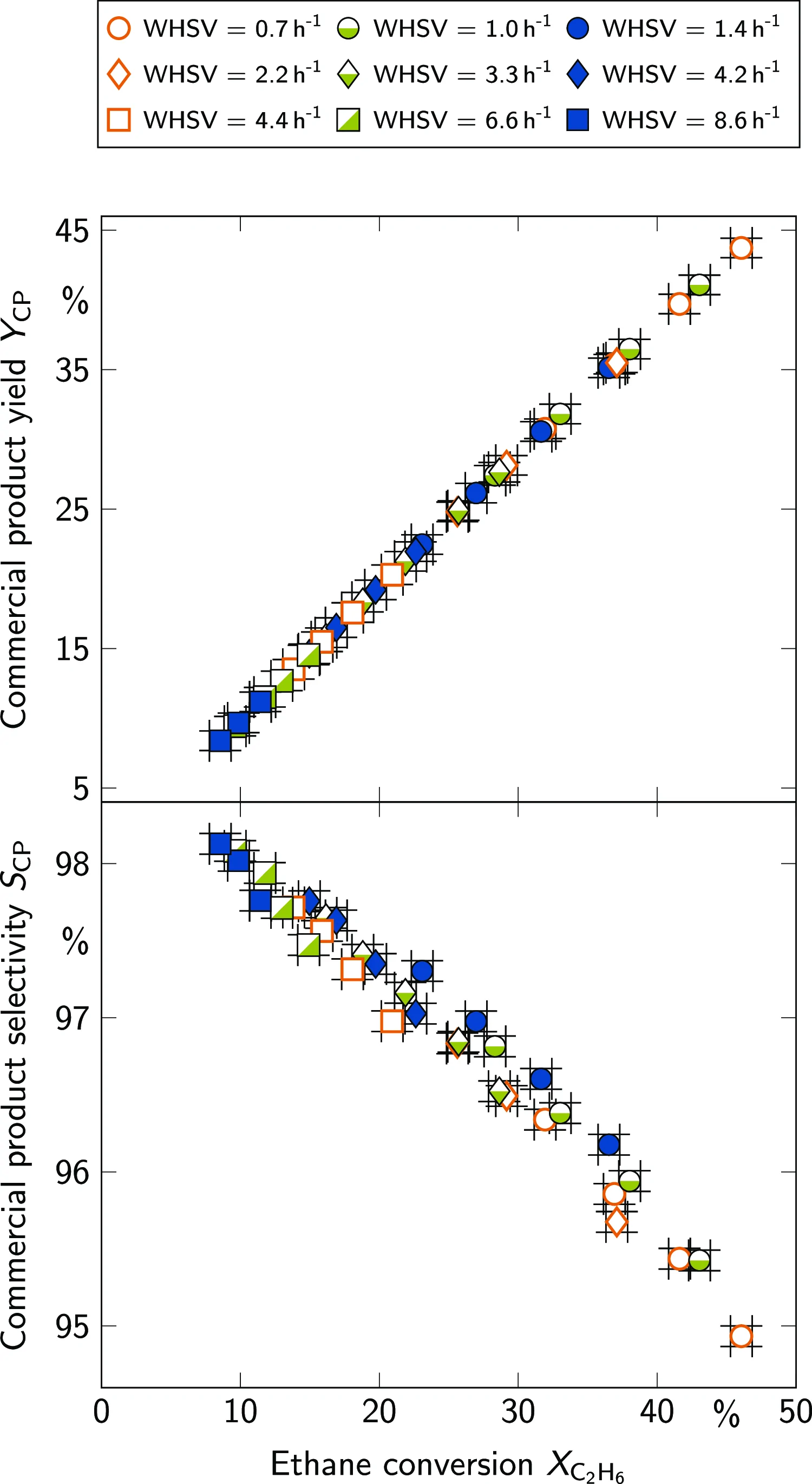
Dependency of *S*_CP_ (bottom panel) and *Y*_CP_ (top panel) on coolant temperature *T*_CM_ and WHSV at *p*_in_ = 3.5 bar, *r*_O_2_/C_2_H_6__ = 0.373, *r*_H_2_O/C_2_H_6__ = 0.427, *T*_CM_ = 300–320 °C using Catalyst A. Catalyst bed inert dilution of 0, 70, and 85 wt % correspond to WHSV = 0.7–1.4 h^–1^, WHSV = 2.2–4.2 h^–1^ and WHSV = 4.4–8.6 h^–1^, respectively.

The commercial product selectivity *S*_CP_ decreases with an increase in ethane conversion *X*_C_2_H_6__. The commercial product yield *Y*_CP_ increases linearly with ethane conversion *X*_C_2_H_6__. The increase in ethane conversion *X*_C_2_H_6__ overcompensates for the simultaneous decline in commercial product selectivity *S*_CP_.

These trends are fundamental for both pseudo-isothermal and non-isothermal conditions, demonstrating that the correlation between ethane conversion *X*_C_2_H_6__ and commercial product selectivity *S*_CP_ and commercial product yield *Y*_CP_ is independent of the catalyst bed inert dilution, thus the thermal regime in the catalyst bed.

These findings indicate that, within the tested range of parameters, the choice between pseudo-isothermal and non-isothermal operation has no significant impact on the trends of performance indicators in ODHE. Detailed data for individual product selectivities *S*_*i*_, product yields *Y*_*i*_ and molar product ratios *r*_*i*_ for variation of catalyst bed inert dilution *M*_cat_ is provided in Section S2.1 (SI). Section 3.4 provides a detailed discussion of observed trends in individual product selectivities *S*_*i*_ with varied coolant temperature *T*_CM_ and WHSV.

### 3.2 Effect of Reactor Inlet Pressure

Variation of the reactor inlet pressure *p*_in_ in ODHE is analyzed under the reported reaction conditions according to Table 2 (variation of *p*_in_).

Although WHSV was kept constant during the pressure variation, changing the reactor inlet pressure *p*_in_ affects the volumetric gas flow rate under reaction conditions and, therefore, the residence time. Thus, the pressure variation cannot be interpreted solely as a change in partial pressures but also includes a residence-time-related contribution.

To decouple these effects as far as possible without varying multiple operating parameters simultaneously, the results are additionally discussed as a function of a modified reciprocal gas hourly space velocity GHVS_mod_^–1^. This representation is used only as an interpretative measure, while WHSV was the experimentally controlled space-velocity parameter.

Figure 3 shows the influence of reactor inlet pressure *p*_in_ on ethane conversion *X*_C_2_H_6__, apparent reaction rate constant *k*, and product distribution. A derivation of the determination of the apparent reaction rate constant *k* is given in Section S1.3 (SI).

**Figure 3:**
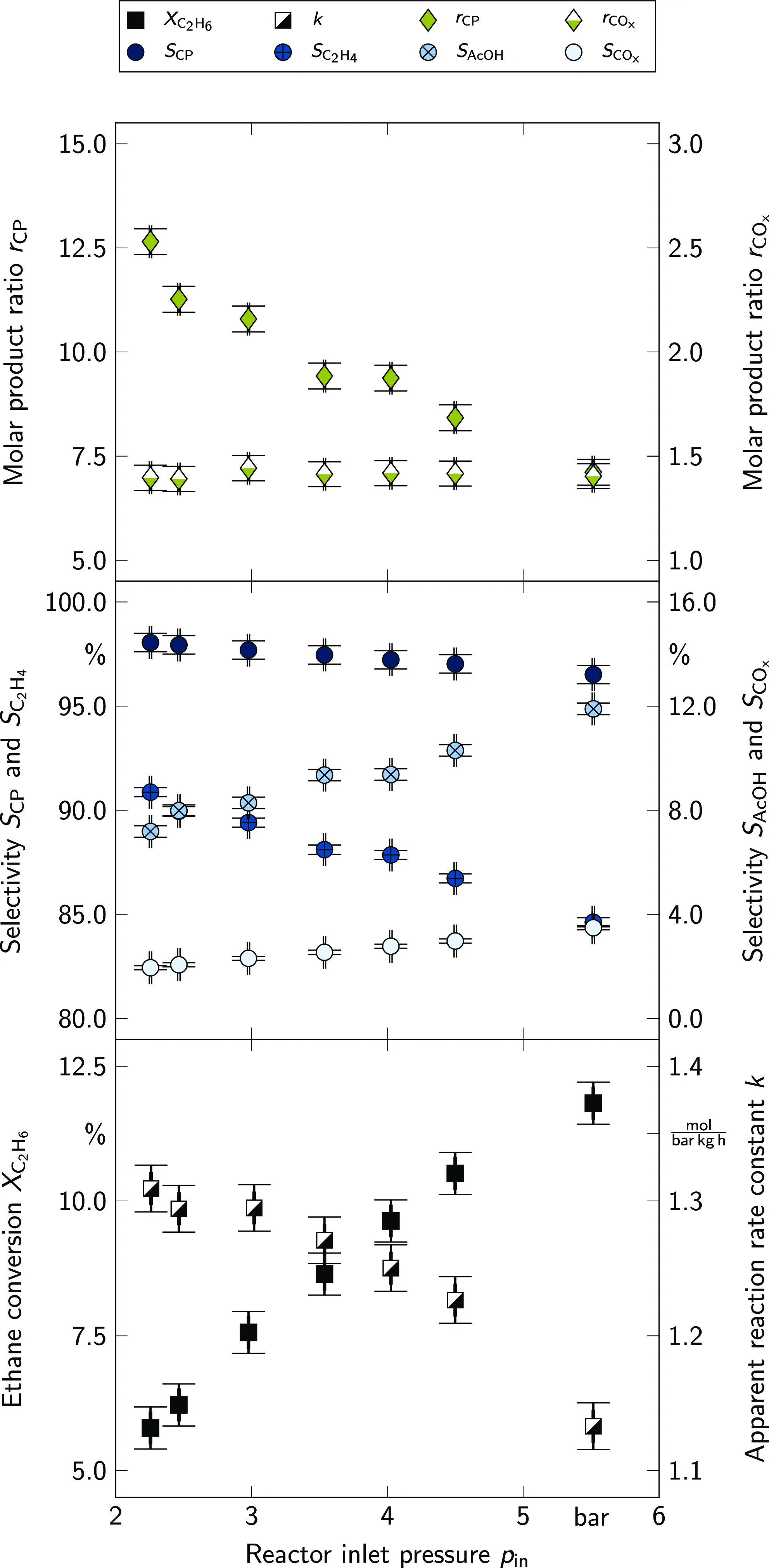
Dependency of *X*_C_2_H_6__ and *k* (bottom panel), *S*_*i*_ (middle panel), and *r*_*i*_ (top panel) on the reactor inlet pressure *p*_in_ at *r*_O_2_/C_2_H_6__ = 0.373, *r*_H_2_O/C_2_H_6__ = 0.427, *T*_CM_ = 315 °C, WHSV = 6.6 h^–1^ with 85 wt % inert dilution of the catalyst bed using Catalyst C.

Increasing the reactor inlet pressure *p*_in_ increases ethane conversion *X*_C_2_H_6__, reflecting the effect of higher partial pressures of feed components, which enhance the overall reaction rate, and increased residence time due to elevated pressure. Interestingly, the apparent reaction rate constant *k* is constant for low reactor inlet pressure, but decreases with increasing reactor inlet pressure *p*_in_ once the steam partial pressure in the reactor feed exceeds *p*_H_2_O,feed_ ≈ 0.75 bar. This corresponds to a reactor inlet pressure of *p*_in_ ≈ 3 bar.

Evaluating the apparent reaction rate constant *k* removes the influence of the residence time, enabling a more direct assessment of pressure effects. However, it should be noted that the calculated reaction rate constant represents an effective parameter derived from the overall ethane conversion based on simplified kinetic assumptions and does not correspond to intrinsic kinetics derived from the elementary reaction mechanism, as shown in Section S1.3 (SI). Consequently, the apparent reaction rate constant *k* should be interpreted as a measure of the overall catalyst activity rather than a true intrinsic rate constant.

The decrease in apparent reaction rate constant *k* with increasing reactor inlet pressure *p*_in_ exceeding a steam partial pressure of *p*_H_2_O,feed_ ≥ 0.75 bar suggests that the observed pressure dependency cannot be attributed solely to the first-order approximation underlying the derivation of the apparent reaction rate constant *k*. A plausible explanation is the simultaneous increase in steam partial pressure *p*_H_2_O_.

Inhibitory effects of steam have been reported for related Mo–V–Nb-based mixed oxide catalysts, including MoVNbPdO_*x*_ systems^56^ and MoVNbO_*x*_ materials.^27^ Although the catalyst investigated in the present work contains tellurium instead of palladium, it is based on the same M1-type Mo–V–Nb oxide framework that governs the catalytic performance. Therefore, a qualitatively similar interaction of steam with active surface sites appears to be plausible.

Consistently, the apparent reaction rate constant *k* exhibits a similar decline when plotted as a function of steam partial pressure, as shown in Section S2.2 (SI), indicating partial inhibition by adsorbed steam under the investigated conditions.

With increasing reactor inlet pressure *p*_in_, ethylene selectivity *S*_C_2_H_4__ decreases, while acetic acid selectivity *S*_AcOH_ increases, indicating a shift toward oxygenate (organic compounds containing oxygen) formation. Since the ethane conversion varied alongside the change in the reactor inlet pressure *p*_in_, an overlap of effects from multiple parameters is possible.

The observation of increasing acetic acid selectivity *S*_AcOH_ with increasing reactor inlet pressure *p*_in_ is qualitatively consistent with literature reports describing enhanced acetic acid formation at elevated steam partial pressure.^27,57^ However, these studies varied the steam partial pressure while maintaining constant ethane and oxygen partial pressures, whereas in the present work, an increase in reactor inlet pressure *p*_in_ results in a simultaneous increase of all partial pressures *p*_*i*_.

Increasing the reactor inlet pressure *p*_in_ inherently raises the steam partial pressure *p*_H_2_O_ in the reactor, and thus the water coverage of the catalyst surface. The influence of steam partial pressure *p*_H_2_O_ and degree of coverage θ_H_2_O_ of the surface with water will further be discussed in Section 3.3.

Carbon oxide selectivity *S*_CO_*x*__ increases with reactor inlet pressure *p*_in_, which can be attributed to the elevated partial pressure of oxygen enhancing total oxidation reactions but is superimposed with effects of varied ethane conversion. Furthermore, a higher reactor inlet pressure *p*_in_ extends the residence time in the reactor, thereby promoting secondary oxidation of ethylene and acetic acid to carbon oxides. The commercial product selectivity *S*_CP_ declines at a higher reactor inlet pressure *p*_in_.

The molar product ratio *r*_CP_ of ethylene to acetic acid decreases with an increasing reactor inlet pressure *p*_in_, driven by the relative promotion of acetic acid formation. In contrast, the molar ratio *r*_CO_*x*__ remains nearly constant under the investigated conditions. These findings underline that higher reactor inlet pressures *p*_in_ enhance ethane conversion *X*_C_2_H_6__ but compromise commercial product selectivity *S*_CP_ and decrease the molar product ratio *r*_CP_.

To assess whether the observed changes in product selectivity resulting from variations in reactor inlet pressure *p*_in_ could also be attributed solely to changes in WHSV, the sensitivity of the product selectivities to both reactor inlet pressure and WHSV is compared via a modified reciprocal gas hourly space velocity GHSV_mod_^–1^ (further details in Section S2.2 (SI)). Table 3 reports the slope of the linear regression of ethane conversion and product selectivity with modified reciprocal gas hourly space velocity GHSV_mod_^–1^ for both experimental series (variation of Inert dil., Section S2.1 (SI), and variation of *p*_in_). Regarding catalyst bed inert dilution variation, only experiments conducted under equal reaction conditions as for variation of reactor inlet pressure are considered.

**Table 3:** Slopes of Linear Regression of Ethane Conversion *X*_C_2_H_6__ and Product Selectivities *S*_*i*_ with Modified Reciprocal Gas Hourly Space Velocity GHSV_mod_^–1^ for Two Experimental Series (Variation of Inert dil., Section S2.1 (SI), and Variation of *p*_in_)­^a^

slope	*X*_C_2_H_6__	*S*_CP_	*S*_C_2_H_4__	*S*_AcOH_
	$\mathrm{mol}-\%\cdot \frac{L_{\mathrm{gas}}}{{\mathrm{skg}}_{\mathrm{act}}}$			
*p*_in_	12.7	–2.5	–9.8	7.2
inert dil.	12.7	–1.0	–1.1	0.1

a Regarding catalyst bed inert dilution variation, experiments with an inert dilution of 85 wt % of the catalyst bed and a coolant temperature *T*_CM_ of 315 °C are considered.

The comparison shows that variations in reactor inlet pressure and WHSV exhibit an identical influence on ethane conversion, as both parameters affect ethane conversion solely through their impact on the modified reciprocal gas hourly space velocity GHSV_mod_^–1^. However, the reactor inlet pressure shows a significantly stronger effect on product selectivities, which can be attributed to a significant increase in steam partial pressure *p*_H_2_O,effluent_ in the wet product gas with higher reactor inlet pressure *p*_in_. In contrast, during WHSV variation the steam partial pressure changes only marginally caused by varied ethane conversion.

### 3.3 Effect of the Steam-to-Ethane Ratio

Variation of the molar steam-to-ethane ratio *r*_H_2_O/C_2_H_6__ by increasing the steam dilution stepwise at constant ethane feed flow *Ṅ*_C_2_H_6__ is analyzed under the reported reaction conditions according to Table 2 (variation of *r*_H_2_O/C_2_H_6__). Figure 4 illustrates the influence of molar steam-to-ethane ratio *r*_H_2_O/C_2_H_6__ on ethane conversion *X*_C_2_H_6__, apparent reaction rate constant *k*, and product selectivities *S*_*i*_ and product yields *Y*_*i*_.

**Figure 4:**
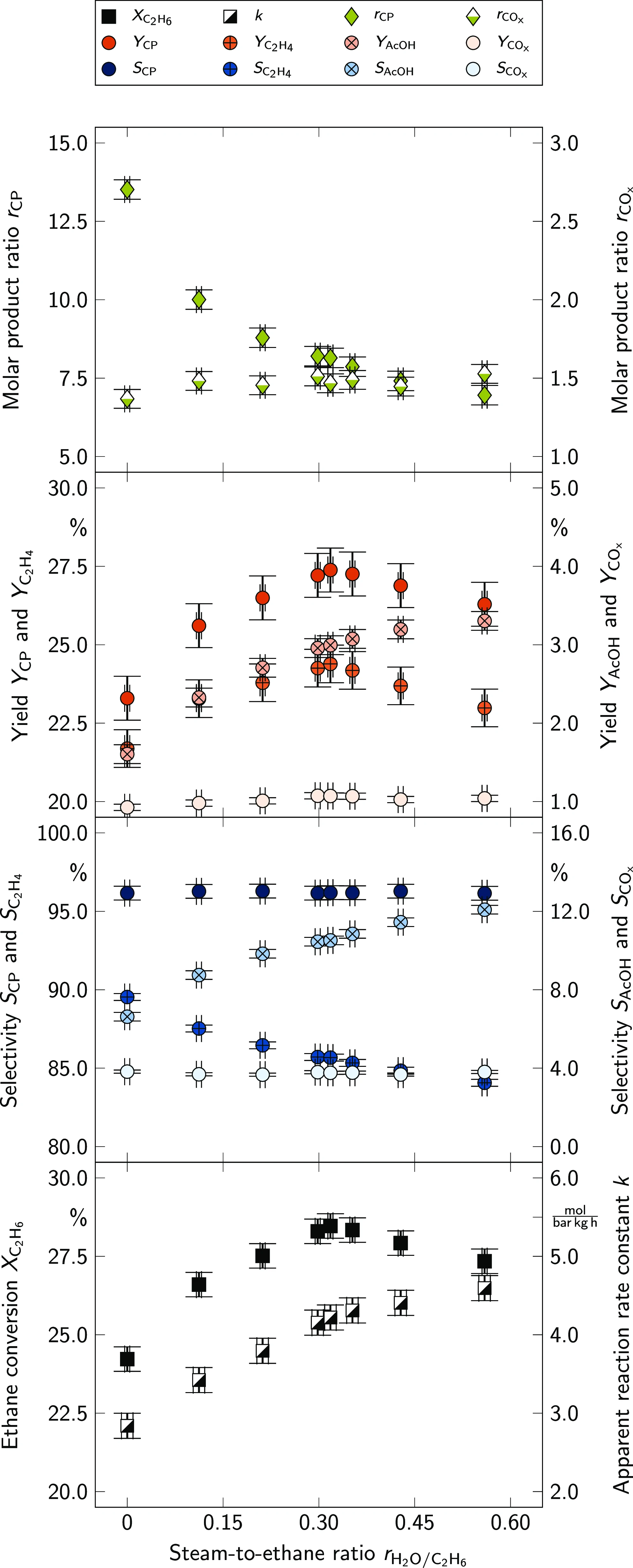
Dependency of *X*_C_2_H_6__ and *k* (bottom panel), *S*_*i*_ (bottom middle panel), *Y*_*i*_ (top middle panel), and *r*_*i*_ (top panel) on the molar steam-to-ethane ratio *r*_H_2_O/C_2_H_6__ at *p*_in_ = 3.5 bar, *r*_O_2_/C_2_H_6__ = 0.373, *T*_CM_ = 305 °C, WHSV = 1.1–1.4 h^–1^ with undiluted catalyst bed using Catalyst C.

As stepwise addition of steam to the feed increases the WHSV and accordingly decreases the space time, the ethane conversion is expected to diminish, as well. Interestingly, increasing the steam-to-ethane ratio *r*_H_2_O/C_2_H_6__ first leads to an increase in ethane conversion *X*_C_2_H_6__, reaching a maximum at a steam-to-ethane ratio of *r*_H_2_O/C_2_H_6__ = 0.32.

For steam-to-ethane ratios *r*_H_2_O/C_2_H_6__ < 0.32, a slow, reversible deactivation (gradual decline in ethane conversion with increasing time-on-stream) of the MoVNbTeO_*x*_ catalyst was observed, consistent with literature reports on steam-deficient conditions,^43^ preventing higher ethane conversion *X*_C_2_H_6__ despite longer space time, due to site blocking by oxygenate species.

At higher steam addition, ethane conversion *X*_C_2_H_6__ slightly decreases, although the apparent reaction rate constant *k* continues to increase. This behavior indicates that steam cannot be considered an inert diluent. Instead, increasing the steam-to-ethane ratio *r*_H_2_O/C_2_H_6__ simultaneously increases the steam partial pressure *p*_H_2_O_, while decreasing the partial pressures of ethane and oxygen due to the constant reactor inlet pressure.

The apparent reaction rate constant *k* does not exhibit a decline beyond the ethane conversion maximum. Instead, the apparent reaction rate constant *k* continues to increase with further steam addition, though with a distinctly reduced slope beyond *r*_H_2_O/C_2_H_6__ ≈ 0.32. This change in behavior coincides with the onset of decreasing ethane conversion *X*_C_2_H_6__ and may indicate that inhibitory effects become increasingly relevant at elevated steam partial pressures *p*_H_2_O_. Similar inhibitory effects of steam on Mo–V–Nb-based mixed oxide catalysts have been reported in the literature and were attributed to increased surface coverage by adsorbed water species.^56^

Consistently, the apparent reaction rate constant *k* exhibits a similar dependence when plotted as a function of steam partial pressure *p*_H_2_O_ in the feed gas, as shown in Section S2.2 (SI). While increasing the steam partial pressure continues to promote the apparent overall catalyst activity, the reduced slope of the apparent reaction rate constant *k* suggests a partial inhibition by adsorbed water species at elevated steam partial pressures.

The increase in apparent reaction rate constant *k* with higher steam partial pressure *p*_H_2_O_ could be attributed to the influence of co-adsorbed water on product desorption and active site availability. Higher *p*_H_2_O_ may facilitate the desorption of chemisorbed oxygenated species such as acetic acid via hydrolysis of oxygenate species with co-adsorbed water, thereby accelerating product removal from the surface and preventing site blocking. As a result, a larger fraction of active sites remains available for reactant adsorption, increasing the apparent reaction rate constant *k*.

The reduced slope of the apparent reaction rate constant *k* for exceeding a steam partial pressure of *p*_H_2_O,feed_ ≥ 0.75 bar (corresponds to a *r*_H_2_O/C_2_H_6__ ≈ 0.32), however, may be explained by inhibiting effects of the reaction pathway toward ethylene (refer also to discussion on the product yields below and to Section S2.2 (SI)).

It should be noted that the experiments conducted for varying the steam-to-ethane ratio *r*_H_2_O/C_2_H_6__ were non-isothermal. Therefore, the determination of an apparent reaction rate constant *k* under these conditions must be interpreted with caution, as temperature deviations of Δ*T*_bed_ = ±5 K around the average catalyst bed temperature may influence the apparent reaction rate constant.

This limitation applies specifically to the quantitative interpretation of the apparent reaction rate constant *k* and does not affect the observed trends in the conversion and selectivity. The latter are discussed independently and are consistently observed under both kinetically oriented and process-relevant operating conditions.

In addition to the standard deviation obtained from repeated measurements of the standard experiment, the influence of Δ*T*_bed_ = ±5 K on the apparent reaction rate constant *k* was considered via error propagation. Experiments were conducted under process-relevant conditions reaching technically relevant ethane conversion and were therefore not intended as intrinsic kinetic measurements.

The ethylene selectivity *S*_C_2_H_4__ shows a continuous decrease with an increasing molar steam-to-ethane ratio *r*_H_2_O/C_2_H_6__, whereas acetic acid selectivity *S*_AcOH_ increases correspondingly. This trend indicates that steam enhances the reaction pathways for oxygenate production. The commercial product selectivity *S*_CP_ remains nearly constant within the studied range, indicating that changes in individual product selectivities *S*_*i*_ reflect a redistribution between ethylene and acetic acid formation.

Despite the continuous decline in ethylene selectivity *S*_C_2_H_4__, the ethylene yield *Y*_C_2_H_4__ increases initially and reaches a maximum at a molar steam-to-ethane ratio of *r*_H_2_O/C_2_H_6__ = 0.32. In this regime, the increase in ethane conversion *X*_C_2_H_6__ overcompensates for the loss in ethylene selectivity. At higher steam-to-ethane ratios, however, the declining ethylene selectivity and the reduction in space time lead to a decrease in ethylene yield *Y*_C_2_H_4__.

In contrast, the acetic acid yield *Y*_AcOH_ increases continuously over the entire investigated range of *r*_H_2_O/C_2_H_6__. This behavior indicates that the promoting effect of steam on acetic acid formation outweighs the adverse effect of a decrease in ethane conversion.

Moreover, the opposite trends observed for *Y*_C_2_H_4__ and *Y*_AcOH_ at elevated steam-to-ethane ratios suggest that increasing the steam partial pressure affects the pathways leading to ethylene and acetic acid formation differently. While pathways contributing to ethylene formation appear to be inhibited or become less favorable, pathways leading to acetic acid formation are further promoted.

This behavior may also contribute to the observed change in slope of the apparent reaction rate constant *k* at *r*_H_2_O/C_2_H_6__ ≈ 0.32. Since the apparent reaction rate constant *k* represents an overall activity measure reflecting the combined contribution of multiple reaction pathways, a reduced contribution from pathways leading to ethylene formation may be partially compensated by increased contributions from pathways leading to acetic acid or other products. Consequently, changes in individual pathway activities may not be directly reflected by the overall apparent reaction rate constant *k*.

The commercial product yield *Y*_CP_ exhibits a maximum at intermediate steam-to-ethane ratios. Since the commercial product selectivity *S*_CP_ remains nearly constant, the decrease in *Y*_CP_ at higher steam-to-ethane ratios directly reflects the decline in ethane conversion *X*_C_2_H_6__. Although the continued increase in *Y*_AcOH_ partly compensates for the loss in ethylene yield, the reduction in *Y*_C_2_H_4__ dominates, owing to the substantially higher contribution of ethylene to the commercial product yield.

Since the ethane conversion varied alongside the change in the molar steam-to-ethane ratio *r*_H_2_O/C_2_H_6__, an overlap of effects from multiple parameters is possible. Increasing the steam-to-ethane ratio *r*_H_2_O/C_2_H_6__ at constant molar feed flow *Ṅ*_C_2_H_6__ of ethane results in increased WHSV and decreased space time. Decreased space time should favor the primary product ethylene and limit acetic acid formation; however, the increased steam partial pressure *p*_H_2_O,effluent_ in the wet product gas appears to counteract this effect and favor acetic acid formation.

Variation of the steam-to-ethane ratio *r*_H_2_O/C_2_H_6__ does not show a significant effect on the carbon oxide selectivity *S*_CO_*x*__ and carbon oxide yield *Y*_CO_*x*__. The molar product ratio *r*_CP_ of ethylene to acetic acid decreases with an increasing steam-to-ethane ratio, whereas the molar product ratio *r*_CO_*x*__ remains unaffected across the investigated range of the molar steam-to-ethane ratio *r*_H_2_O/C_2_H_6__.

As described in the literature,^57^ the molar product ratio *r*_CP_ of ethylene to acetic acid mainly depends on the steam partial pressure *p*_H_2_O,effluent_ in the wet product gas, and not the molar steam-to-ethane ratio *r*_H_2_O/C_2_H_6__. This correlation becomes particularly relevant under integral reactor operation at high ethane conversion, *X*_C_2_H_6__, where reactant concentrations and partial pressures vary along the catalyst bed. Water formed during the reaction increases the steam partial pressure *p*_H_2_O_ downstream in the catalyst bed, shifting the selectivity toward acetic acid.

The molar product ratio *r*_CP_ of ethylene to acetic acid decreases with increasing steam partial pressure *p*_H_2_O,effluent_ in the wet product gas, as shown in Figure 5 for variation of the molar steam-to-ethane ratio *r*_H_2_O/C_2_H_6__.

**Figure 5:**
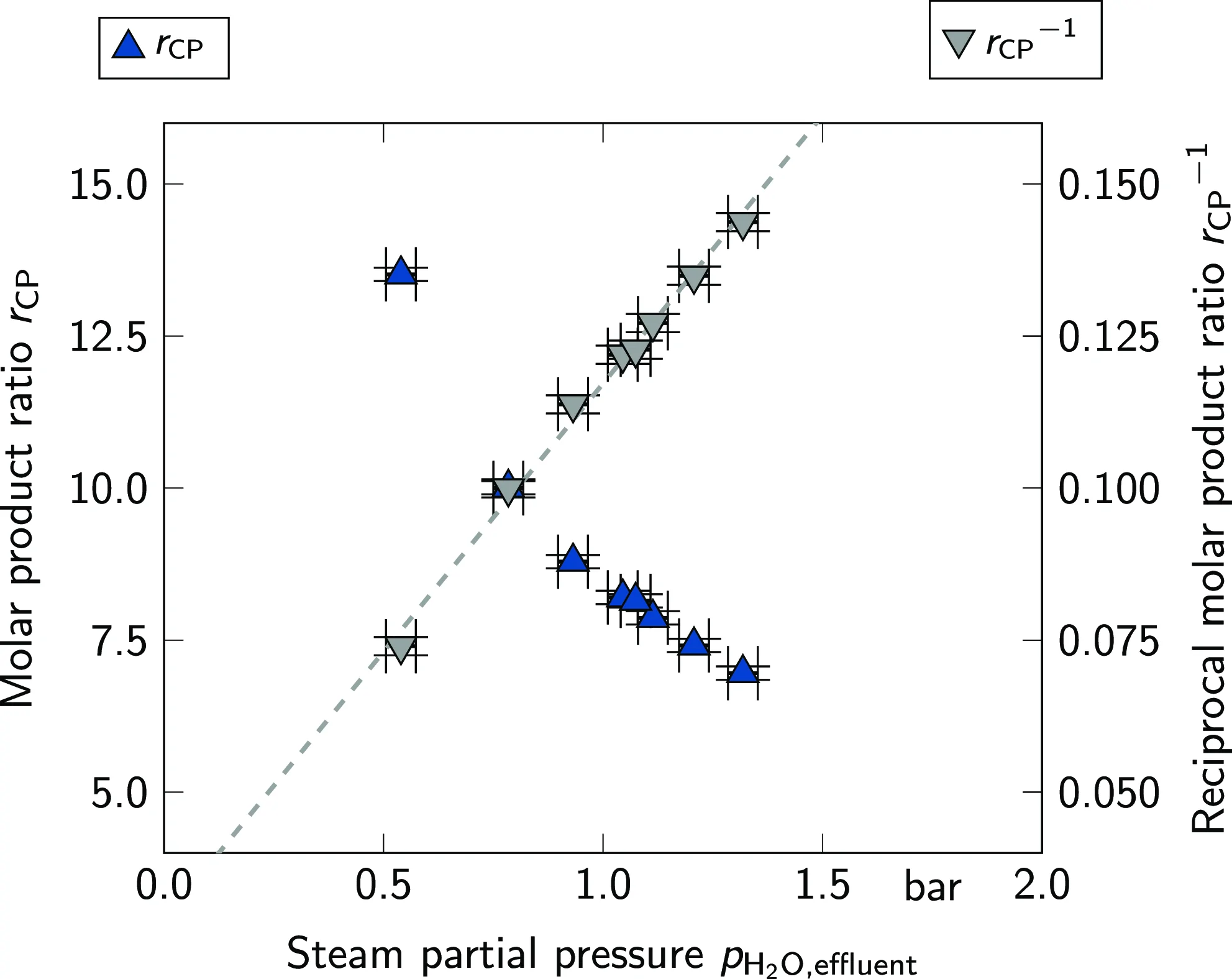
Dependency of the molar product ratio *r*_CP_ and reciprocal molar product ratio *r*_CP_^–1^ on the steam partial pressure *p*_H_2_O,effluent_ in the wet product gas at *p*_in_ = 3.5 bar, *r*_O_2_/C_2_H_6__ = 0.373, *T*_CM_ = 305 °C, WHSV = 1.1–1.4 h^–1^ with undiluted catalyst bed using Catalyst C. Linear regression for reciprocal molar product ratio *r*_CP_^–1^ with steam partial pressure *p*_H_2_O,effluent_ with *R* ^2^ exceeding 0.99.

The reciprocal molar product ratio *r*_CP_^–1^ of acetic acid to ethylene increases linearly with the steam partial pressure *p*_H_2_O,effluent_ in the wet product gas. Variations in reactor inlet pressure *p*_in_ in Section 3.2 would show a similar linear trend in the reciprocal molar product ratio *r*_CP_^–1^ with respect to steam partial pressure *p*_H_2_O,effluent_ in the wet product gas. However, due to different thermal regimes and varied space time via differing WHSV, the experimental series are not directly comparable.

Linear regression of the reciprocal molar product ratio *r*_CP_^–1^ with steam partial pressure *p*_H_2_O,effluent_ indicates that acetic acid forms on active sites requiring adsorbed water, whereas ethylene-selective sites operate independently of co-adsorbed steam. As the steam partial pressure *p*_H_2_O,effluent_ increases, a larger fraction of surface sites becomes covered with water, thereby promoting the water-assisted reaction pathway leading to acetic acid. This interpretation is consistent with a Langmuir-type adsorption model with *K* as the adsorption constant of water, in which the degree of coverage θ_H_2_O_ of the surface with water can be calculated via steam partial pressure *p*_H_2_O_ and the adsorption constant *K* according to

22 $$\theta_{H_{2}O}=\frac{K\cdot p_{H_{2}O}}{1+K\cdot p_{H_{2}O}}$$

The degree θ_free_ of free surface can be calculated according to

23 $$\theta_{\mathrm{free}}=\frac{1}{1+K\cdot p_{H_{2}O}}$$

Assuming that ethylene formation proceeds over free active sites, i.e., *S*_C_2_H_4__ ∝ θ_free_, while acetic acid formation requires co-adsorbed steam on the catalyst surface, i.e., *S*_AcOH_ ∝ θ_H_2_O_, the molar product ratio *r*_CP_ can be calculated according to

24 $$r_{\mathrm{CP}}=\frac{S_{C_{2}H_{4}}}{S_{\mathrm{AcOH}}}\propto \frac{\theta_{\mathrm{free}}}{\theta_{H_{2}O}}=\frac{1}{K\cdot p_{H_{2}O}}$$

Thus, the reciprocal molar product ratio *r*_CP_^–1^ of acetic acid to ethylene corresponds to

25 $$r_{\mathrm{CP}}^{-1}\propto K\cdot p_{H_{2}O}$$

The experimentally observed linear correlation between reciprocal molar product ratio *r*_CP_^–1^ and steam partial pressure *p*_H_2_O,effluent_ therefore provides strong evidence that acetic acid formation requires active sites occupied by adsorbed water, in contrast to ethylene formation.

The linear increase in reciprocal molar product ratio *r*_CP_^–1^ of ethylene and acetic acid with steam partial pressure *p*_H_2_O,effluent_ in the effluent provides a mechanistic basis consistent with surface coverage-dependent behavior and aligns with process control concepts discussed in the patent literature.^57^ Among the investigated variables, the steam partial pressure *p*_H_2_O,effluent_ exerts the strongest influence on the molar product ratio *r*_CP_, clearly surpassing the effects of reactor inlet pressure *p*_in_ and molar steam-to-ethane feed ratio *r*_H_2_O/C_2_H_6__. Consequently, process optimization should prioritize controlling *p*_H_2_O,effluent_, as it offers the most direct and effective handle for tuning product distribution in ODHE.^57^

### 3.4 Effect of Coolant Temperature and WHSV

The effect of coolant temperature *T*_CM_ and WHSV is analyzed under the reported reaction conditions according to Table 2 (variation of WHSV).

In addition to its kinetic effect, WHSV also influences the thermal behavior of the reactor. Owing to the exothermic nature of the ODHE reactions, the average catalyst bed temperature *T*_bed_ exceeds the coolant temperature *T*_CM_ and attains a higher value under process-relevant, non-isothermal conditions.

For a given coolant temperature *T*_CM_, the average catalyst bed temperature *T*_bed_ remained essentially constant despite variations in the WHSV and the resulting changes in ethane conversion *X*_C_2_H_6__. This indicates that WHSV-induced changes in ethane conversion are compensated for by corresponding shifts in product selectivity within the investigated range.

Consequently, each coolant temperature *T*_CM_ can be associated with a corresponding average catalyst bed temperature *T*_bed_, as summarized in Table 4.

**Table 4:** Temperatures *T*_1_–*T*_7_ Correspond to Distinct Coolant Temperatures *T*_CM_ Varied in the Range of 275–305 °C, Each of Which Results in a Characteristic Average Catalyst Bed Temperature *T*_bed_

temperature	*T*_CM_	*T*_bed_	Δ*T*_max_	Δ*T*_bed_ ^a^
	°C	°C	K	K
*T*_1_	275	280	8	±2
*T*_2_	280	286	9	±3
*T*_3_	285	291	11	±3
*T*_4_	290	297	12	±5
*T*_5_	295	303	14	±5
*T*_6_	300	309	17	±5
*T*_7_	305	316	20	±7

a Process-relevant, non-isothermal conditions: Δ*T*_bed_ > ±2 K.

For convenience, these coupled operating conditions are represented by the discrete temperature labels *T*_1_–*T*_7_, which define specific experimental set points combining coolant temperature and the resulting average catalyst bed temperature.

The dependency of main product selectivities *S*_C_2_H_4__ and *S*_AcOH_ on coolant temperature *T*_CM_ and WHSV is shown in Figure 6.

**Figure 6:**
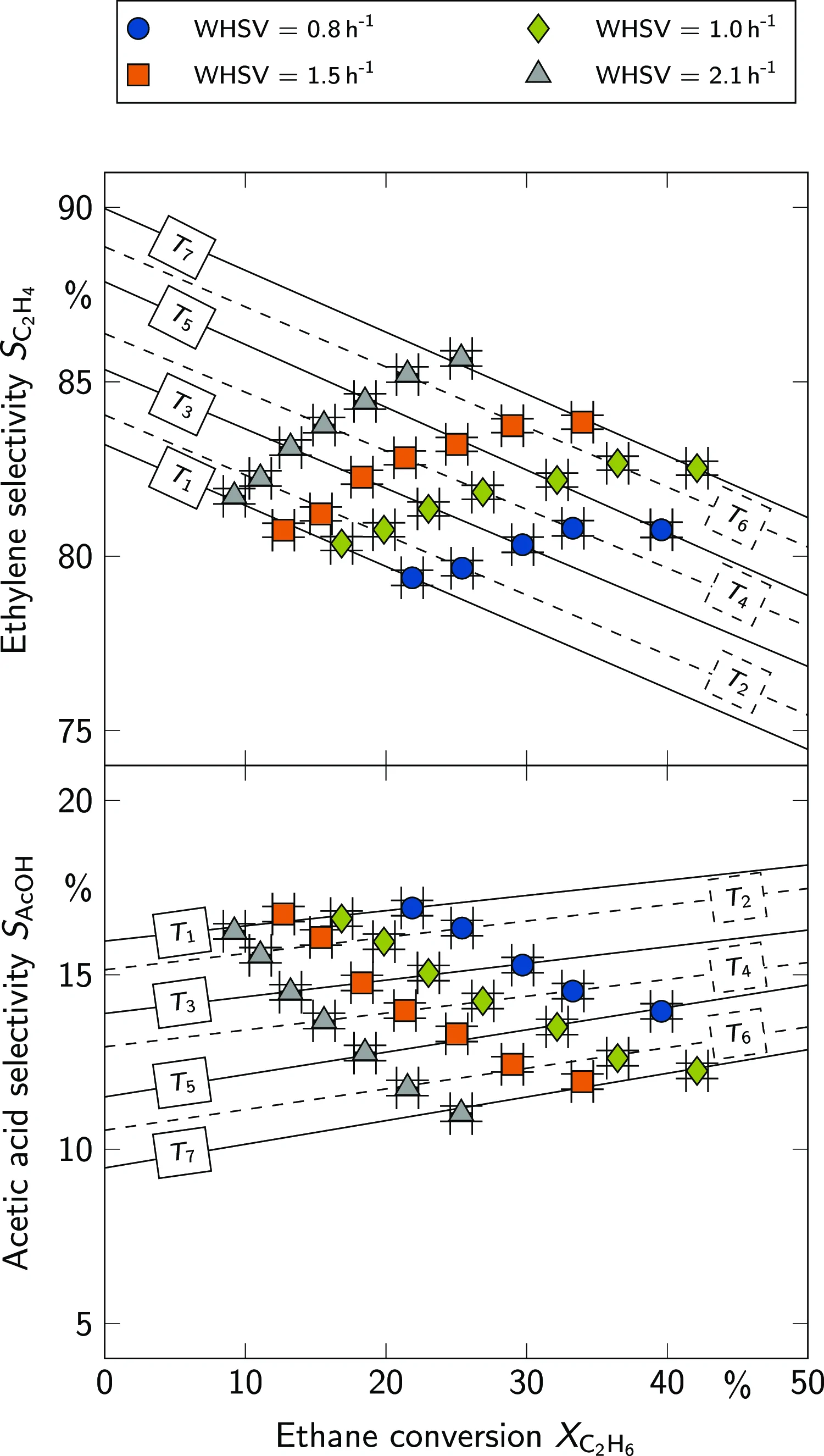
Dependency of *S*_C_2_H_4__ (top panel) and *S*_AcOH_ (bottom panel) on coolant temperature *T*_CM_ and WHSV at *p*_in_ = 3.5 bar, *r*_O_2_/C_2_H_6__ = 0.373, *r*_H_2_O/C_2_H_6__ = 0.427, *T*_CM_ = 275–305 °C with undiluted catalyst bed using Catalyst B.

Isotachs (lines of equal WHSV) are obtained by variation of coolant temperature *T*_CM_ at constant WHSV, and isotherms (lines of equal coolant temperature and average catalyst temperature *T*_*i*_ as reported in Table 4) are obtained by variation of WHSV at constant coolant temperature *T*_CM_ and average catalyst bed temperature *T*_bed_.

The main product selectivities *S*_C_2_H_4__ and *S*_AcOH_ depend strongly on *T*_CM_ and the WHSV. Along isotachs, increasing *T*_CM_ raises ethane conversion *X*_C_2_H_6__ and shifts the product distribution toward ethylene, while *S*_AcOH_ declines. Along isotherms, increasing the WHSV lowers *X*_C_2_H_6__ but yields a linear increase in *S*_C_2_H_4__ and a corresponding decrease in *S*_AcOH_.

Comparisons at equal ethane conversion *X*_C_2_H_6__ show that higher WHSV results in higher ethylene selectivity and lower acetic acid selectivity. This trend is attributed to the higher average catalyst bed temperatures *T*_bed_ needed for maintaining equal ethane conversion *X*_C_2_H_6__ at higher WHSV under non-isothermal operation. Higher average catalyst bed temperature may reduce the degree of coverage θ_H_2_O_ of the surface with water and thereby disfavor the water-assisted pathway to acetic acid. This behavior is consistent with coverage-based relationships in eqs 35 and 38, which link increasing degree of coverage θ_H_2_O_ of the surface with water to a higher reciprocal molar product ratio *r*_CP_^–1^ of acetic acid to ethylene. However, this effect is superimposed by changes in space time resulting from WHSV variation.

The carbon oxide selectivities *S*_CO_ and *S*_CO_2__ are dependent on *T*_CM_ and WHSV, as shown in Figure 7. Carbon monoxide selectivity *S*_CO_ and carbon dioxide selectivity *S*_CO_2__ increase linearly with ethane conversion *X*_C_2_H_6__ at constant WHSV, with carbon dioxide selecitivity remaining the lowest product selectivity compared to ethylene, acetic acid, and CO. The increase in both carbon oxides with rising conversion reflects the growing contribution of consecutive oxidation pathways with increasing ethane conversion. At constant coolant temperature *T*_CM_, increasing WHSV lowers the ethane conversion *X*_C_2_H_6__ and yields lower carbon oxide selectivities *S*_CO_ and *S*_CO_2__, resulting in linear isotherms.

**Figure 7:**
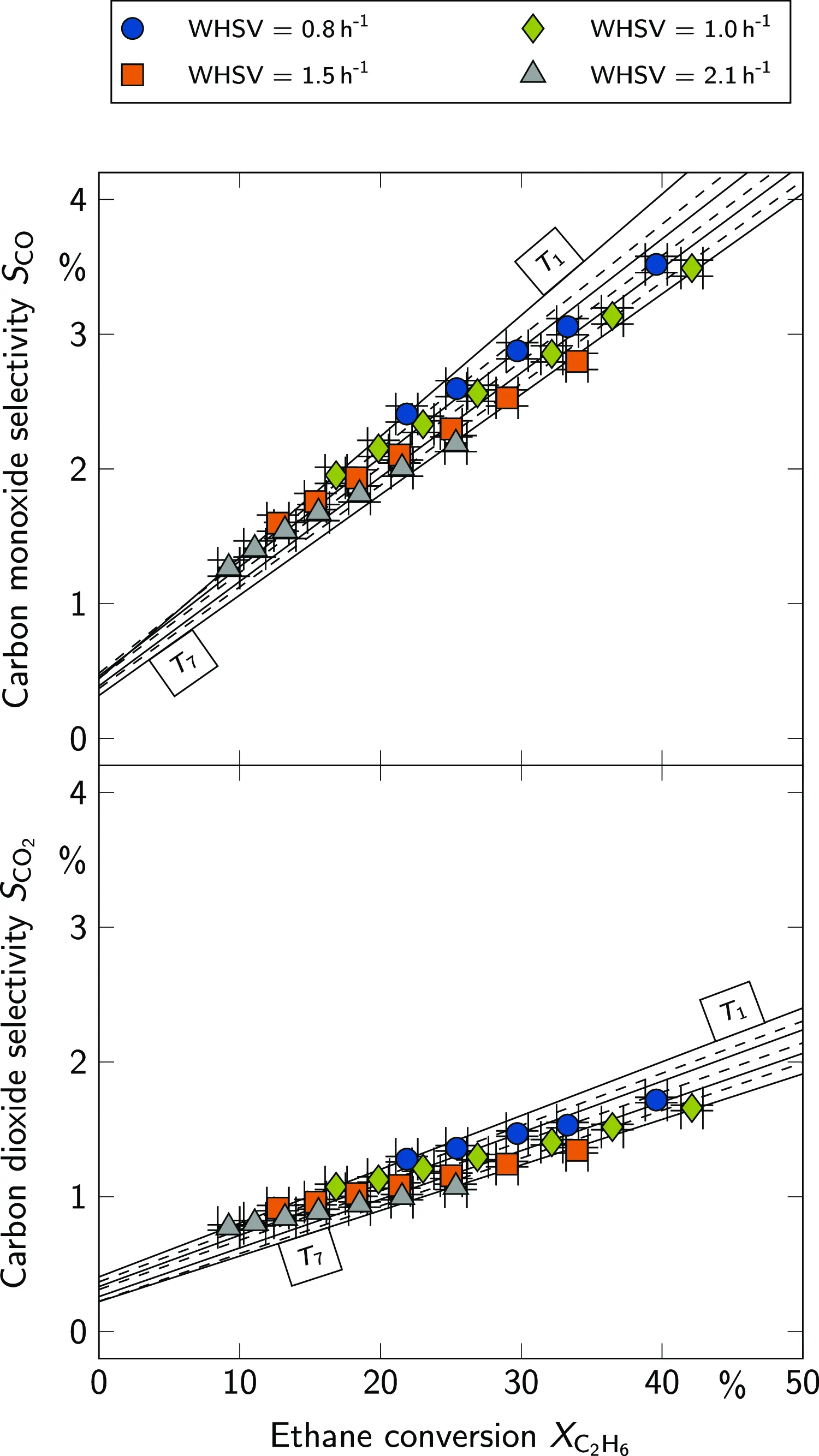
Dependency of *S*_CO_ (top panel) and *S*_CO_2__ (bottom panel) on coolant temperature *T*_CM_ and WHSV at *p*_in_ = 3.5 bar, *r*_O_2_/C_2_H_6__ = 0.373, *r*_H_2_O/C_2_H_6__ = 0.427, *T*_CM_ = 275–305 °C with undiluted catalyst bed using Catalyst B.

The dependency of molar product ratio *r*_CP_ of commercial products and the commercial product selectivity *S*_CP_ on coolant temperature and WHSV is shown in Figure 8.

**Figure 8:**
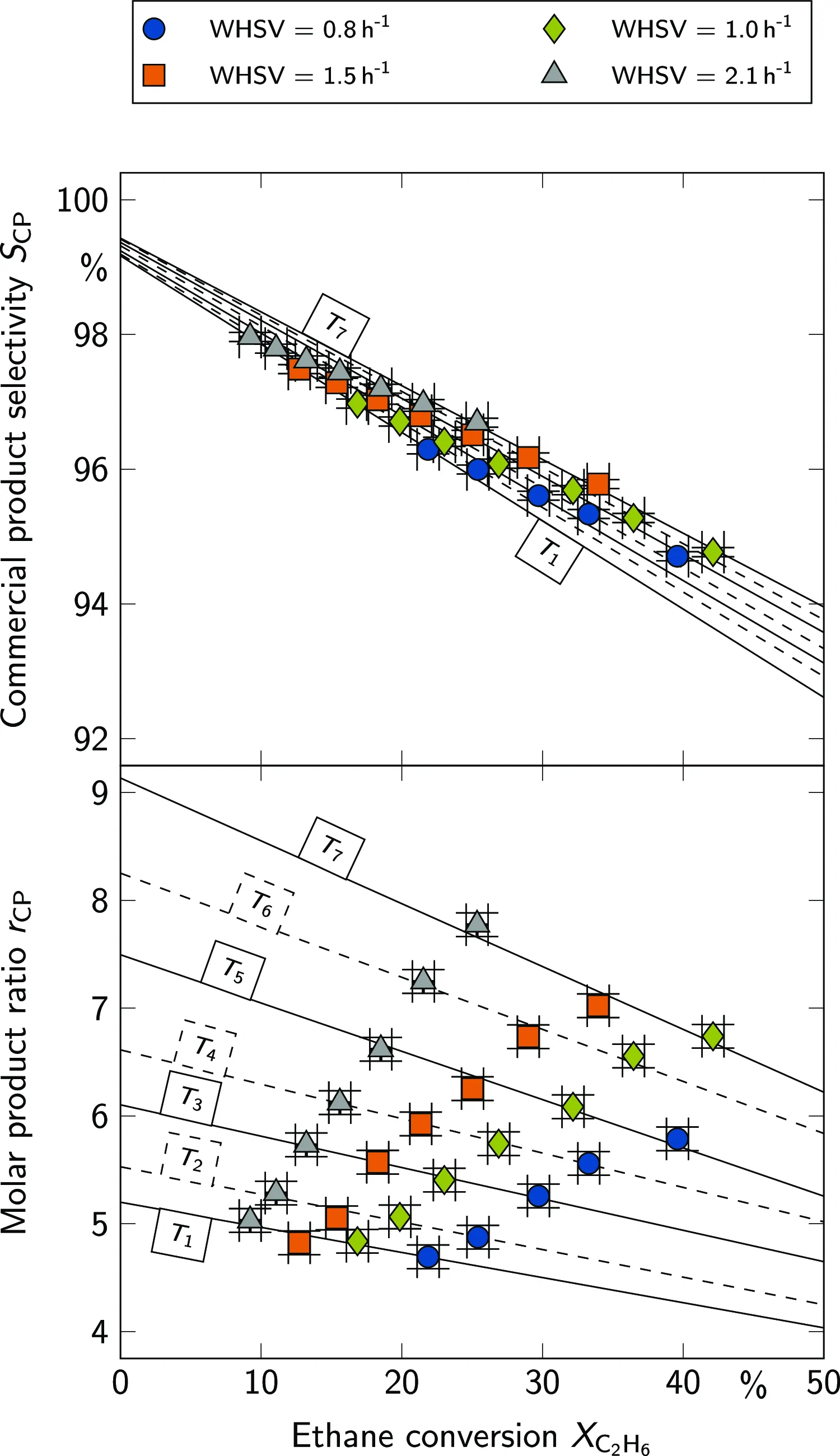
Dependency of *S*_CP_ (top panel) and *r*_CP_ (bottom panel) on coolant temperature *T*_CM_ and WHSV at *p*_in_ = 3.5 bar, *r*_O_2_/C_2_H_6__ = 0.373, *r*_H_2_O/C_2_H_6__ = 0.427, *T*_CM_ = 275–305 °C with undiluted catalyst bed using Catalyst B.

Since the individual selectivities of ethylene and acetic acid have been discussed in detail, the molar product ratio *r*_CP_ of commercial products and the commercial product selectivity *S*_CP_ can be interpreted analogously. They follow the same trends with respect to coolant temperature *T*_CM_ and WHSV, as they directly reflect the shift between ethylene formation and oxygenate formation governed by the same underlying dependencies and water-coverage effects. In addition to the coverage-based interpretation, differences in the apparent activation energies *E*_A,app,*i*_ of the overall formation of products *i* may also be used to explain the observed temperature-dependent selectivity trends. Apparent activation energies were determined from quasi-isothermal experiments (Table 2 – variation of Inert dil., 85 wt % inert dilution) according to the Arrhenius approach described in Section S1.3 (SI) and are shown in Table 5.

**Table 5:** Apparent Activation Energies *E*_A,app,*i*_ for the Formation of Main and Side Products *i* Determined from Quasi-Isothermal Experiments with Catalyst A

	unit	C_2_H_4_	AcOH	CO	CO_2_
*E*_A,app,*i*_	kJ mol^–1^	86	56	125	116

Since the determination is based on total molar product flow rates obtained during ethane oxidation, the resulting values represent apparent activation energies of overall product formation from ethane and do not distinguish between individual reaction pathways or elementary steps. Consequently, the apparent activation energies *E*_A,app,*i*_ reflect the aggregate temperature dependence of product formation and inherently include the influence of parallel and consecutive reaction pathways, adsorption and desorption equilibria, surface coverage effects, and transport-related phenomena under the investigated reaction conditions. A mechanistic discrimination between individual reaction pathways would require dedicated experiments addressing the respective intermediates, e.g., through separate ethylene and acetic acid oxidation studies.

The apparent activation energies *E*_A,app,*i*_ show a substantially higher value for ethylene formation (*E*_A,app,C_2_H_4__ = 86 kJ mol^–1^) than for acetic acid formation (*E*_A,app,AcOH_ = 56 kJ mol^–1^). Consequently, acetic acid formation is favored at lower temperatures, whereas increasing catalyst bed temperature results in a comparatively stronger increase in the ethylene formation rate. This behavior is consistent with the experimentally observed increase in ethylene selectivity and corresponding decrease in acetic acid selectivity at elevated temperatures.

The experimentally observed increase in ethylene selectivity and simultaneous decrease in acetic acid selectivity with increasing catalyst bed temperature are therefore consistent with both the apparent activation energies and the coverage-based interpretations. Since the apparent activation energies describe the overall formation of the respective products during ethane oxidation and inherently incorporate the influence of adsorption and desorption equilibria, surface coverage effects, and competing reaction pathways, they should not be interpreted independently of the coverage-based discussion. Rather, both approaches provide complementary descriptions of the observed temperature-dependent selectivity trends and consistently explain the shift from acetic acid toward ethylene formation at elevated temperatures.

Even higher values *E*_A,app,i_ > 100 kJ mol^–1^ for apparent activation energies for carbon monoxide and carbon dioxide formation are obtained. The corresponding increase in carbon oxide formation with increasing coolant temperature is reflected in selectivities *S*_CO_ and *S*_CO_2__ in Figure 7, but also in product yields *Y*_CO_ and *Y*_CO_2__, as shown in Section S2.4 (SI).

The determined values of apparent activation energy of formation of ethylene, acetic acid, and carbon oxides are further consistent with observations reported in the patent literature,^58^ where increasing reaction temperature favored ethylene formation relative to acetic acid formation.

The dependency of molar ratio *r*_CO_*x*__ of carbon oxides and total carbon oxide selectivity *S*_CO_*x*__ on coolant temperature *T*_CM_ and WHSV is shown in Section S2.4 (SI). The corresponding dependencies of the product yields *Y*_*i*_ are also provided in Section S2.4 (SI). The yield-based analysis confirms the trends previously identified in the selectivity discussion.

Extrapolation of the linear selectivity–conversion relationships toward vanishing ethane conversion indicates that ethylene, acetic acid, and carbon oxides exhibit nonzero intercepts. While ethylene clearly behaves as a primary product, the nonzero intercepts for acetic acid and carbon oxides suggest contributions from both primary and secondary formation pathways under the investigated conditions.

However, it should be noted that the intercept values for carbon oxides remain very small (below approximately 1%), indicating that their formation is still dominated by consecutive oxidation pathways. Thus, the apparent primary contributions of CO and CO_2_ formation are of minor quantitative relevance.

This observation slightly deviates from earlier interpretations, where especially acetic acid, but also carbon oxides, were predominantly regarded as secondary products,^1,29^ although alternative viewpoints exist.^27,28,30,39^ Additional experiments under pseudo-isothermal conditions supporting this interpretation are provided in Section 2.3 (SI).

From a process perspective, higher WHSV values are advantageous for maximizing ethylene selectivity while minimizing the total oxidation toward carbon oxides.

### 3.5 Trace Product Formation and Reaction Network

Besides main and side reaction products, methane and acetylene are formed in trace amounts during ODHE. Their formation was first analyzed for variation of coolant temperature *T*_CM_ and WHSV according to Table 2 (variation of WHSV). Figure 9 shows the dependence of trace product selectivities on ethane conversion *X*_C_2_H_6__.

**Figure 9:**
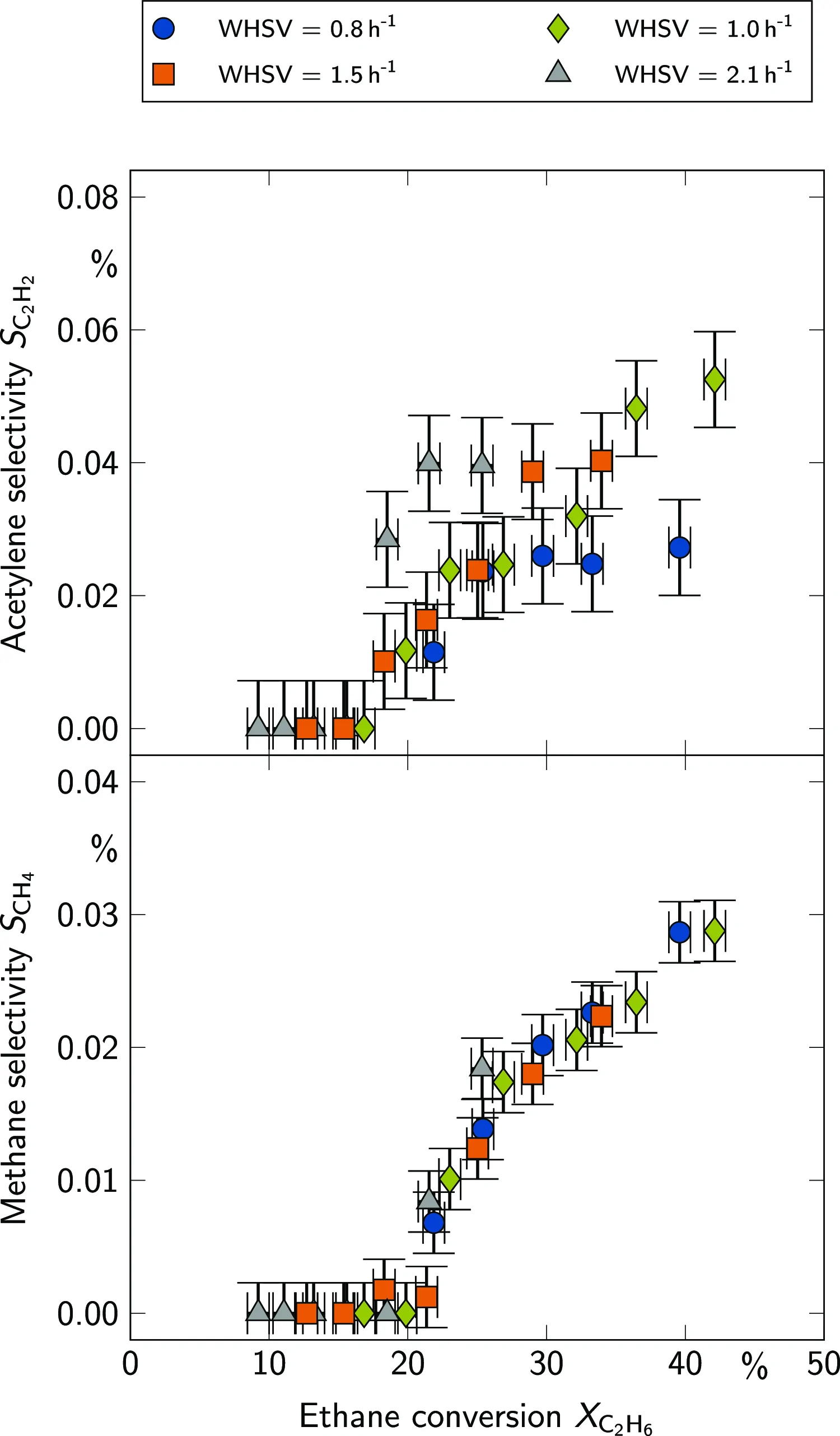
Dependency of *S*_CH_4__ (top panel) and *S*_C_2_H_2__ (bottom panel) on coolant temperature *T*_CM_ and WHSV at *p*_in_ = 3.5 bar, *r*_O_2_/C_2_H_6__ = 0.373, *r*_H_2_O/C_2_H_6__ = 0.427, *T*_CM_ = 275–305 °C with undiluted catalyst bed using Catalyst B.

Formation of methane and acetylene was observed starting at ethane conversion of *X*_C_2_H_6__ ≥ 20%, indicating that these species originate from secondary oxidation pathways that become significant at higher ethane conversion *X*_C_2_H_6__ or average catalyst bed temperature *T*_bed_.

The interpretation of trace product formation under non-isothermal conditions is complemented by a series of experiments conducted under pseudo-isothermal operation to elucidate the origin and impact of the trace products acetylene and methane. Under pseudo-isothermal conditions, the activity and selectivity of MoVNbTeO_*x*_ catalysts in ethane and ethylene oxidation are compared under reaction conditions according to Table 2 (C_2_H_6_ ox. and C_2_H_4_ ox.). The literature^20,22,59^ generally suggests that ethylene, because of its double bond and higher activation tendency, should exhibit higher reactivity. However, it is known in the literature that MoVNbTeO_*x*_ catalysts exhibit exceptionally low reactivity toward ethylene oxidation through tailored MoVNbTeO_*x*_ catalyst properties in the ODHE process.^26,30^

Table 6 summarizes the experimental results comparing ethane and ethylene oxidation.

**Table 6:** Comparison of Ethane and Ethylene Oxidation Using MoVNbTeO_*x*_ Catalysts at *p*_in_ = 3.5 bar, *r*_O_2_/C_2_H_n__ = 0.373, *r*_H_2_O/C_2_H_n__ = 0.427, Coolant Temperature *T*_CM_ = 310 °C, WHSV = 6.6 h^–1^ with 85 wt % Catalyst Bed Inert Dilution Using Catalyst A^a^

	unit	C_2_H_6_ oxidation		C_2_H_4_ oxidation	
*X*_C_2_H_n__	%	12.1	±0.2	1.2	±0.3
*S*_C_2_H_4__	%	87.5	±0.2		
*S*_AcOH_	%	10.3	±0.2	49.6	±0.7
*S*_CO_	%	1.3	±0.1	40.7	±0.4
*S*_CO_2__	%	0.9	±0.1	9.0	±0.5
*S*_CH_4__	%	BDL		0.2	±0.1
*S*_C_2_H_2__	%	BDL		0.8	±0.3

a Values below the detection limit (BDL) of the microGC are reported as BDL.

The conversion, i.e., activity for ethane oxidation, is approximately an order of magnitude higher than for ethylene oxidation, which is consistent with findings reported in the literature.^30^ Consequently, the substantial formation of acetic acid and carbon oxides observed at low ethane conversion cannot be attributed to the secondary oxidation of ethylene alone.

The formation of acetic acid and carbon oxides at low ethane conversion reflects that these products are formed within the initial reaction network under the investigated conditions, in line with the apparent primary behavior inferred from selectivity–conversion extrapolation. At higher ethane conversion, additional contributions from the oxidation of ethylene indicate the dual primary and secondary product character of acetic acid and carbon oxides.

The trace products methane and acetylene were detected in the non-isothermal experiments (variation of WHSV) only at higher ethane conversion *X*_C_2_H_6__ ≥ 20%. In contrast, under quasi-isothermal conditions, these species were observed exclusively during ethylene oxidation, while they were not detected during ethane oxidation.

This comparison indicates that the formation of methane and acetylene is linked to secondary pathways involving intermediate products, such as ethylene or acetic acid, rather than to primary ethane conversion. Acetylene presumably originates from the oxidative dehydrogenation of ethylene, whereas methane may be formed through oxidative cleavage of ethylene or acetic acid.

The occurrence of both trace products only at elevated ethane conversion under non-isothermal conditions further suggests that a minimum concentration or partial pressure of intermediate species such as ethylene is required for trace product formation.

Thus, ODHE over MoVNbTeO_*x*_ catalysts proceeds through a complex network of primary and secondary oxidation pathways. Table 1 lists the individual reactions considered in this work, while Figure 10 presents our proposed extended reaction network.^31^

**Figure 10:**
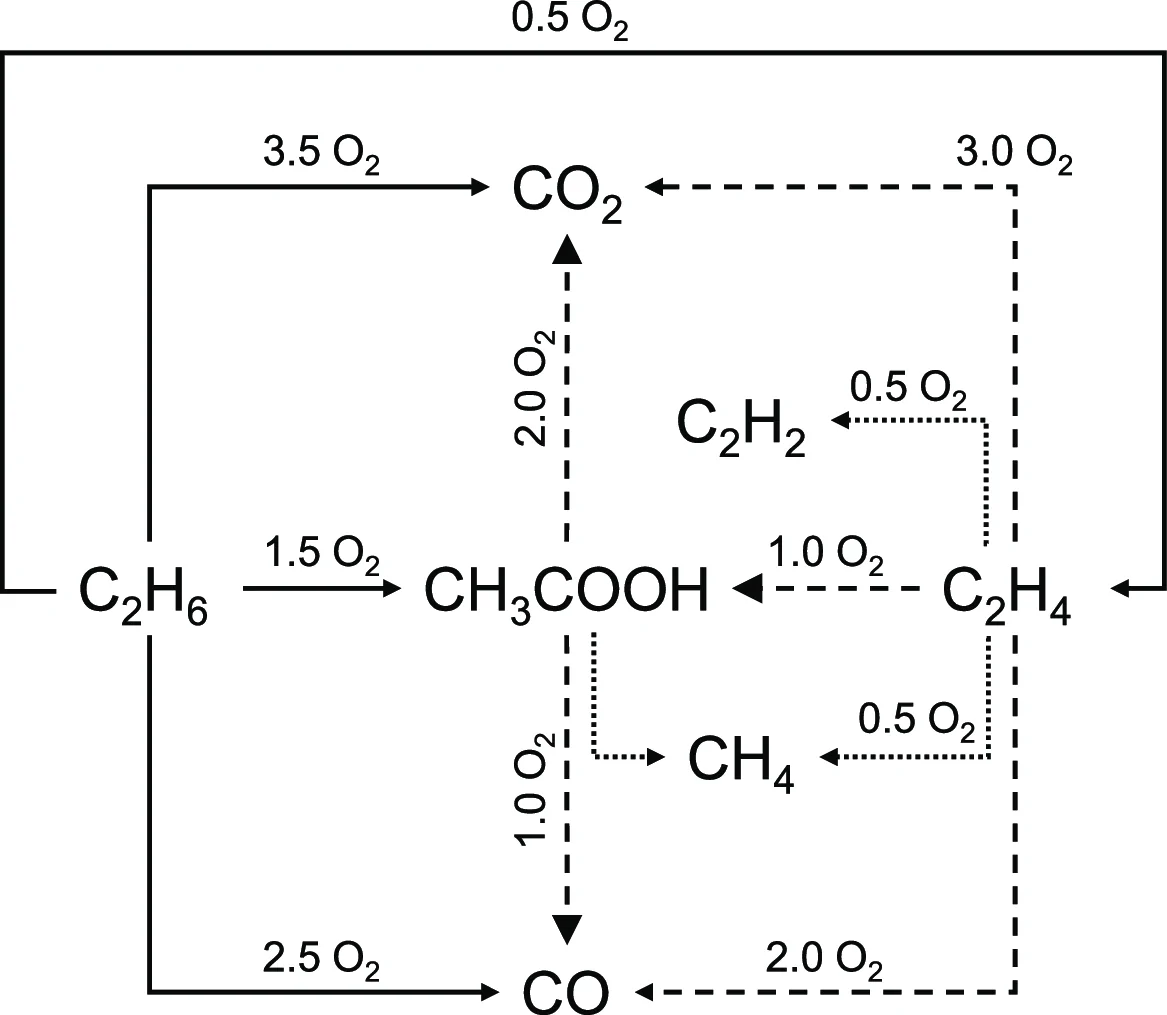
Reaction network considering the entire set of Reactions R2–R13, including primary reaction paths (solid), secondary reaction paths (dashed), and formation of trace products methane and acetylene (dotted).

In the reaction network, solid lines represent primary reactions of ethane, while dashed lines indicate the secondary oxidation of ethylene and acetic acid. Trace reaction pathways to methane and acetylene are included as dotted lines, representing an extension beyond existing reaction networks in the literature,^30,39^ where these trace reactions are not considered.

Although the selectivities of trace components methane and acetylene remain below 0.1%, awareness of their occurrence at elevated ethane conversion *X*_C_2_H_6__ under process-relevant conditions is important for process design, as both species affect product quality, product separation, and downstream applications including integration with steam cracking^60^ or use in vinyl acetate monomer production plants.^61^

Acetylene must be rigorously removed to prevent the deterioration of ethylene product quality and polymerization-induced fouling. In the EDHOX process, trace acetylene is removed in a gas cleanup reactor using a highly selective oxidation catalyst, reducing residual oxygen to molar ppm level and acetylene to molar ppb level without requiring hydrogenation.^38,62^ The oxygen concentration in the feed of the gas cleanup reactor should be minimized, as oxygen will subsequently oxidize carbon monoxide and ethylene to carbon dioxide and consequently increase direct CO_2_ emissions.^63^

Trace methane is separated in the cryogenic fractionation section together with carbon monoxide from unconverted ethane, enabling recovery and recycle.^47^ These observations highlight the importance of minimizing ethylene space time and controlling operating conditions to limit trace product formation while ensuring product purity and process reliability.

At higher ethane conversion *X*_C_2_H_6__, hydrogen was formed with its selectivity *S*_H_2__ linearly increasing with ethane conversion *X*_C_2_H_6__. Since the molar hydrogen concentration in the product gas never exceeded 25 ppm, and the origin of hydrogen formation could not be identified, hydrogen as a trace product is not further analyzed in this work.

## 4 Summary and Conclusion

Alternative pathways for ethylene production need to combine flexibility in production volumes with a significant reduction in CO_2_ emissions to complement or compete with conventional steam cracking. The oxidative dehydrogenation of ethane over MoVNbTeO_*x*_ catalysts has demonstrated promising potential to meet these requirements, offering high commercial product selectivity *S*_CP_ and flexible molar product ratio *r*_CP_ values of ethylene to acetic acid.

The correlations observed under kinetically oriented, pseudo-isothermal conditions are confirmed under process-relevant, non-isothermal reaction conditions reaching technically relevant ethane conversion with significant temperature increase over the catalyst bed. While pseudo-isothermal tests provide fundamental insights into intrinsic kinetics, experiments under process-relevant conditions are indispensable for identifying secondary pathways and trace product formation, which may only appear at elevated ethane conversion under process-relevant thermal regimes. The knowledge of trace product formation is crucial for a commercial-scale process design due to effects on product quality, product separation, and downstream applications.

Systematic variation of crucial operating parameters reveals distinct effects on ethane conversion *X*_C_2_H_6__ and the product distribution. Increasing the reactor inlet pressure *p*_in_ enhances ethane conversion *X*_C_2_H_6__ but shifts selectivity toward acetic acid and carbon oxides, reducing the molar product ratio *r*_CP_. An optimal steam-to-ethane ratio *r*_H_2_O/C_2_H_6__ prevents catalyst deactivation. Ethylene yield *Y*_C_2_H_4__ reaches a maximum at intermediate steam-to-ethane ratios, whereas the acetic acid yield *Y*_AcOH_ increases continuously over the investigated range. The steam partial pressure *p*_H_2_O,effluent_ in the wet product gas emerged as the dominant parameter governing the molar product ratio *r*_CP_, as it modulates the surface coverage of adsorbed water and thereby shifts the product distribution between ethylene and acetic acid formation.

Coolant temperature *T*_CM_ and WHSV influence ethane conversion *X*_C_2_H_6__ and product distribution mainly via their impact on the average catalyst bed temperature *T*_bed_ and the degree of coverage θ_H_2_O_ of the surface with water. Apparent activation energies determined from quasi-isothermal experiments indicate a substantially stronger temperature dependence of ethylene formation than of acetic acid formation, which additionally contributes to the observed shift in product distribution with increasing temperature.

Higher average catalyst bed temperature *T*_bed_ (e.g., at higher WHSV for a given ethane conversion *X*_C_2_H_6__) lowers the degree of coverage θ_H_2_O_ of the surface with water by promoting water desorption and thereby suppresses the water-assisted pathway to acetic acid, which increases the molar product ratio *r*_CP_ of ethylene to acetic acid. Consistently, the reciprocal molar product ratio *r*_CP_^–1^ scales linearly with the steam partial pressure *p*_H_2_O,effluent_ in the wet product gas, in accordance with a Langmuir-type coverage correlation.

Pseudo-isothermal comparison of ethane and ethylene oxidation confirms low reactivity of the ethylene oxidation through tailored MoVNbTeO_*x*_ catalyst properties in the ODHE process. The substantial formation of acetic acid and carbon oxides at low ethane conversion indicates that these products are not formed exclusively through the secondary oxidation of ethylene. This observation is consistent with the nonzero intercepts obtained from linear extrapolation of product selectivities *S*_*i*_ toward vanishing ethane conversion, although the apparent primary contribution of carbon oxides remains quantitatively small.

The trace products methane and acetylene were detected in the non-isothermal ODHE experiments at elevated ethane conversion, whereas under pseudo-isothermal conditions they were observed exclusively during ethylene oxidation and not during ethane oxidation. Although their selectivities remain below 0.1%, their presence is relevant for process design due to implications for product quality, separation, and downstream integration.

These results demonstrate that ODHE proceeds through a coupled network of versatile reaction pathways over MoVNbTeO_*x*_ catalysts and provide an approach to controlling product distribution under process-relevant conditions. Moreover, the strong sensitivity of the product distribution to operating parameters highlights the tunability of ODHE process technology.

By carefully adjusting the steam-to-ethane ratio, reactor inlet pressure, coolant temperature, and WHSV, the molar product ratio *r*_CP_ of ethylene to acetic acid and the corresponding product selectivities and product yields can be influenced. This tunability, combined with the potential to reduce CO_2_ emissions, positions ODHE over MoVNbTeO_*x*_ catalysts as a promising complementary route for ethylene and acetic acid production.

## Supplementary Material



## Notes

Detailed flowsheet and description of the laboratory ODHE setup (S1.1); exemplary calculation of conversion and selectivity (S1.2); apparent reaction rate constant and apparent activation energy determination (S1.3); assessment of measurement accuracy (S1.4); catalyst particle size selection and hydrodynamic considerations (S1.5); detailed experimental data for variation of catalyst bed inert dilution (S2.1); discussion of pressure influence via modified reciprocal GHSV and steam partial pressure (S2.2); primary and secondary product discussion based on pseudo-isothermal experiments (S2.3); and additional experimental data for variation of coolant temperature and WHSV (S2.4) (PDF)

## Nomenclature

Δ*H*_*R*_^0^: standard enthalpy of reaction [kJ mol^–1^]
Δ*M*: mass balance [−]
Δ*N*_*e*_: molar balance of element e [−]
Δ*T*_bed_: temperature deviation over cat. bed [K]
Δ*T*_max_: maximum temperature increase [K]
ν: stoichiometry factor [−]
θ: degree of coverage [−]
φ_*i*_: volume fraction [−]
σ: standard deviation [−]
*A*: apparent pre-exponential factor [mol bar^–1^ kg^–1^ h^–1^]
*d*_p_: catalyst particle diameter [mm]
*d*_p,max_: maximum catalyst particle diameter [mm]
*d*_p,min_: minimum catalyst particle diameter [mm]
*D*_R_: reactor inner diameter [mm]
*E*_A,app,*i*_: apparent activation energy for formation of product *i* [kJ mol^–1^]
GHSV_mod_: mod. gas hourly space velocity [L_gas_ s^–1^ kg_act_^–1^]
*k*: apparent reaction rate constant [mol bar^–1^ kg^–1^ h^–1^]
*K*: Langmuir adsorption constant [bar^–1^]
*L*_bed_: catalyst bed length [cm]
*M*_*i*_: mass of component *i* [g]
*M*_cat,act_: catalyst active mass [kg]
*Ṁ*_*i*_: mass flow rate of component *i* [g h^–1^]
*M̅*_*i*_: molar mass of component *i* [g mol^–1^]
*n*: refractive index [−]
*Ṅ*_*i*_: molar flow rate of component *i* [mol h^–1^]
*p*_*i*_: partial pressure of component *i* [bar]
*p*_in_: reactor inlet pressure [bar]
*p*_out_: reactor outlet pressure [bar]
*R*: universal gas constant [J mol^–1^ K^–1^]
*r*_*i*/*j*_: molar ratio of components *i* and *j* [−]
*Re*_p_: particle Reynolds number [−]
*S*_*i*_: selectivity of product *i* [−]
*T*_bed_: average catalyst bed temperature [°C]
*T*_bed,min_: lowest catalyst bed temperature [°C]
*T*_CM_: coolant temperature [°C]
*T*_HS_: hotspot temperature [°C]
*V̇*_feed_: volumetric feed flow rate [L_gas_ s^–1^]
*w*_*i*_: mass fraction of component *i* [−]
WHSV: weight hourly space velocity [g_feed_ g_cat_^–1^ h^–1^]
*x*_*i*_: molar fraction of component *i* [−]
*X*_*i*_: conversion of reactant *i* [−]
*Y*_*i*_: yield of product *i* [−]
**Abbreviations**
BDL: below detection limit
CP: commercial product
CM: coolant medium
Cond: condensate
DPG: dry product gas
GC: gas chromatography
HPLC: high-performance liquid chromatography
HS: hotspot
MFC: mass flow controller
ODHE: oxidative dehydrogenation of ethane
WPG: wet product gas
