# Supplementary material for: Impact of Reaction Conditions on Product Distribution in Oxidative Dehydrogenation of Ethane over MoVNbTeO x Catalysts
Source: Ind Eng Chem Res. 2026 Oct 1;65(40):21525–41. doi: 10.1021/acs.iecr.6c02345 (PMC13679875; doi:10.1021/acs.iecr.6c02345)
Supplement: Supplementary file 1 [file ie6c02345_si_001.pdf]

Supporting Information: Impact of Reaction Conditions on Product Distribution in Oxidative Dehydrogenation of Ethane over $MoVNbTeO_x$ Catalysts

Yannick Weis*[1], Martin Schubert[2], Andreas Meiswinkel[2], Sebastian Böcklein[3], Klaus Wanninger[3], Gerhard Mestl[3], Sebastian Rehfeldt[1], and Harald Klein[1]

[1]TUM School of Engineering and Design, Department of Energy and Process Engineering, Institute of Plant and Process Technology, Technical University of Munich, Garching 85748, Germany
[2]Linde GmbH, Linde Engineering, Pullach 82049, Germany
[3]Clariant Produkte (Deutschland) GmbH, Heufeld 83052, Germany

*Email: yannick.weis@tum.de

The following topics are adressed in the Supporting Information:

- S1.1 Detailed flowsheet and description of the laboratory ODHE setup
- S1.2 Exemplary calculation of conversion and selectivity
- S1.3 Apparent reaction rate constant and apparent activation energy determination
- S1.4 Assessment of measurement accuracy
- S1.5 Catalyst particle size selection and hydrodynamic considerations
- S2.1 Detailed experimental data for variation of catalyst bed inert dilution
- S2.2 Discussion of pressure influence via modified reciprocal GHSV and steam partial pressure
- S2.3 Primary and secondary product discussion based on pseudo-isothermal experiments
- S2.4 Additional experimental data for variation of coolant temperature and WHSV

S1 Additional Experimental Details

S1.1 Detailed Flowsheet and Description of the Laboratory ODHE Setup

Figure S1 shows a simplified block flow diagram of the experimental setup.

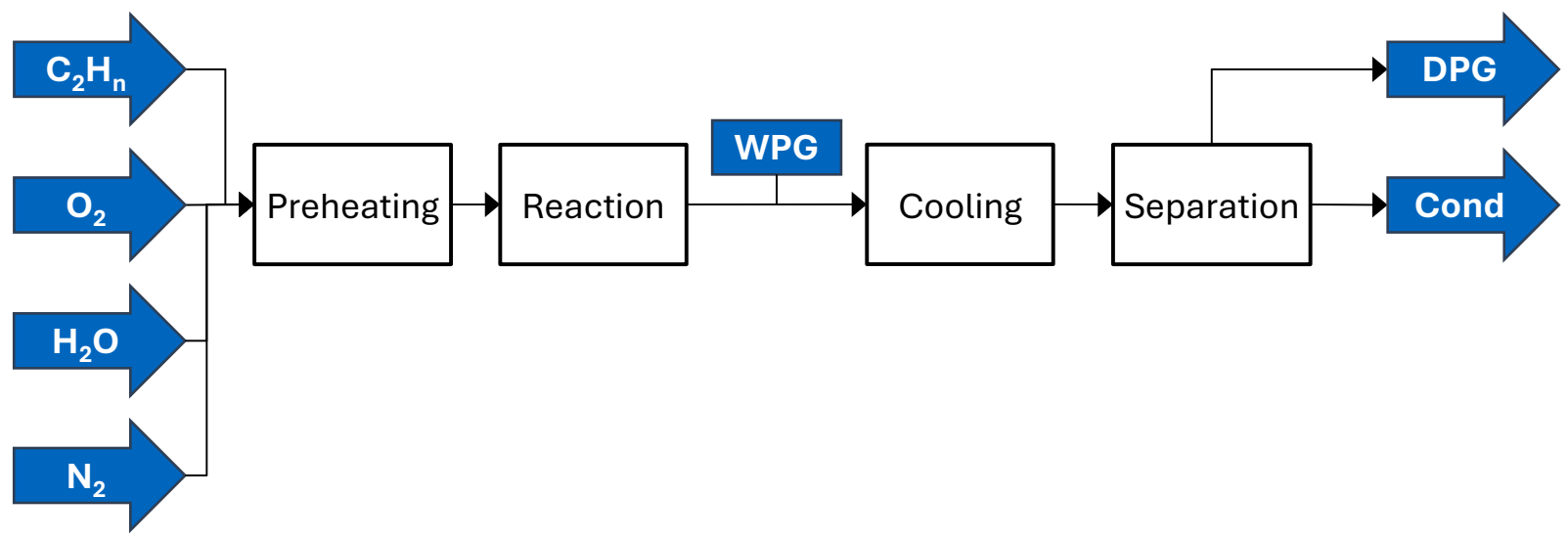

Figure S1: Block flow diagram of laboratory-scale ODHE unit comprising feed supply, tubular reactor with wet product gas (WGP) as effluent and separation unit yielding dry product gas (DPG) and condensate (Cond).

Figure S2 shows a detailed flowsheet of the laboratory plant including feed supply, tubular reactor, separation part and important measuring and control technology.

The reactants ethane and oxygen and inert nitrogen are supplied from gas cylinders via mass flow controllers (MFC, Bronkhorst, E Flow). Ethane is preheated by electrical heat tracing EHT 01. Demineralized water is supplied from a water storage tank C 01 by a HPLC pump P 01 (Fink, Carino 09). The water feed mass flow rate is monitored gravimetrically by placing the water tank on a scale during operation. Before entering the tubular reactor, the reactant mixture of water, oxygen, ethane and optionally nitrogen is vaporized in a tubular furnace preheater H 01 (HTM Reez, LOSA). Electrical heat tracing EHT 02 and insulation with mineral wool of the subsequent feed line prevent recondensation of the preheated gas mixture before entering the tubular reactor R 01. A continuous measurement of the reactor inlet pressure p_{in} is provided by PIR 02 on the ethane feed line.

The reactor is a stainless steel (SS321) double tube with a length of 900 mm. The inner tube containing the catalyst bed has an inner diameter of 10 mm and a wall thickness of 2 mm. The outer tube has an inner diameter of 25 mm and a wall thickness of 2 mm. Thermal oil (Lauda, Ultra350) flows co-currently through the outer tube of the reactor, which surrounds the actual reaction tube, to heat the reactor and further to remove the heat generated by the exothermic main and side reactions. The thermal oil is pumped and temperature controlled by a thermooil heater (Lauda, Integral IN 8 XTW).

After the reactor, the product gas is cooled in a tubular heat exchanger E 01 condensing a water and acetic acid mixture. Cryostat E 02 (Lauda, Alpha) provides a coolant mixture consisting of ethylene glycol and water. In separator S 01, the two-phased product stream is separated into a liquid and gaseous fraction. The liquid fraction (condensate) is collected in condensate tank C 02. Condensate tanks are weighed to determine the condensate mass flow rate M_{cond}. A level sensor and switch LS 01 is applied for automatic condensate discharge. The mass flow rate $\dot{M}_{\mathrm{DPG}}$ of the gaseous fraction (dry product gas) is detected by a Coriolis flow meter FIR 06. The pressure control valve PICR 04 in the main product gas line enables manual control of the reactor inlet pressure p_{in}.

The dry product gas composition after condensation of water and acetic acid is on-line analyzed in a two channel gas chromatograph (Micro GC Fusion, Inficon GmbH) consisting of two modules using a molecular sieve column (module A) with argon as a carrier gas and a Rt-U-BOND column (module B) with helium as carrier gas. With increasing retention time, module A separates H_2, O_2, N_2, CH_4 and CO, while module B separates the carbon compounds CO_2, C_2H_4, C_2H_6 and C_2H_2. The modules yield the product composition as volume fractions φ_i derived from the TCD detector response relating the thermal-conductivity-based signal to the corresponding concentration and are evaluated using EZIQ software. The dry product gas composition was monitored by gas chromatography at regular intervals of five minutes. The microGC was calibrated using three calibration gases with the compositions given in Table S1.

Several temperature sensors TIR(S) 04 – 11 enable the measurement of a temperature profile over the

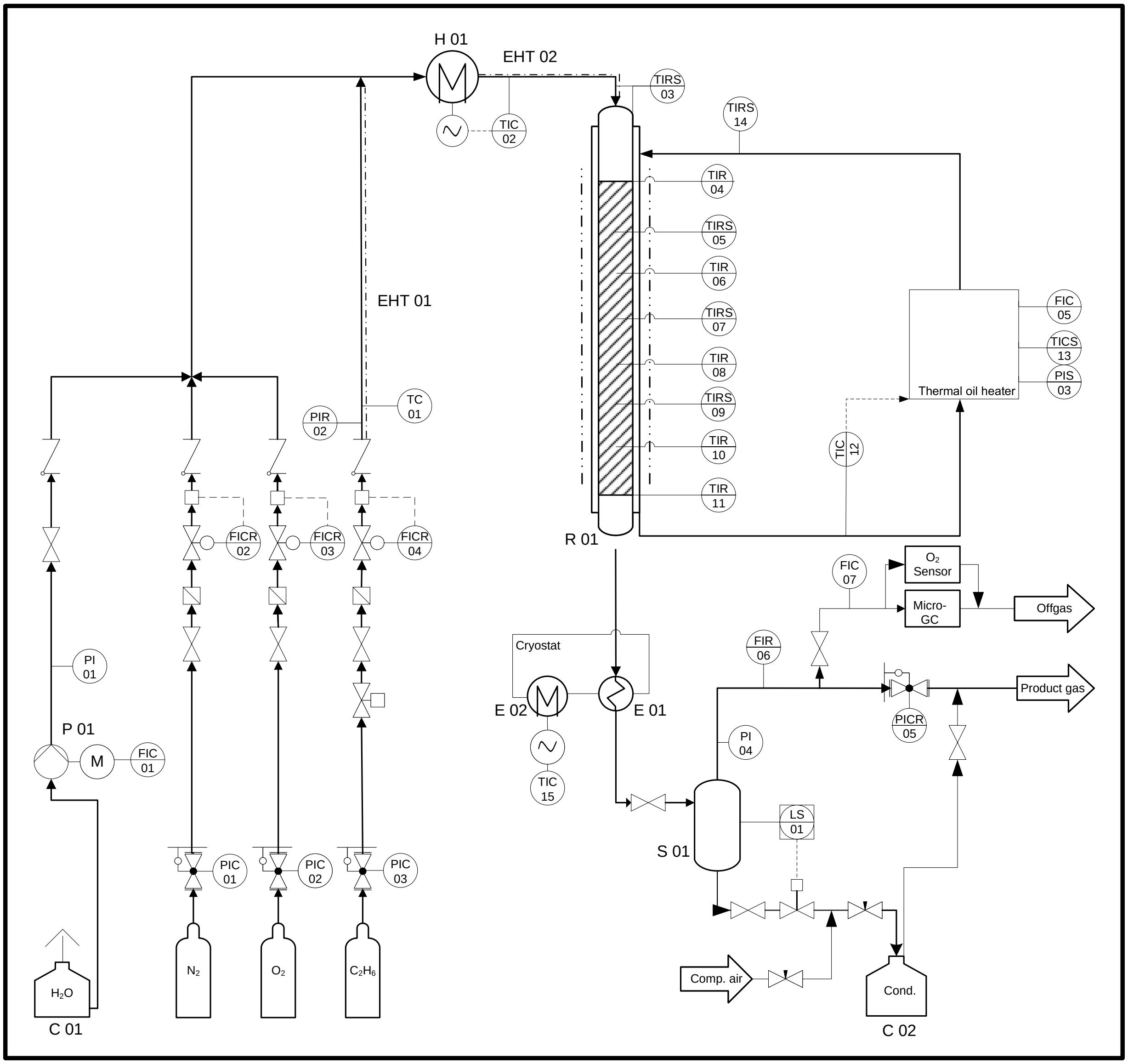

Figure S2: Flowsheet of the laboratory plant for investigation of ODHE including feed supply, tubular reactor and separation unit. Important process measuring and control technology is indicated.

Table S1: Calibration gas compositions for calibration of the gas chromatograph.

Molar fraction x_i		Gas 1	Gas 2	Gas 3
x_{H_2}	mol-%	1.0	0.5	-
x_{O_2}	mol-%	-	0.5	5.0
x_{N_2}	mol-%	7.0	15.0	95.0
x_{CH_4}	mol-%	0.05	1.0	-
x_{CO}	mol-%	4.0	6.0	-
x_{CO_2}	mol-%	1.5	4.0	-
$x_{C_2H_4}$	mol-%	43.0	35.0	-
$x_{C_2H_6}$	mol-%	43.4	38.0	-
$x_{C_2H_2}$	mol-%	0.05	-	-

inert and catalyst filling. Six temperature sensors TIR(S) 05 – 10 are distributed uniformly over the catalyst bed and are introduced centrally into the reactor as a bundled assembly with different sensor lengths from the bottom to indicate the maximum temperature increase $\Delta T_{\max}$ due to hotspot formation. In case of a thermal runaway the switches TIRS 05 - 9 lead to plant shutdown. The pressure drop over the catalyst bed can be obtained from the reactor inlet (PIR 02) and outlet (PI 04) pressure.

For each experiment, the laboratory plant was operated at steady-state conditions for a continuous two-hour period with all process variables continuously logged at a sampling rate of $0.5\,\mathrm{s}^{-1}$ and the representative steady-state values were obtained as arithmetic mean over the two-hour period.

S1.2 Exemplary Calcuation of Conversion and Selectivity

The ethane conversion $X_{\mathrm{C_2H_6}}$ in ethane oxidation can be calculated according to

$$X_{\mathrm{C_2H_6}} = \frac{\sum_{i=0}^{m}(\dot{N}_i \cdot \nu_{\mathrm{c},i}) - \dot{N}_{\mathrm{C_2H_6}} \cdot \nu_{\mathrm{c,C_2H_6}}}{\sum_{i=0}^{m}(\dot{N}_i \cdot \nu_{\mathrm{c},i})} = \frac{Z_1 - \dot{N}_{\mathrm{C_2H_6}} \cdot \nu_{\mathrm{c,C_2H_6}}}{Z_1} \tag{S1}$$

with

$$\begin{aligned} Z_1 = \sum_{i=0}^{m}(\dot{N}_i \cdot \nu_{\mathrm{c},i}) = {} & \dot{N}_{\mathrm{C_2H_6}} \cdot \nu_{\mathrm{c,C_2H_6}} + \dot{N}_{\mathrm{C_2H_4}} \cdot \nu_{\mathrm{c,C_2H_4}} + \dot{N}_{\mathrm{AcOH}} \cdot \nu_{\mathrm{c,AcOH}} + \dot{N}_{\mathrm{CO}} \cdot \nu_{\mathrm{c,CO}} \\ & + \dot{N}_{\mathrm{CO_2}} \cdot \nu_{\mathrm{c,CO_2}} + \dot{N}_{\mathrm{CH_4}} \cdot \nu_{\mathrm{c,CH_4}} + \dot{N}_{\mathrm{C_2H_2}} \cdot \nu_{\mathrm{c,C_2H_2}} \,. \end{aligned} \tag{S2}$$

The ethylene conversion $X_{\mathrm{C_2H_4}}$ in ethylene oxidation can be calculated according to

$$X_{\mathrm{C_2H_4}} = \frac{\sum_{i=0}^{m}(\dot{N}_i \cdot \nu_{\mathrm{c},i}) - \dot{N}_{\mathrm{C_2H_4}} \cdot \nu_{\mathrm{c,C_2H_4}}}{\sum_{i=0}^{m}(\dot{N}_i \cdot \nu_{\mathrm{c},i})} = \frac{Z_2 - \dot{N}_{\mathrm{C_2H_4}} \cdot \nu_{\mathrm{c,C_2H_4}}}{Z_2} \tag{S3}$$

with

$$\begin{aligned} Z_2 = \sum_{i=0}^{m}(\dot{N}_i \cdot \nu_{\mathrm{c},i}) = {} & \dot{N}_{\mathrm{C_2H_4}} \cdot \nu_{\mathrm{c,C_2H_4}} + \dot{N}_{\mathrm{AcOH}} \cdot \nu_{\mathrm{c,AcOH}} + \dot{N}_{\mathrm{CO}} \cdot \nu_{\mathrm{c,CO}} + \dot{N}_{\mathrm{CO_2}} \cdot \nu_{\mathrm{c,CO_2}} \\ & + \dot{N}_{\mathrm{CH_4}} \cdot \nu_{\mathrm{c,CH_4}} + \dot{N}_{\mathrm{C_2H_2}} \cdot \nu_{\mathrm{c,C_2H_2}} \,. \end{aligned} \tag{S4}$$

The acetic acid selectivity S_{AcOH} in ethane oxidation can be calculated according to

$$S_{\mathrm{AcOH}} = \frac{\dot{N}_{\mathrm{AcOH}} \cdot \nu_{\mathrm{c,AcOH}}}{Z_1 - \dot{N}_{\mathrm{C_2H_6}} \cdot \nu_{\mathrm{c,C_2H_6}}} \,. \tag{S5}$$

The acetic acid selectivity S_{AcOH} in ethylene oxidation can be calculated according to

$$S_{\mathrm{AcOH}} = \frac{\dot{N}_{\mathrm{AcOH}} \cdot \nu_{\mathrm{c,AcOH}}}{Z_2 - \dot{N}_{\mathrm{C_2H_4}} \cdot \nu_{\mathrm{c,C_2H_4}}} \,. \tag{S6}$$

The individual product selectivites S_i for C_2H_4, CO, CO_2, CH_4 and C_2H_2 can be calculated analogously.

S1.3 Apparent Reaction Rate Constant and Apparent Activation Energy Determination

The reaction rate as a function of apparent reaction rate constant k, ethane molar fraction $x_{\mathrm{C_2H_6}}$ and oxygen molar fraction $x_{\mathrm{O_2}}$ in the feed and their corresponding reaction orders x and y can be calculated according to

$$r = k \cdot {x_{\mathrm{C_2H_6}}}^{x} \cdot {x_{\mathrm{O_2}}}^{y} \,. \tag{S7}$$

Assuming a first-order reaction regarding ethane and zero-order reaction regarding oxygen due to high oxygen extent and low ethane conversion yiels the simplified reaction rate equation

$$r = k \cdot x_{\mathrm{C_2H_6}} \,. \tag{S8}$$

Using the definition of the reaction rate r as the time dependent change of the ethane molar flow rate $\dot{N}_{\mathrm{C_2H_6}}$ related to the catalyst mass M_{cat} and transforming the ethane molar fraction by Dalton's law yields

$$\frac{d\dot{N}_{\mathrm{C_2H_6}}}{dM_{\mathrm{cat}}} = k \cdot p_{\mathrm{in}} \cdot \frac{\dot{N}_{\mathrm{C_2H_6}}}{\dot{N}_{\mathrm{feed}}} \,. \tag{S9}$$

The molar flow rate $\dot{N}_{\mathrm{C_2H_6}}$ of ethane can be correlated to the ethane conversion $X_{\mathrm{C_2H_6}}$. Integrating the function over its variables results in

$$-\ln(1 - X_{\mathrm{C_2H_6}}) = k \cdot p_{\mathrm{in}} \cdot \frac{M_{\mathrm{cat}}}{\dot{N}_{\mathrm{feed}}} \,. \tag{S10}$$

Applying the definition of a modified weight hourly space velocity $\mathrm{WHSV_{mod}}$ as ratio of feed molar flow rate $\dot{N}_{\mathrm{feed}}$ and catalyst mass M_{cat} enables the determination of the apparent reaction rate constant k as a measure of the overall catalyst activity according to

$$-\ln(1 - X_{\mathrm{C_2H_6}}) = k \cdot p_{\mathrm{in}} \cdot \frac{1}{\mathrm{WHSV_{mod}}} \,. \tag{S11}$$

The overall ethane conversion can be described according to

$$X_{\mathrm{C_2H_6}} = 1 - \exp\left(-k' \frac{1}{\mathrm{WHSV}}\right) , \tag{S12}$$

where k' denotes an apparent activity parameter obtained from the ethane conversion data. The corresponding molar flow rate $\dot{N}_{\mathrm{C_2H_6,conv}}$ of converted ethane was then calculated as

$$\dot{N}_{\mathrm{C_2H_6,conv}} = \dot{N}_{\mathrm{C_2H_6,feed}} \left(1 - \exp\left(-k' \frac{1}{\mathrm{WHSV}}\right)\right) \,. \tag{S13}$$

For each product i, the measured product molar flow rate $\dot{N}_i$ was plotted as a function of the converted ethane molar flow rate $\dot{N}_{\mathrm{C_2H_6,conv}}$. Linear regression yielded a product-specific apparent rate constant k'_i according to

$$\dot{N}_i = k'_i \, \dot{N}_{\mathrm{C_2H_6,conv}} \,. \tag{S14}$$

To account for the pressure dependency introduced by the applied formulation, the apparent rate constants k'_i were normalized by the reactor inlet pressure p_{in}:

$$k_i = \frac{k'_i}{p_{\mathrm{in}}} \,. \tag{S15}$$

The resulting apparent product-specific reaction rate constants k_i for products i were used for Arrhenius analysis. Assuming Arrhenius-type behavior, the temperature dependency can be described by

$$k_i = A_i \exp\left(-\frac{E_{\mathrm{A,app},i}}{RT_{\mathrm{bed}}}\right) , \tag{S16}$$

with A_i as apparent pre-exponential factor, $E_{\mathrm{A,app},i}$ as apparent activation energy of formation of product i, and R as universal gas constant.

The logarithmic form

$$\ln(k_i) = \ln(A_i) - \frac{E_{\mathrm{A,app},i}}{R} \frac{1}{T_{\mathrm{bed}}} \,, \tag{S17}$$

allows determination of the apparent activation energy $E_{\mathrm{A,app},i}$ from the slope of the Arrhenius plot.

It should be noted that the resulting apparent activation energies $E_{\mathrm{A,app},i}$ represent overall formation characteristics of the respective products and do not correspond to activation energies of individual elementary reaction steps. The determination is based on the total molar product flow rate $\dot{N}_i$ and therefore reflects the combined contribution of all reaction pathways leading to product i. Consequently, the obtained values represent apparent activation energies of overall product formation, incorporating potential contributions from parallel and consecutive reaction pathways, adsorption and desorption phenomena, as well as transport effects.

S1.4 Assessment of Measurement Accuracy

Measurement uncertainty was estimated based on repeated analysis of a standard operating point under typical process conditions of coolant temperature $T_{\mathrm{CM}} = 295\,^{\circ}\mathrm{C}$, reactor inlet pressure $p_{\mathrm{in}} = 3.5\,\mathrm{bar}$, $r_{\mathrm{O_2/C_2H_6}} = 0.373$, $r_{\mathrm{H_2O/C_2H_6}} = 0.427$, and $\mathrm{WHSV} = 1.5\,\mathrm{h^{-1}}$ with non-diluted catalyst bed using Catalyst B. This point was measured 15 times, and the standard deviation σ of key performance indicators ethane conversion $X_{\mathrm{C_2H_6}}$, product selectivities S_{i} and reaction conditions was calculated. Error bars are included in all figures for all data points and represent the determined value $\pm 2\sigma$.

S1.5 Catalyst Particle Size Selection and Hydrodynamic Considerations

The catalyst particle size fraction of 1.0 mm to 2.5 mm was selected based on hydrodynamic design considerations while maintaining an acceptable pressure drop under the investigated operating conditions. Although smaller particle sizes generally improve radial mixing and approach ideal plug flow behavior, they also lead to substantially increased pressure drop for the investigated catalyst bed length of approximately 54 cm and total reactor filling height of 90 cm. We accepted pressure drops below 0.3 − 0.5 bar due to pressure sensitivity of the ODHE reactions and overall low reaction pressure regime.

To assess the hydrodynamic characteristics of the reactor, the reactor-to-particle diameter ratio, the bed length-to-particle diameter ratio, and the particle Reynolds number were evaluated under representative operating conditions.

The feed gas consisted of 56 % ethane, 20 % oxygen, and 24 % steam at a coolant temepravure of 300 °C and a reactor inlet pressure of 3.5 bar. Dynamic viscosities μ_i and densities ρ_i of the pure components were obtained from the NIST database [1] and are shown in Table S2.

Table S2: Pure-component properties used for estimation of mixture properties at 300 °C and 3.5 bar. Density and viscosity data of ethane, oxygen, and water were obtained from the NIST Thermophysical Properties of Fluid Systems database [1].

	μ_i Pa s	ρ_i $\mathrm{kg\,m^{-3}}$
C_2H_6	1.67×10^{-5}	2.21
O_2	3.37×10^{-5}	2.35
H_2O	2.03×10^{-5}	1.33

Mixture properties were estimated as mole-fraction-weighted averages according to

$$\mu_{\text{mix}} = \sum_i x_i \mu_i \qquad \text{and} \qquad \rho_{\text{mix}} = \sum_i x_i \rho_i \,, \tag{S18}$$

with x_i as the molar fraction of component i.
Using the pure-component properties listed in Table S2, the resulting mixture properties are

$$\mu_{\text{mix}} = 2.10 \times 10^{-5}\,\text{Pa}\,\text{s} \qquad \text{and} \qquad \rho_{\text{mix}} = 2.03\,\text{kg}\,\text{m}^{-3}\,. \tag{S19}$$

For the largest catalyst particles ($d_{\text{p,max}} = 2.5\,\text{mm}$), the bed length-to-particle diameter ratio is

$$\frac{L_{\text{bed}}}{d_{\text{p,max}}} = \frac{540\,\text{mm}}{2.5\,\text{mm}} = 216 \qquad \text{and} \qquad \frac{L_{\text{bed}}}{d_{\text{p,max}}} > 50\,, \tag{S20}$$

indicating that the relative influence of bed entrance and bed end effects are expected to be negligible [2].
The particle Reynolds number Re_p was calculated according to

$$\text{Re}_\text{p} = \frac{\rho_{\text{mix}}\, u\, d_\text{p}}{\mu_{\text{mix}}}\,, \tag{S21}$$

with the superficial gas velocity within the void fraction of the bed calculated using the volumentric feed flow rate $\dot{V}_{\text{feed}}$, the reactor cross-sectional area A_R and a bed porosity ε according to

$$u = \frac{\dot{V}_{\text{feed}}}{A_\text{R}\, \varepsilon}\,. \tag{S22}$$

Assuming a bed porosity $\varepsilon = 0.4$ for densely packed fixed beds [3], a particle Reynolds number $\text{Re}_\text{p} \approx 60$ is obtained for a representative GHSV value of $1100\,\text{h}^{-1}$. At very low particle Reynolds numbers ($\text{Re}_\text{p} \approx 1 - 10$), external convective transport may become insufficient to adequately supply reactants to the catalyst surface, resulting in an externally mass-transfer-limited regime [4]. In contrast, the present operating conditions exceed the threshold value of $\text{Re}_\text{p} \approx 50$ reported for heterogeneous gas–solid systems, above which further increases in external convection have only a minor influence on the overall reaction rate.

Consequently, external transport limitations are expected to be negligible. Therefore, the calculated particle Reynolds numbers indicate sufficiently developed convective transport through the packed bed under the investigated operating conditions. For the lowest investigated WHSV values, particle Reynolds numbers as low as $\text{Re}_\text{p} \approx 30$ may occur, slightly below the aforementioned threshold, but still above very low particle Reynolds numbers ($\text{Re}_\text{p} \approx 1 - 10$), thus a certain contribution of external transport limitations cannot be fully excluded under these conditions.

For the present reactor with an inner diameter D_R of 10 mm containing a catalyst sieve fraction with a maximum catalyst particle diameter $d_{\text{p,max}}$ of 2.5 mm and a minimum catalyst particle diameter $d_{\text{p,min}}$ of 1.0 mm, the resultig reactor-to-particle diameter ratio spans the range

$$\frac{D_\text{R}}{d_{\text{p,max}}} = 4 \leq \frac{D_\text{R}}{d_\text{p}} \leq 10 = \frac{D_\text{R}}{d_{\text{p,min}}}\,. \tag{S23}$$

Although the lower bound of this range falls below the value commonly recommended to minimize wall effects [5],

$$\frac{D_\text{R}}{d_\text{p}} \geq 8\,, \tag{S24}$$

the investigated particle size fraction covers reactor-to-particle diameter ratios both below and above this criterion. Consequently, a substantial fraction of the catalyst particles fulfills the recommended range, supporting the assessment that potential wall effects remain acceptable under the investigated conditions.

Furthermore, the simultaneous fulfillment of

$$\frac{L_{\mathrm{bed}}}{d_{\mathrm{p}}} > 50 \qquad \text{and} \qquad \mathrm{Re}_{\mathrm{p}} > 50 \tag{S25}$$

indicates, that the hydrodynamic conditions can be considered sufficient for reliable reactor operation. A further reduction of particle size would improve the reactor-to-particle diameter ratio but would also result in substantially increased pressure drop under the investigated operating conditions. Therefore, the selected particle size fraction represents a compromise between hydrodynamic performance and acceptable pressure drop below 0.3 – 0.5 bar due to pressure sensitivity of the ODHE reactions and overall low reaction pressure regime.

S2 Additional Experimental Data

S2.1 Detailed Experimental Data for Variation of Catalyst Bed Inert Dilution

To demonstrate that the observed trends in product selectivity S_i, product yield Y_i and molar product ratios r_i are independent of the thermal regime, a variation of the catalyst mass M_{cat} was performed by adjusting the catalyst bed inert dilution using Catalst A.

This approach enabled systematic adjustment of the temperature deviation ΔT_{bed} over the catalyst bed and covered a broad range of WHSV values by varying M_{cat}. Low values of WHSV$\leq 1.4\,\mathrm{h}^{-1}$ correspond to process-relevant, non-isothermal conditions, high values of WHSV$\geq 4.4\,\mathrm{h}^{-1}$ correspond to pseudo-isothermal conditions. Values of WHSV in the range of 2.2 – $4.2\,\mathrm{h}^{-1}$ correspond to non-isothermal conditions with lower temperature deviation over the catalyst bed than for process-relevant, non-isothermal conditions, according to Table 2 in Section 2.4 in the main paper. Different marker shapes indicate catalyst bed dilution (temperature deviation over the catalyst bed), while color and filling represent variations in WHSV.

The trends of product selectivites S_i, product yields Y_i and molar product ratios r_i in Figures S3 – S9 are consistent to observed trends in the WHSV variation series in Section 3.4 in the main paper. The correlations between ethane conversion $X_{\mathrm{C_2H_6}}$, product selectivities S_i, product yields Y_i and molar product ratios r_i observed under kinetically oriented, pseudo-isothermal conditions can be confirmed under process-relevant, non-isothermal conditions. Importantly, the commercial product selectivity S_{CP} and commercial product yield Y_{CP} exhibit a systematic trend independent of the applied thermal regime (isothermal vs. non-isothermal), indicating an intrinsic correlation governed primarily by ethane conversion.

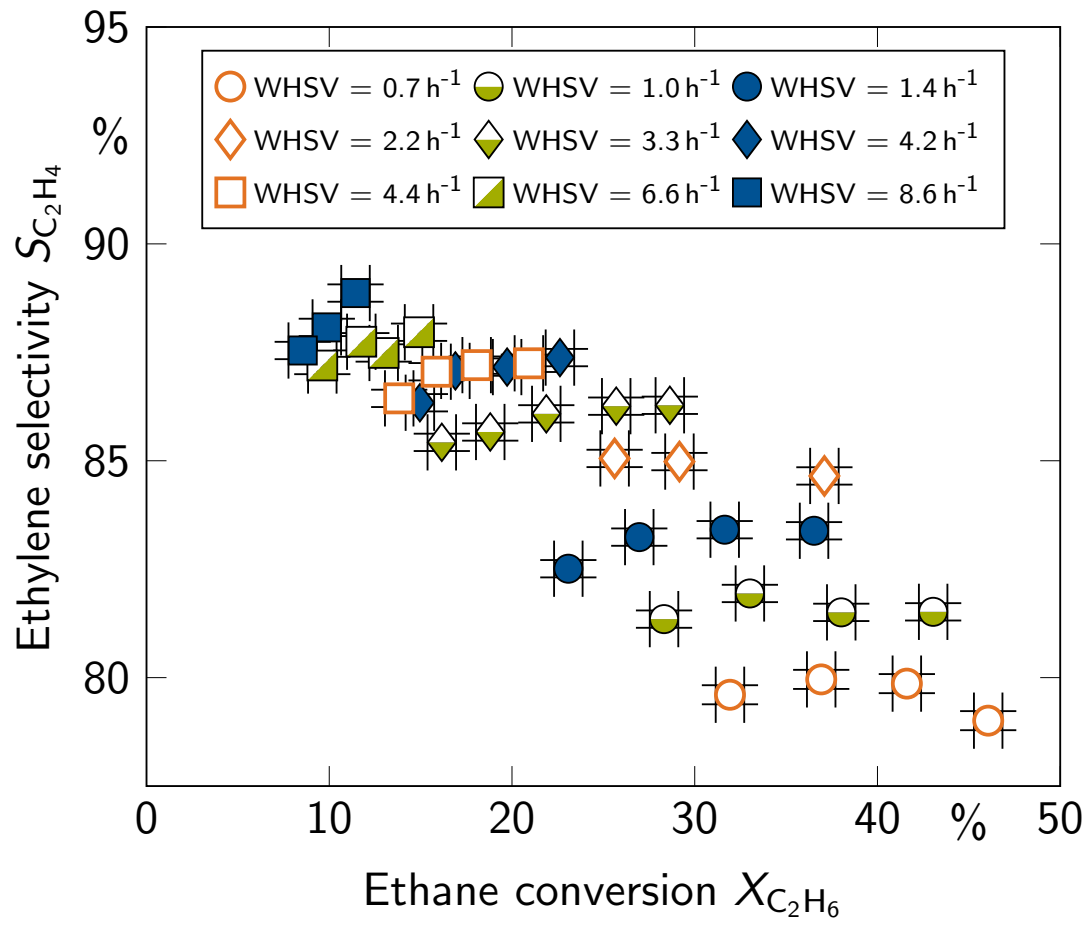

(a)

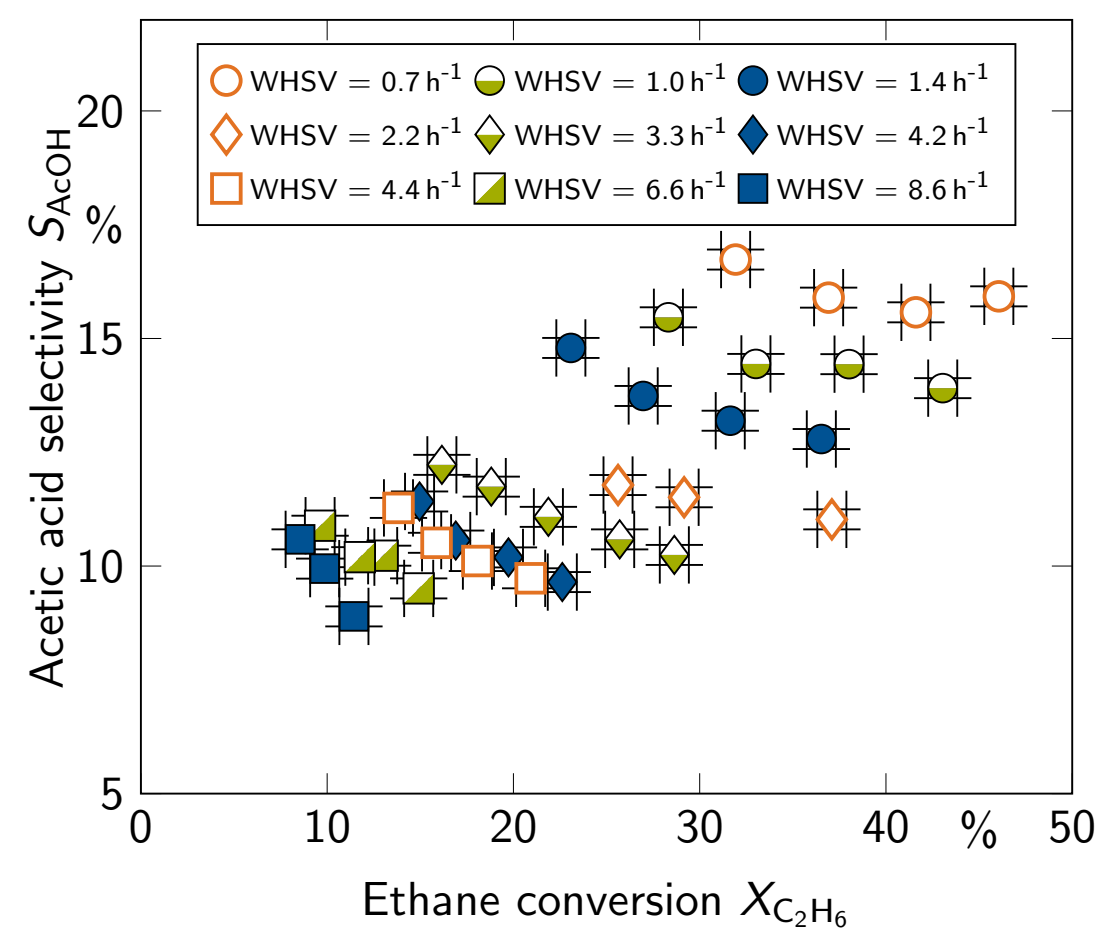

(b)

Figure S3: Dependency of **(a)** $S_{C_2H_4}$ and **(b)** S_{AcOH} on coolant temperature T_{CM} and WHSV at $p_{in} = 3.5$ bar, $r_{O_2/C_2H_6} = 0.373$, $r_{H_2O/C_2H_6} = 0.427$, T_{CM}=300 – 320 °C using Catalyst A. Catalyst bed inert dilution of 0 wt. %, 70 wt. % and 85 wt. % corresponds to WHSV of 0.7 – 1.4 h^{-1}, 2.2 – 4.2 h^{-1} and 4.4 – 8.6 h^{-1} respectively.

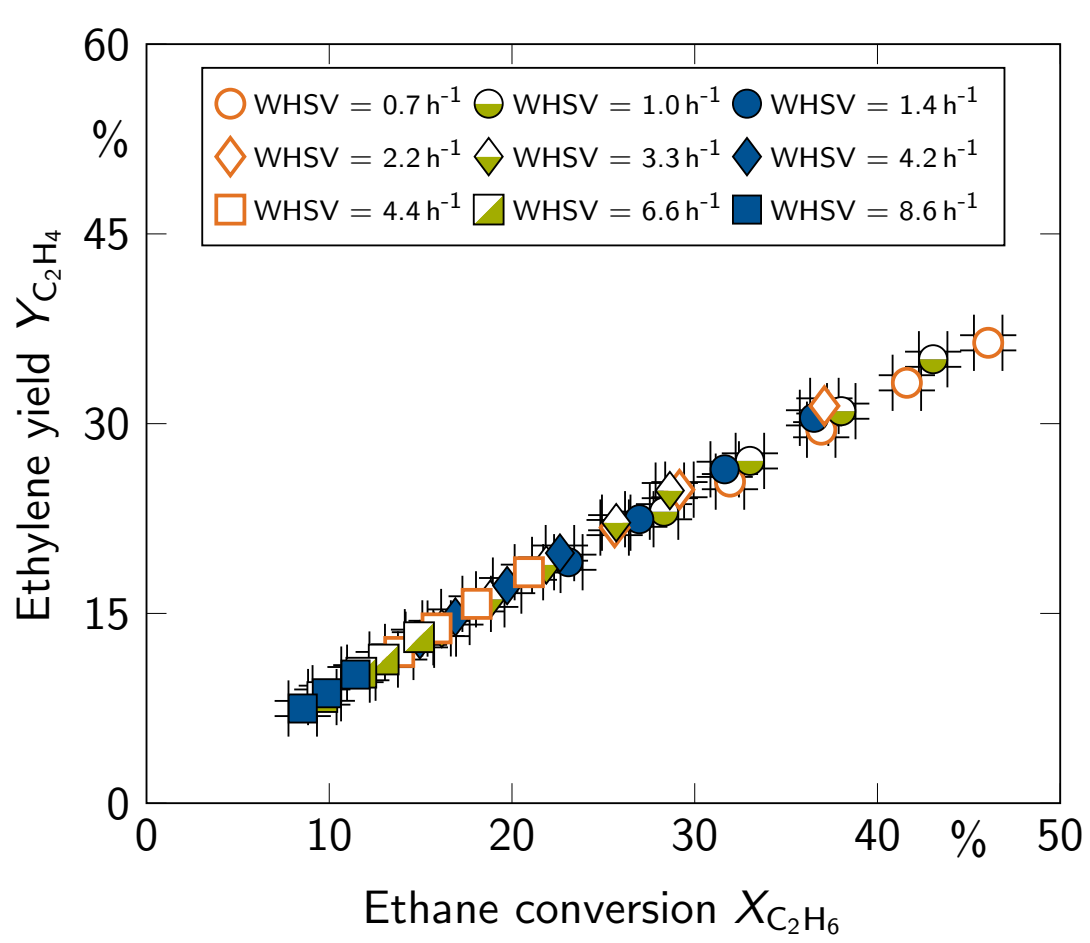

(a)

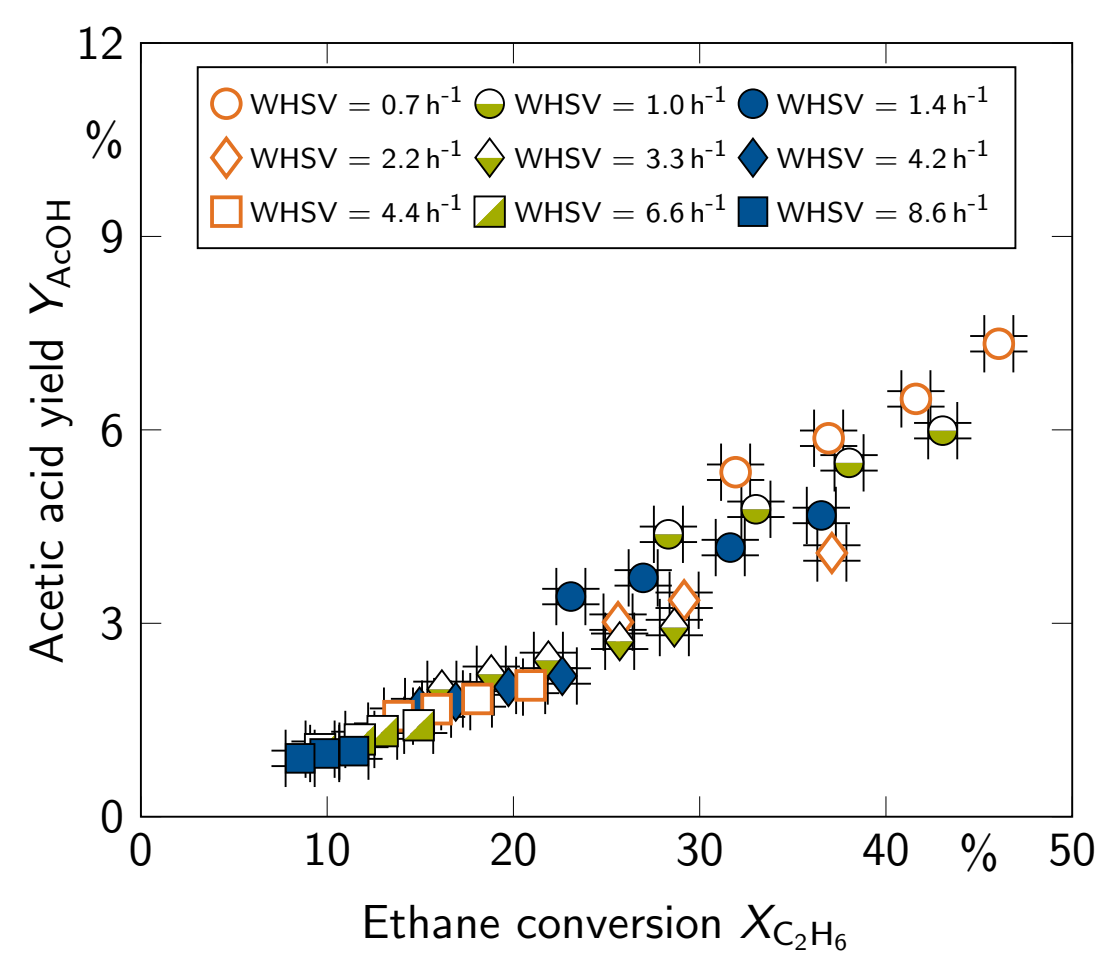

(b)

Figure S4: Dependency of **(a)** $Y_{C_2H_4}$ and **(b)** Y_{AcOH} on coolant temperature T_{CM} and WHSV at $p_{in} = 3.5$ bar, $r_{O_2/C_2H_6} = 0.373$, $r_{H_2O/C_2H_6} = 0.427$, T_{CM}=300 – 320 °C using Catalyst A. Catalyst bed inert dilution of 0 wt. %, 70 wt. % and 85 wt. % corresponds to WHSV of 0.7 – 1.4 h^{-1}, 2.2 – 4.2 h^{-1} and 4.4 – 8.6 h^{-1} respectively.

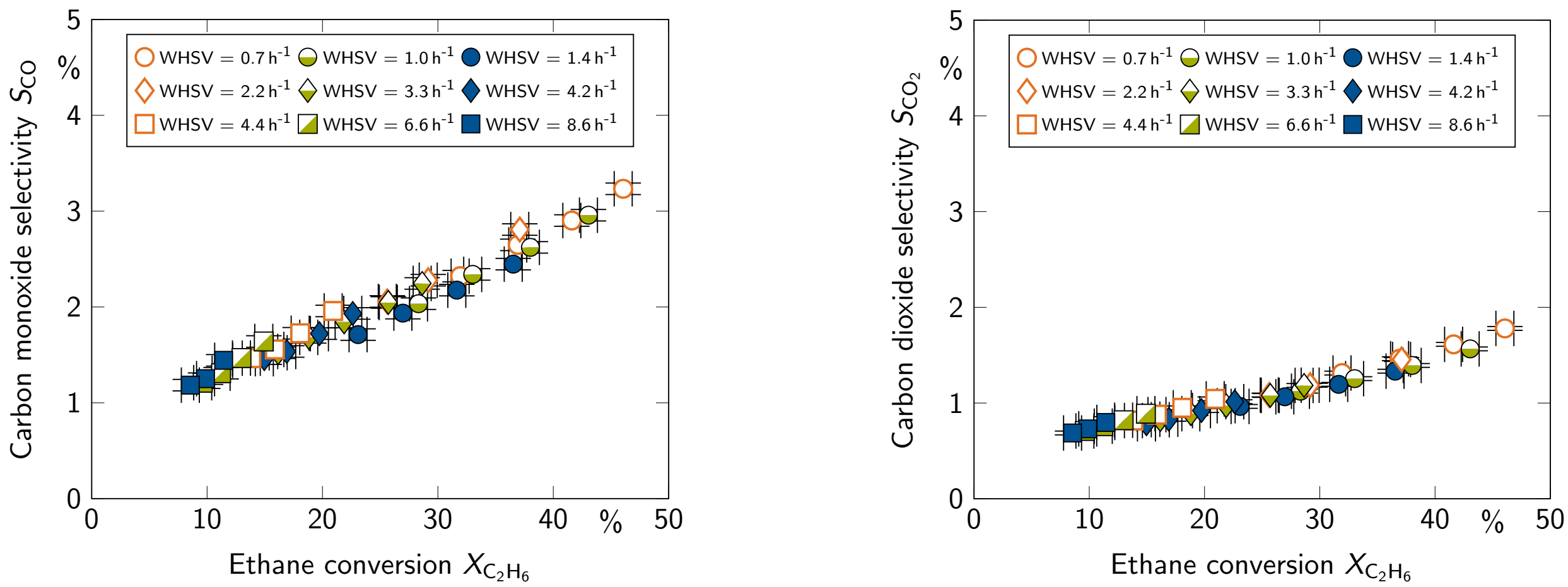

(a) **(b)**

Figure S5: Dependency of **(a)** S_{CO} and **(b)** S_{CO_2} on coolant temperature T_{CM} and WHSV at $p_{in} = 3.5\,bar$, $r_{O_2/C_2H_6} = 0.373$, $r_{H_2O/C_2H_6} = 0.427$, T_{CM}=300 – 320 °C using Catalyst A. Catalyst bed inert dilution of 0 wt. %, 70 wt. % and 85 wt. % corresponds to WHSV of 0.7 – 1.4 h^{-1}, 2.2 – 4.2 h^{-1} and 4.4 – 8.6 h^{-1} respectively.

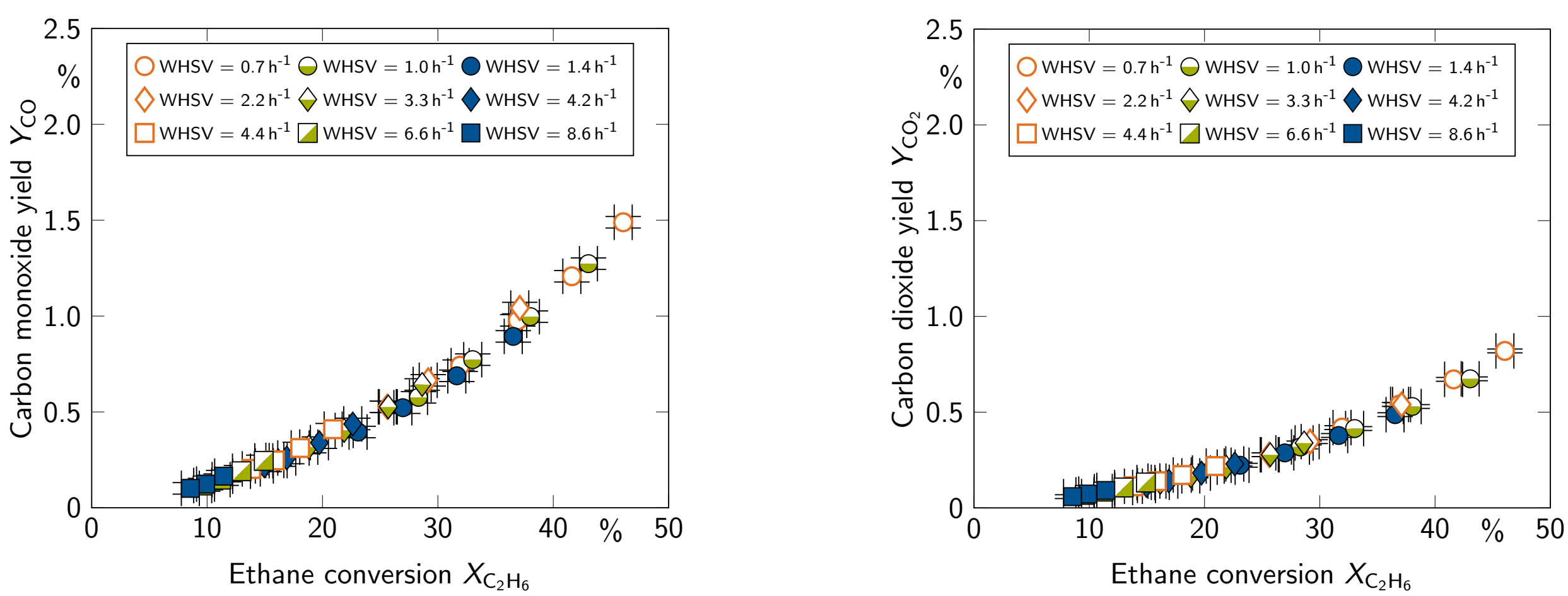

(a) **(b)**

Figure S6: Dependency of **(a)** Y_{CO} and **(b)** Y_{CO_2} on coolant temperature T_{CM} and WHSV at $p_{in} = 3.5\,bar$, $r_{O_2/C_2H_6} = 0.373$, $r_{H_2O/C_2H_6} = 0.427$, T_{CM}=300 – 320 °C using Catalyst A. Catalyst bed inert dilution of 0 wt. %, 70 wt. % and 85 wt. % corresponds to WHSV of 0.7 – 1.4 h^{-1}, 2.2 – 4.2 h^{-1} and 4.4 – 8.6 h^{-1} respectively.

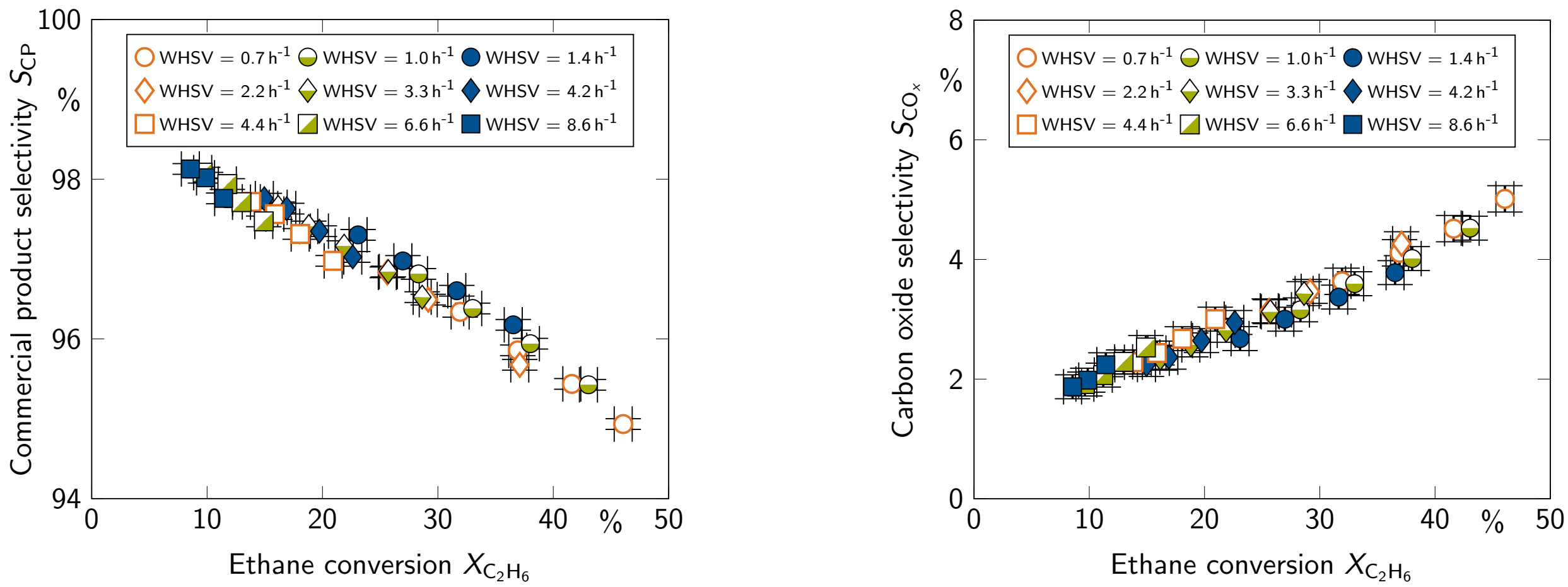

(a) **(b)**

Figure S7: Dependency of **(a)** S_{CP} and **(b)** $S_{\mathrm{CO_x}}$ on coolant temperature T_{CM} and WHSV at $p_{\mathrm{in}} = 3.5\,\mathrm{bar}$, $r_{\mathrm{O_2/C_2H_6}} = 0.373$, $r_{\mathrm{H_2O/C_2H_6}} = 0.427$, T_{CM}=300 – 320 °C using Catalyst A. Catalyst bed inert dilution of 0 wt. %, 70 wt. % and 85 wt. % corresponds to WHSV of 0.7 – 1.4 $\mathrm{h^{-1}}$, 2.2 – 4.2 $\mathrm{h^{-1}}$ and 4.4 – 8.6 $\mathrm{h^{-1}}$ respectively.

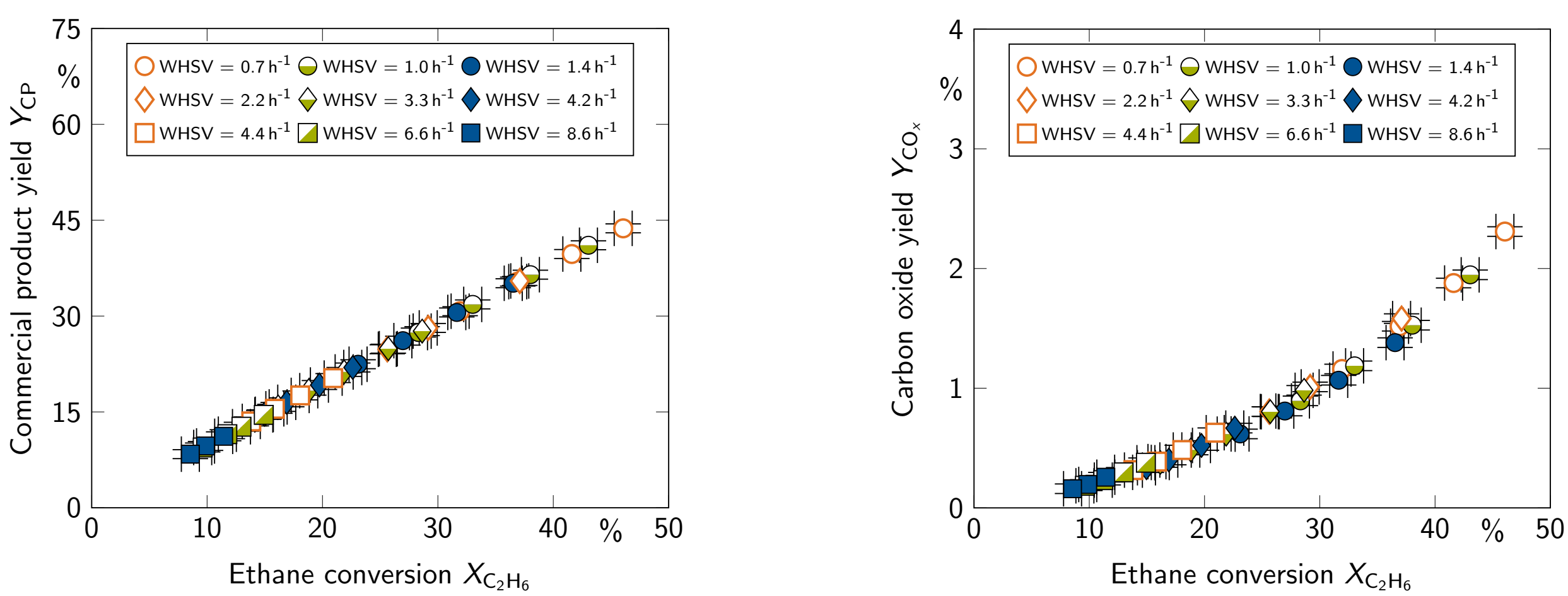

(a) **(b)**

Figure S8: Dependency of **(a)** Y_{CP} and **(b)** $Y_{\mathrm{CO_x}}$ on coolant temperature T_{CM} and WHSV at $p_{\mathrm{in}} = 3.5\,\mathrm{bar}$, $r_{\mathrm{O_2/C_2H_6}} = 0.373$, $r_{\mathrm{H_2O/C_2H_6}} = 0.427$, T_{CM}=300 – 320 °C using Catalyst A. Catalyst bed inert dilution of 0 wt. %, 70 wt. % and 85 wt. % corresponds to WHSV of 0.7 – 1.4 $\mathrm{h^{-1}}$, 2.2 – 4.2 $\mathrm{h^{-1}}$ and 4.4 – 8.6 $\mathrm{h^{-1}}$ respectively.

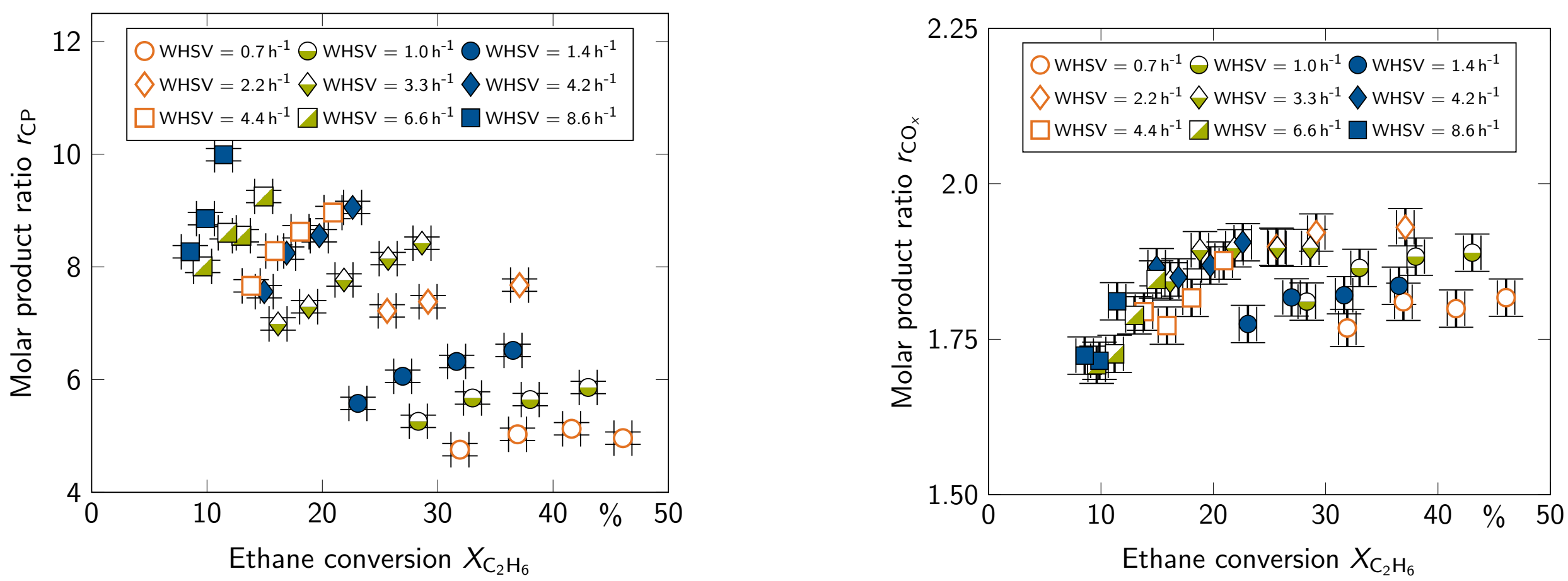

(a) **(b)**

Figure S9: Dependency of **(a)** r_{CP} and **(b)** r_{CO_x} on coolant temperature T_{CM} and WHSV at $p_{in} = 3.5\,\text{bar}$, $r_{O_2/C_2H_6} = 0.373$, $r_{H_2O/C_2H_6} = 0.427$, T_{CM}=300 – 320 °C using Catalyst A. Catalyst bed inert dilution of 0 wt. %, 70 wt. % and 85 wt. % corresponds to WHSV of 0.7 – 1.4 h^{-1}, 2.2 – 4.2 h^{-1} and 4.4 – 8.6 h^{-1} respectively.

S2.2 Discussion of Pressure Influence via Modified Reciprocal GHSV and Steam Partial Pressure

To assess whether the observed changes in product selectivity resulting from variations in reactor inlet pressure p_{in} originate from pressure effects or from the accompanying change in residence time, the sensitivity of the product selectivities to both reactor inlet pressure and WHSV is compared via a modified reciprocal gas hourly space velocity $\mathrm{GHSV}^{-1}_{\mathrm{mod}}$, where the volumetric flow rate employed in its calculation corresponds to the actual pressure-dependent flow rate rather than the flow rate at standard (or normal) conditions.

The modified gas hourly space velocity $\mathrm{GHSV}_{\mathrm{mod}}$ is calcualated as ratio of total volume flow rate $\dot{V}_{\mathrm{feed}}$ of the feed gas and catalyst active mass $M_{\mathrm{cat,act}}$ according to

$$\mathrm{GHSV}_{\mathrm{mod}} = \frac{\dot{V}_{\mathrm{feed}}}{M_{\mathrm{cat,act}}} \,. \tag{S26}$$

The modified reciprocal gas hourly space velocity $\mathrm{GHSV}^{-1}_{\mathrm{mod}}$ is then obtained as the reciprocal of the modified gas hourly space velocity $\mathrm{GHSV}_{\mathrm{mod}}$ according to

$$\mathrm{GHSV}^{-1}_{\mathrm{mod}} = \frac{1}{\mathrm{GHSV}_{\mathrm{mod}}} \,. \tag{S27}$$

In contrast to WHSV, which is defined on a mass basis and therefore remains independent of pressure and temperature, the modified gas hourly space velocity $\mathrm{GHSV}_{\mathrm{mod}}$ is based on the volumetric feed flow rate. Assuming ideal gas behavior, the volumetric flow rate is related to the molar flow rate according to

$$\dot{V}_{\mathrm{feed}} = \frac{\dot{N}_{\mathrm{feed}} R T}{p_{\mathrm{in}}} \,. \tag{S28}$$

For a constant molar feed flow rate $\dot{N}_{\mathrm{feed}}$ and constant temperature T, the volumetric flow rate therefore decreases with increasing reactor inlet pressure p_{in}. Consequently, $\mathrm{GHSV}_{\mathrm{mod}}$ decreases and the modified reciprocal gas hourly space velocity $\mathrm{GHSV}^{-1}_{\mathrm{mod}}$ increases with increasing pressure. This allows the influence of pressure-induced changes in gas-phase residence time to be assessed separately from the effects directly related to reaction pressure.

The sensitivity of ethane conversion $X_{\mathrm{C_2H_6}}$ and product selectivity S_i with modified reciprocal gas hourly space velocity $\mathrm{GHSV}^{-1}_{\mathrm{mod}}$ are compared for two experiment series (variation of Inert dil., Section S2.1 (SI), and variation of p_{in}). Regarding the experiment series of catalyst bed inert dilution variation, only experiments conducted at equal reaction conditions as for variation of reactor inlet pressure, specifically an inert dilution of 85 wt. % of the catalyst bed and a coolant temperature T_{CM} of 315 °C. Figure S10 shows the influence of varied reactor inlet pressure p_{in} and WHSV on ethane conversion $X_{\mathrm{C_2H_6}}$ and product selectivity S_i compared via modified reciprocal gas hourly space velocity $\mathrm{GHSV}^{-1}_{\mathrm{mod}}$.

The comparison shows that variations in reactor inlet pressure and WHSV exhibit an identical influence on ethane conversion, as both parameters affect ethane conversion solely through their impact on the modified reciprocal gas hourly space velocity $\mathrm{GHSV}^{-1}_{\mathrm{mod}}$. However, the reactor inlet pressure shows a significantly stronger effect on product selectivities, which can be attributed to a significant increase in steam partial pressure $p_{\mathrm{H_2O,effluent}}$ in the wet product gas with higher reactor inlet pressure p_{in}. In contrast, during WHSV variation the steam partial pressure changes only marginally due to varied ethane conversion.

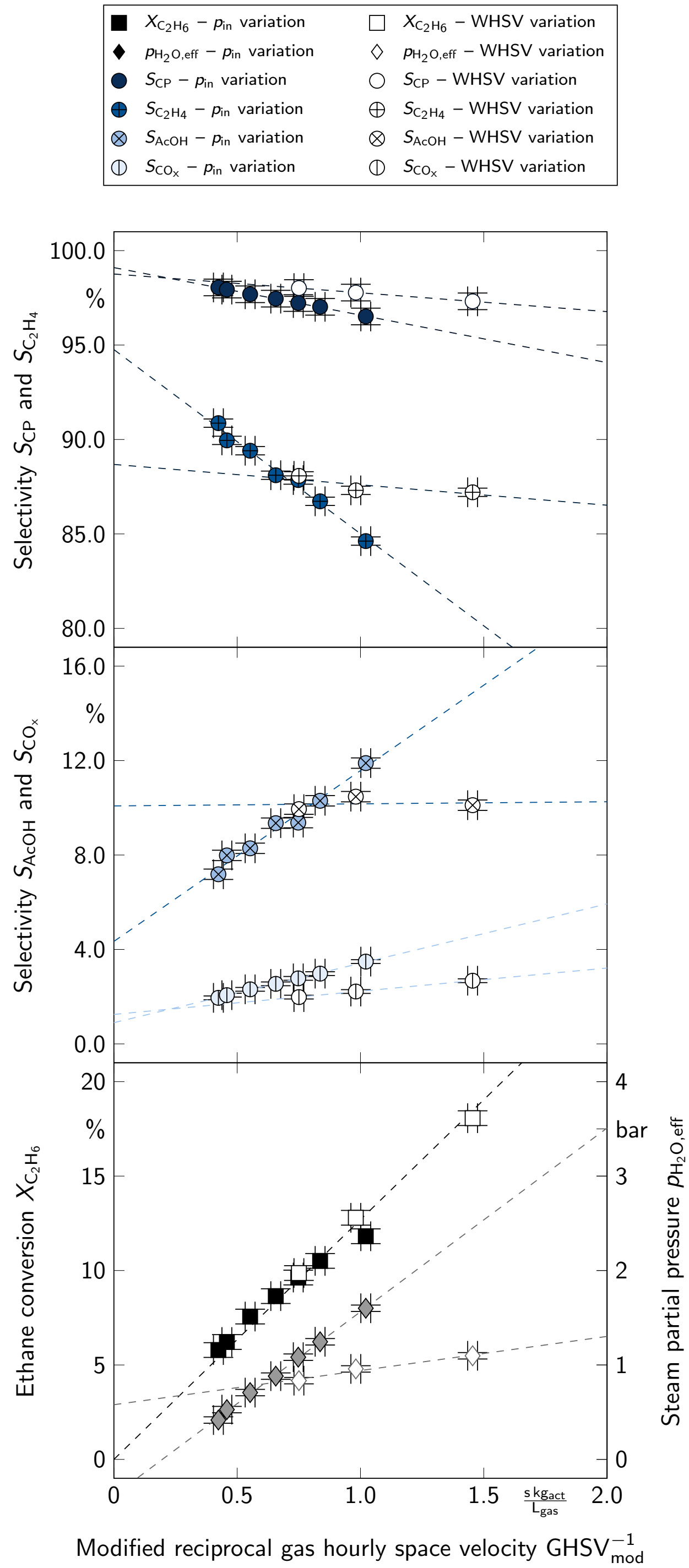

Figure S10: Dependency of reactor performance parameters on reactor inlet pressure and WHSV compared via modified reciprocal gas hourly space velocity $GHSV_{mod}^{-1}$.

Figure S11 shows the apparent reaction rate constant k as a function of the steam partial pressure $p_{H_2O,feed}$ for both the variation of reactor inlet pressure p_{in} and the variation of the steam-to-ethane ratio r_{H_2O/C_2H_6}. For both parameter variations, a characteristic change in the behavior of the apparent reaction rate constant k is observed at a steam partial pressure of approximately $p_{H_2O,feed} \approx 0.75$ bar.

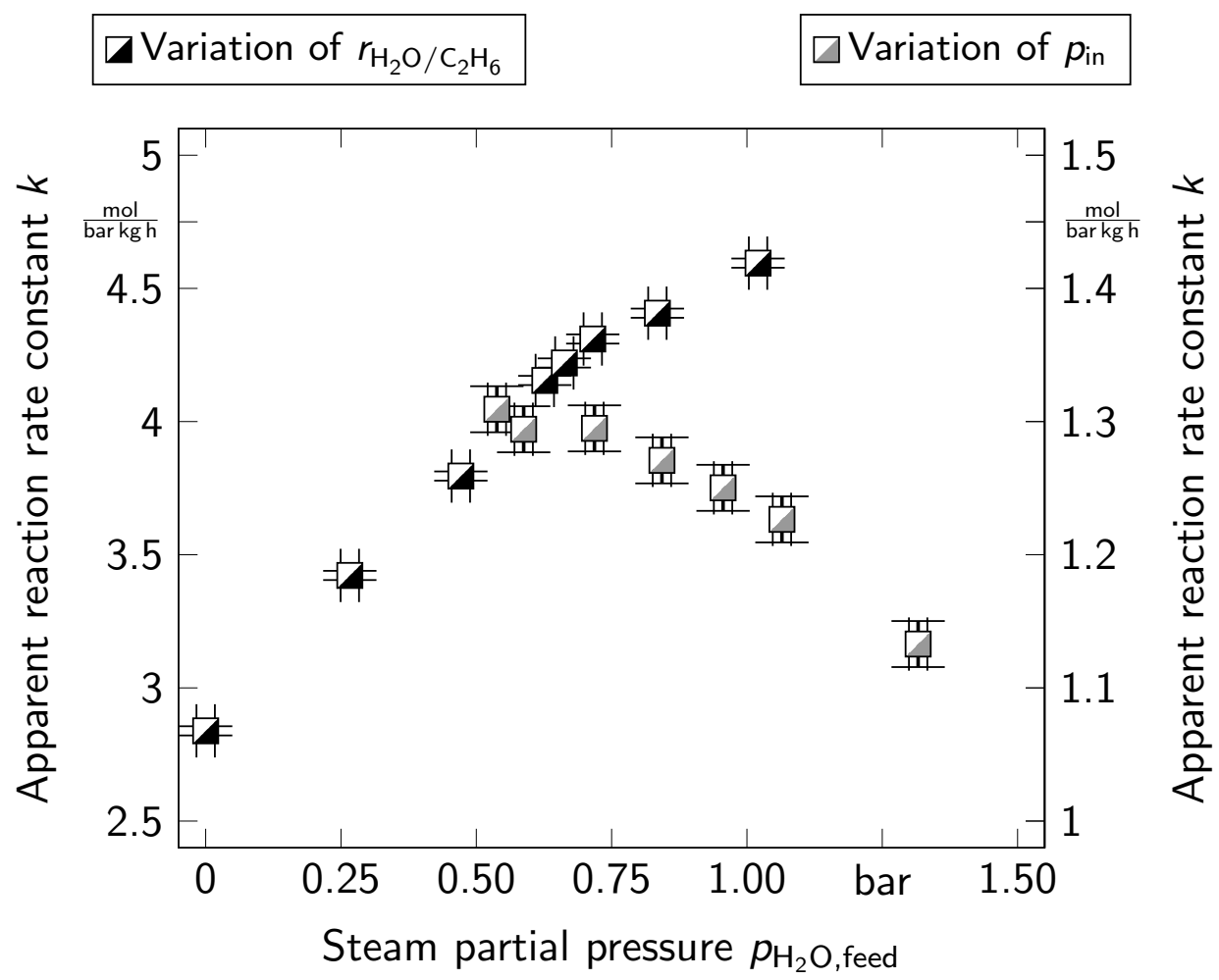

Figure S11: Dependency of apparent reaction rate constant k on steam partial pressure $p_{H_2O,feed}$ for variation of reactor inlet pressure and molar steam-to-ethane ratio.

For the variation of the steam-to-ethane ratio r_{H_2O/C_2H_6}, the apparent reaction rate constant k continues to increase beyond this value, but with reduced slope. In contrast, during variation of the reactor inlet pressure p_{in}, the apparent reaction rate constant k is constant for low reactor inlet pressure, but decreases significantly once the steam partial pressure in the reactor feed exceeds $p_{H_2O,feed} \approx 0.75$ bar. This corresponds to a reactor inlet pressure of $p_{in} \approx 3$ bar. Both data sets therefore indicate that elevated steam partial pressures increasingly affect the apparent catalyst activity.

This behavior is consistent with literature reports describing inhibitory effects of water on related Mo-V-Nb-based mixed oxide catalysts, including $MoVNbPdO_x$ and $MoVNbO_x$ materials, which have been attributed to increased surface coverage by adsorbed water species [6, 7]. Although the catalyst investigated in the present work additionally contains tellurium and no palladium, it is based on the same M1-type Mo-V-Nb oxide framework that is generally considered responsible for catalytic performance in these catalyst systems. Therefore, a qualitatively similar interaction between steam and active surface sites appears plausible. Although the apparent reaction rate constant k remains an overall activity measure derived from simplified kinetic assumptions, the observed change around $p_{H_2O,feed} \approx 0.75$ bar suggests that steam partial pressure becomes a relevant factor influencing catalyst performance under the investigated conditions.

S2.3 Primary and Secondary Product Discussion

Isothermal experiments with Catalyst A are analyzed to determine whether the observed main and side products are of apparent primary or secondary nature. Selectivities of individual products S_i are analyzed as function of space time WHSV^{-1} and extrapolated to ethane conversion of $X_{C_2H_6} \approx 0$ % (see Figures S12 and S13). All products exhibit a positive y-intercept (marked with circles), indicating a potential contribution from apparent primary formation pathways.

Ethylene selectivity $S_{C_2H_4}$ shows a decreasing linear trend with increasing space time, confirming that ethylene is the only true primary product. In contrast, acetic acid, CO and CO_2 show increasing trends with increasing space time, indicating contributions from secondary formation pathways.

However, it should be noted that the extrapolated selectivities for carbon oxides at vanishing ethane conversion remain very small, suggesting that their formation is still predominantly governed by consecutive oxidation reactions.

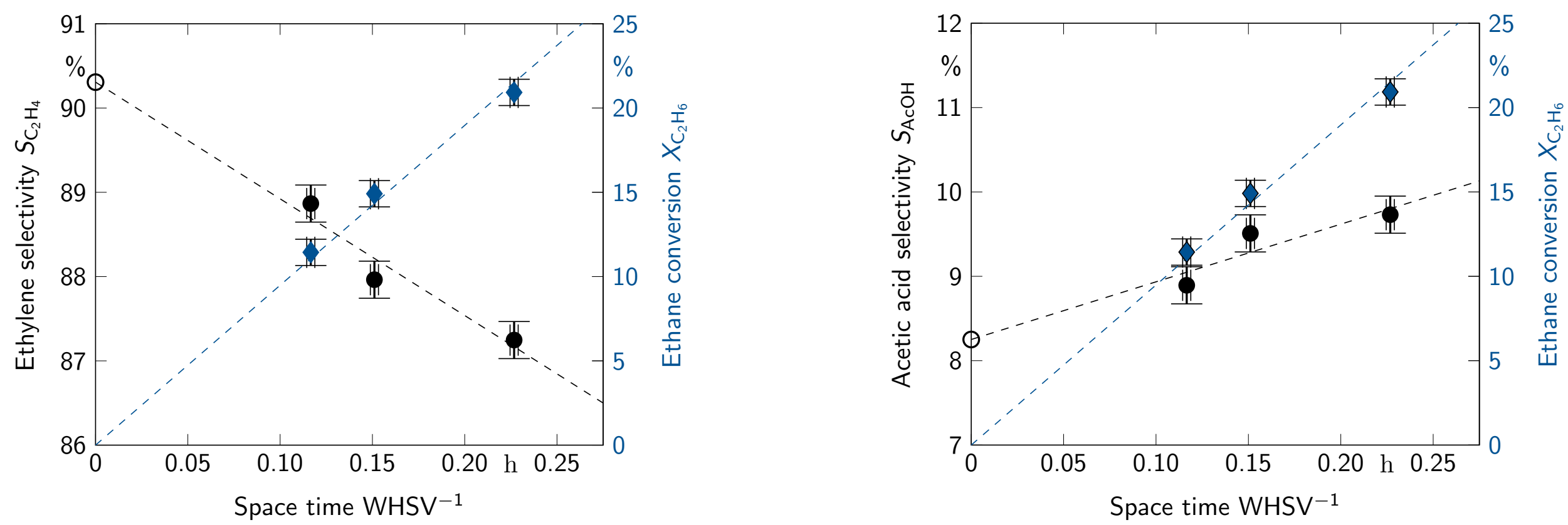

(a) (b)

Figure S12: Dependency of **(a)** $S_{C_2H_4}$ and **(b)** S_{AcOH} on space time WHSV^{-1} under pseudo-isothermal conditions at $p_{in} = 3.5$ bar, $r_{O_2/C_2H_6} = 0.373$, $r_{H_2O/C_2H_6} = 0.427$, WHSV = 4.4 – 8.6 h^{-1}, $T_{CM} = 320$ °C with 85 wt. % catalyst bed inert dilution using Catalyst A.

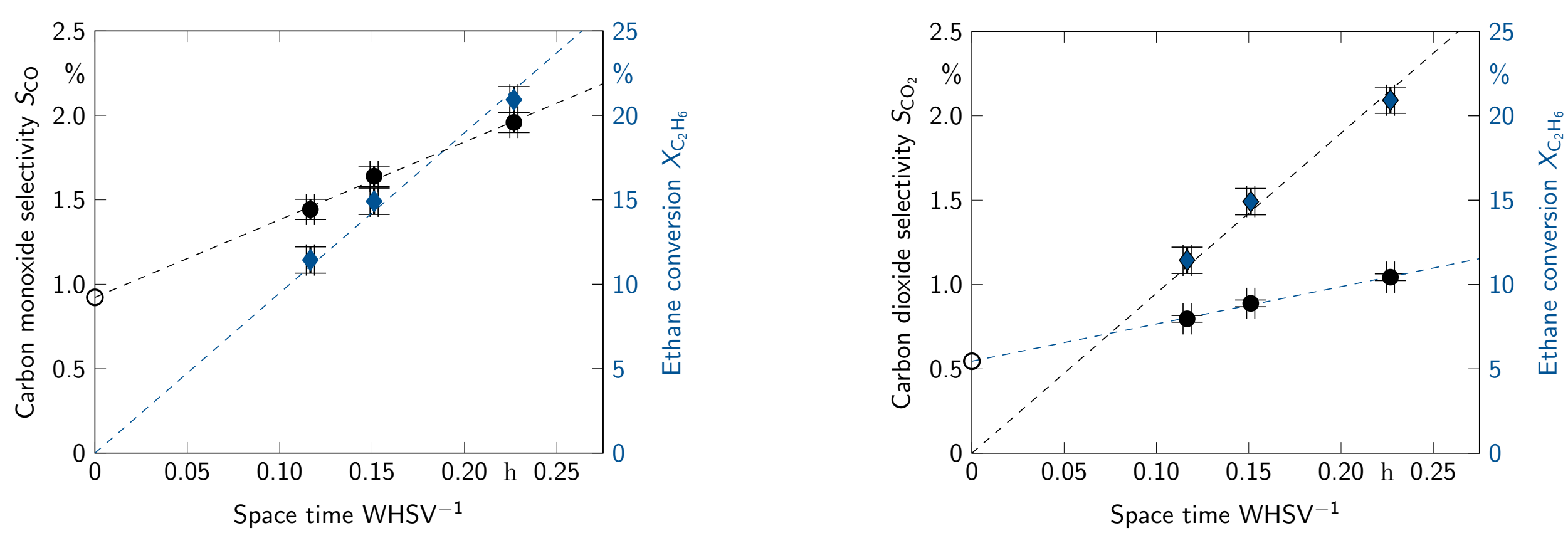

(a) (b)

Figure S13: Dependency of **(a)** S_{CO} and **(b)** S_{CO_2} on space time WHSV^{-1} under pseudo-isothermal conditions at $p_{in} = 3.5$ bar, $r_{O_2/C_2H_6} = 0.373$, $r_{H_2O/C_2H_6} = 0.427$, WHSV = 4.4 – 8.6 h^{-1}, $T_{CM} = 320$ °C with 85 wt. % catalyst bed inert dilution using Catalyst A.

S2.4 Additional Experimental Data for Variation of Coolant Temperature and WHSV

The dependency of molar ratio r_{CO_x} of carbon oxides and total carbon oxide selectivity S_{CO_x} on coolant temperature T_{CM} and WHSV is shown in Figure S14.

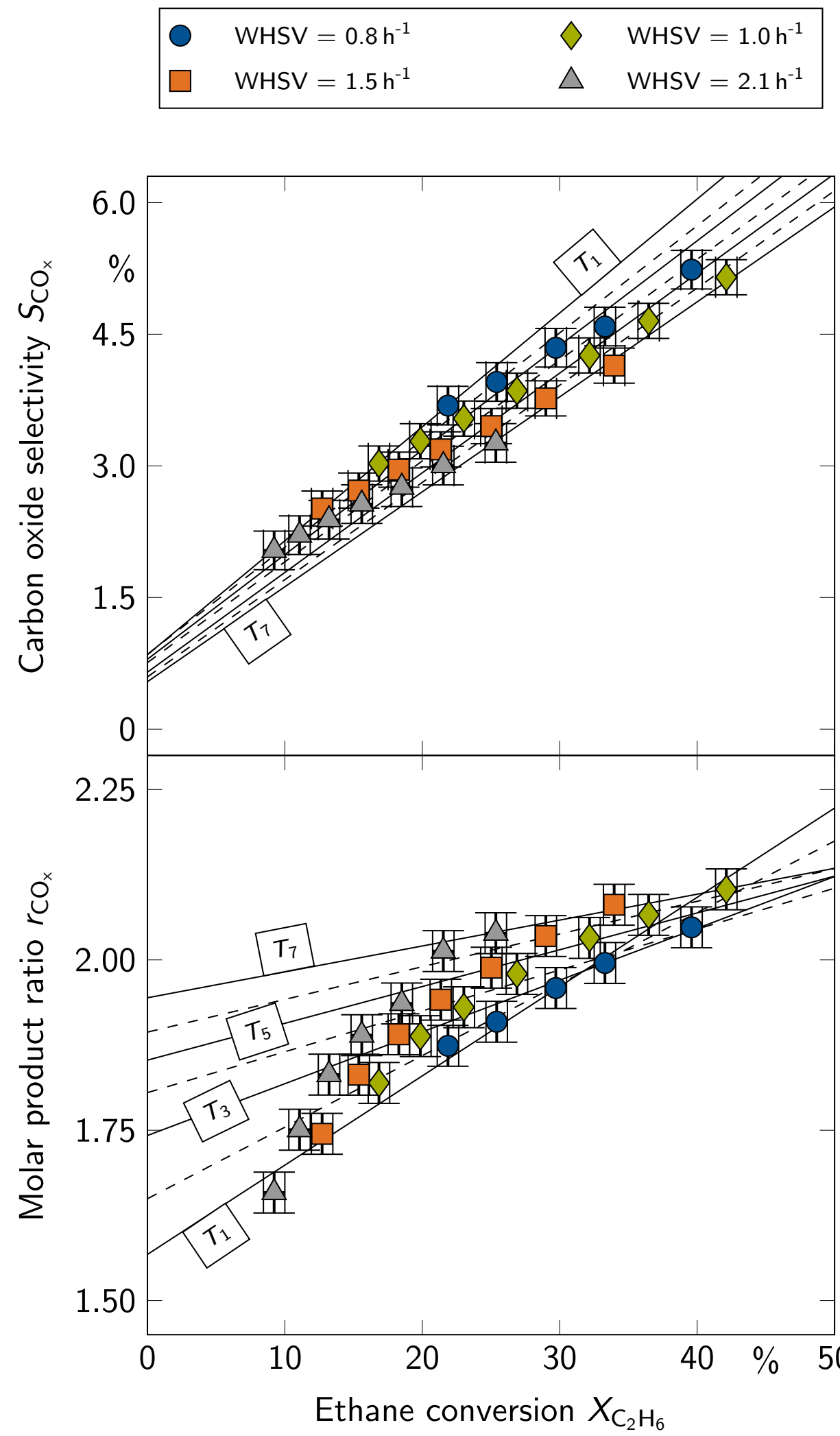

Figure S14: Dependency of S_{CO_x} and r_{CO_x} on coolant temperature T_{CM} and WHSV at $p_{in} = 3.5\,\mathrm{bar}$, $r_{O_2/C_2H_6} = 0.373$, $r_{H_2O/C_2H_6} = 0.427$, $T_{CM} = 275 - 305\,^\circ\mathrm{C}$ with non-diluted catalyst bed using Catalyst B.

The carbon oxide selectivity S_{CO_x} and molar ratio r_{CO_x} increase with coolant temperature T_{CM} and space time, reflecting the growing impact of total oxidation at higher ethane conversion $X_{C_2H_6}$. This trend corresponds to the decline in commercial product selectivity S_{CP}. Higher WHSV at constant coolant temperature T_{CM} leads to decreasing ethane conversion $X_{C_2H_6}$ and linearly decreasing S_{CO_x} (linear isotherms).

The molar product ratio r_{CO_x} decreases with increasing WHSV at constant coolant temperature T_{CM}, with diminishing significance at elevated temperatures (linear isotherms). For high ethane conversion ($X_{C_2H_6} \geq 40\,\%$), the molar product ratio r_{CO_x} approaches a constant value of $r_{CO_x} \approx 2.1$. These observations underline that controlling WHSV and average catalyst bed temperature T_{bed} is essential to minimize carbon oxide formation while maintaining process efficiency.

In addition to the product selectivities S_i discussed in the main manuscript, the corresponding product yields Y_i are presented in Figures S15 – S19. The yield-based representation complements the selectivity

analysis by accounting for simultaneous changes in ethane conversion $X_{C_2H_6}$, thereby providing a direct measure of product formation under the investigated operating conditions.

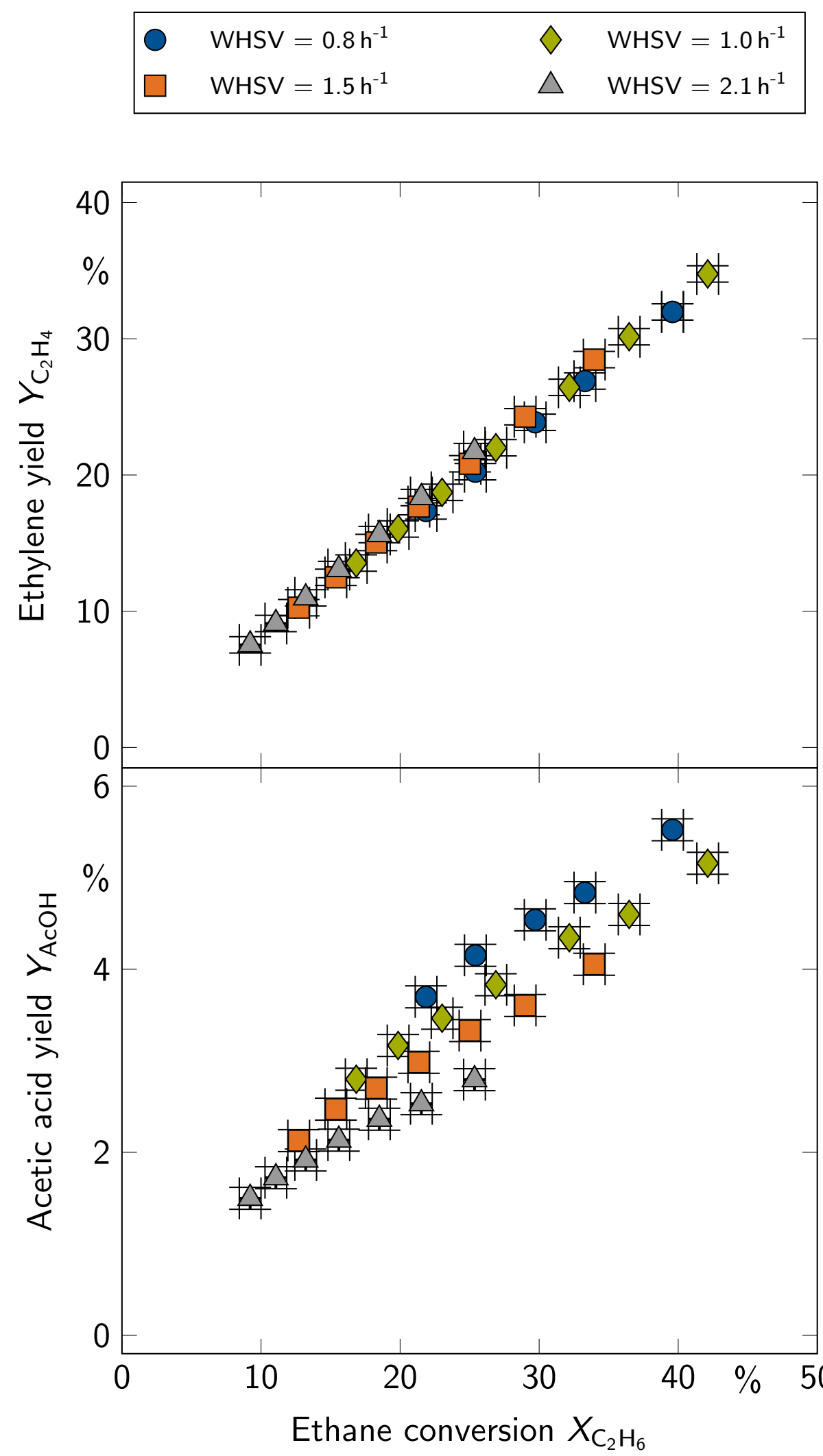

Figure S15: Dependency of $Y_{C_2H_4}$ (top) and Y_{AcOH} (bottom) on coolant temperature T_{CM} and WHSV.

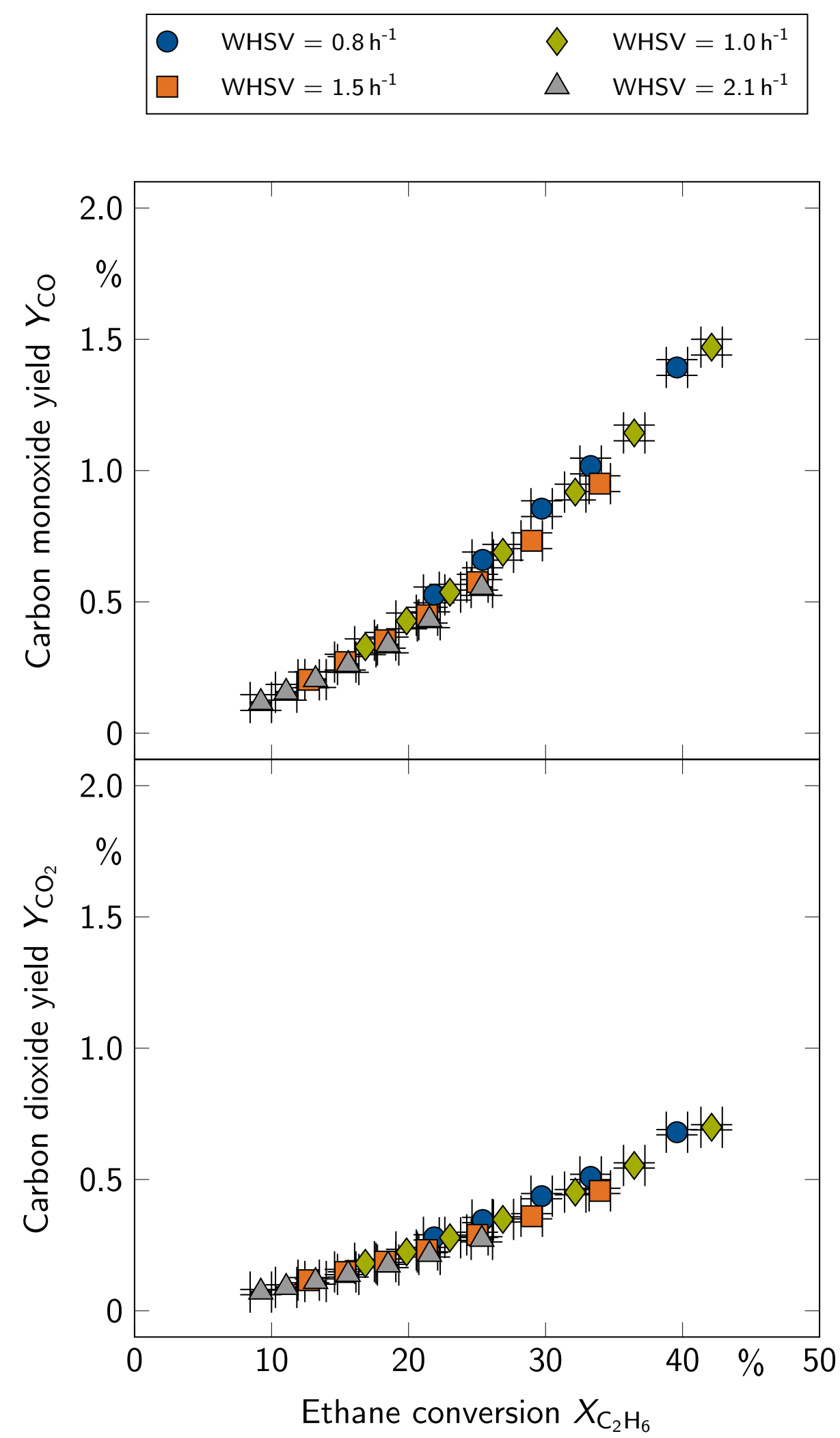

Figure S16: Dependency of Y_{CO} (top) and Y_{CO_2} (bottom) on coolant temperature T_{CM} and WHSV.

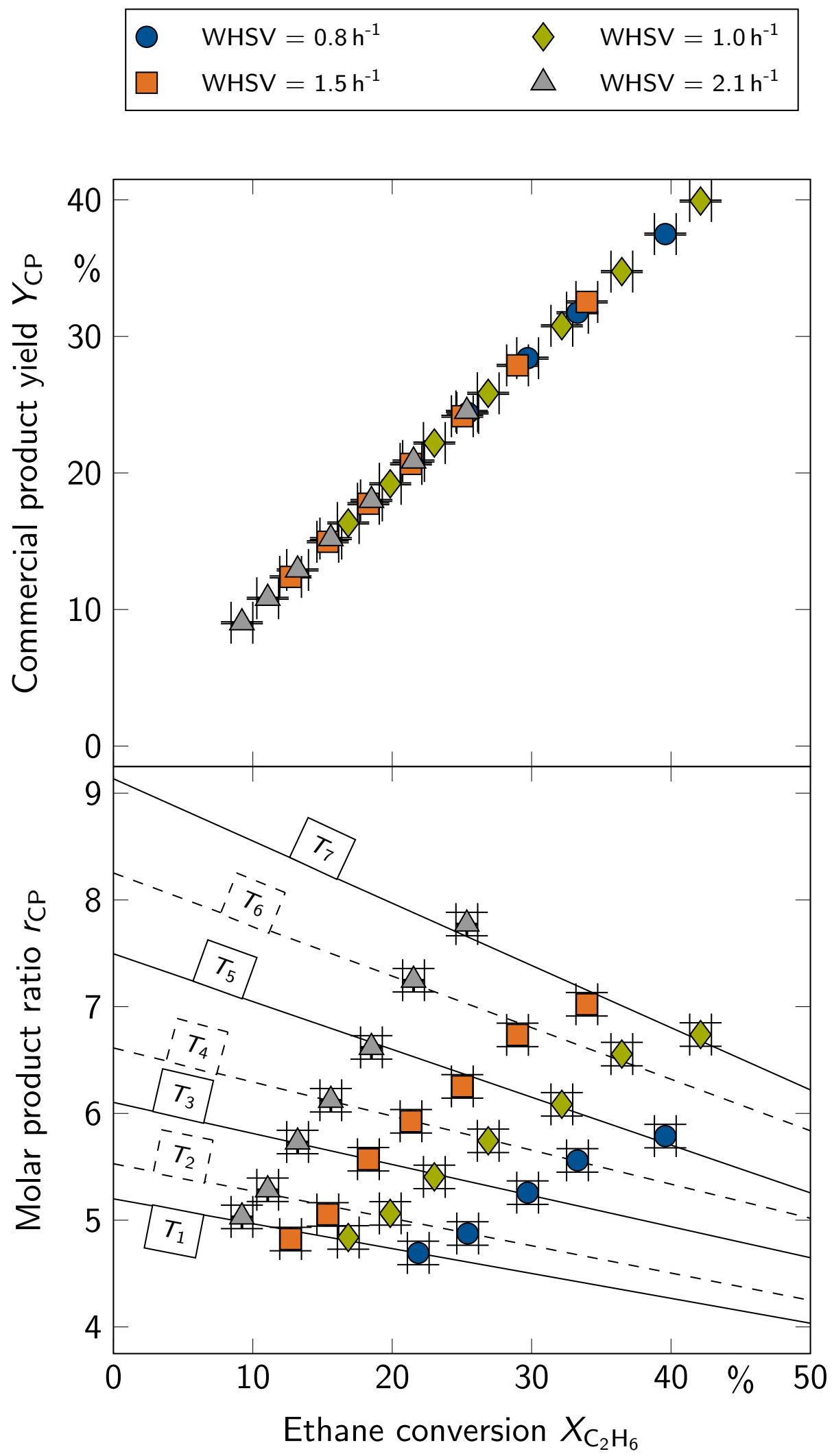

Figure S17: Dependency of Y_{CP} (top) and r_{CP} (bottom) on coolant temperature T_{CM} and WHSV.

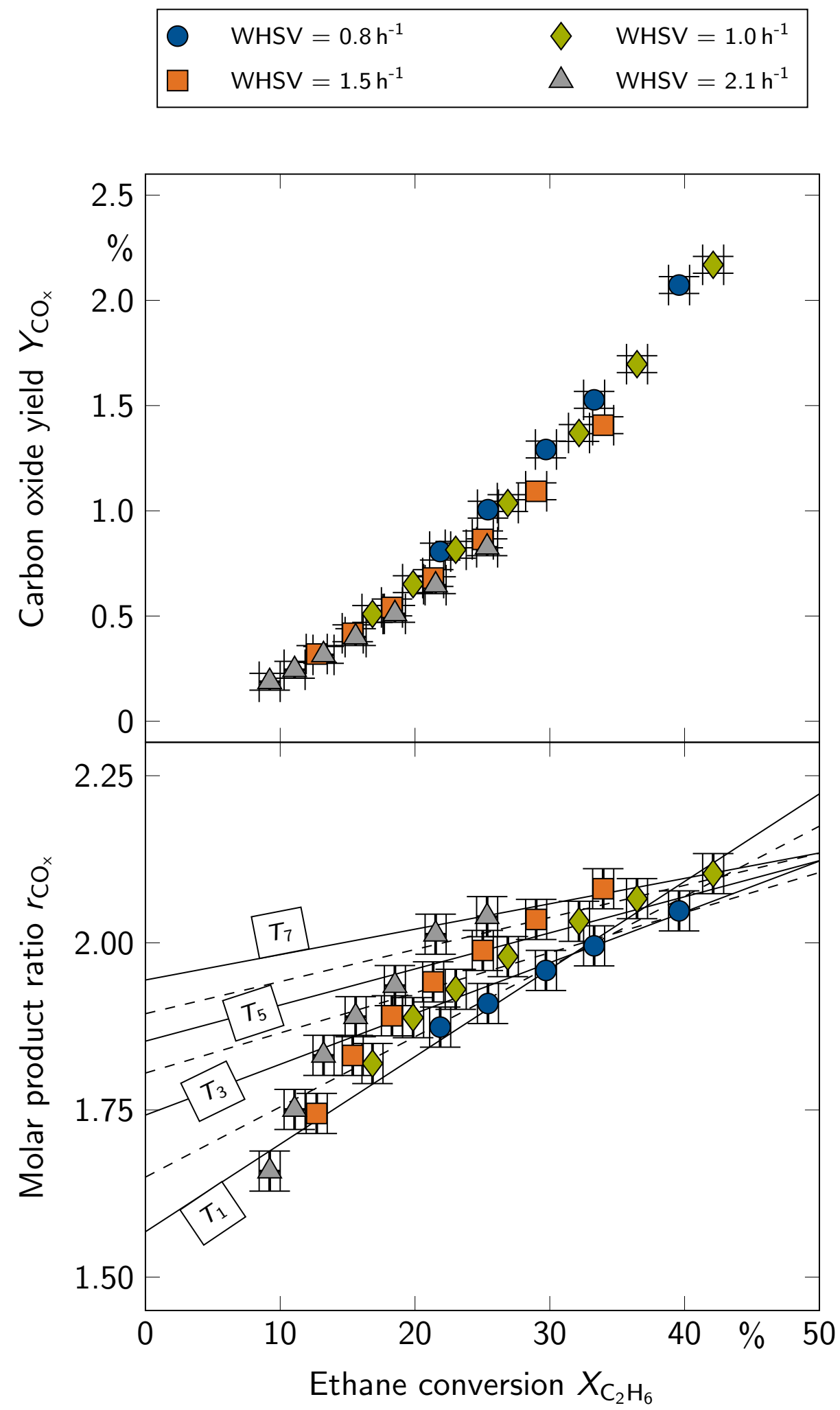

Figure S18: Dependency of $Y_{\mathrm{CO_x}}$ and $r_{\mathrm{CO_x}}$ on coolant temperature T_{CM} and WHSV.

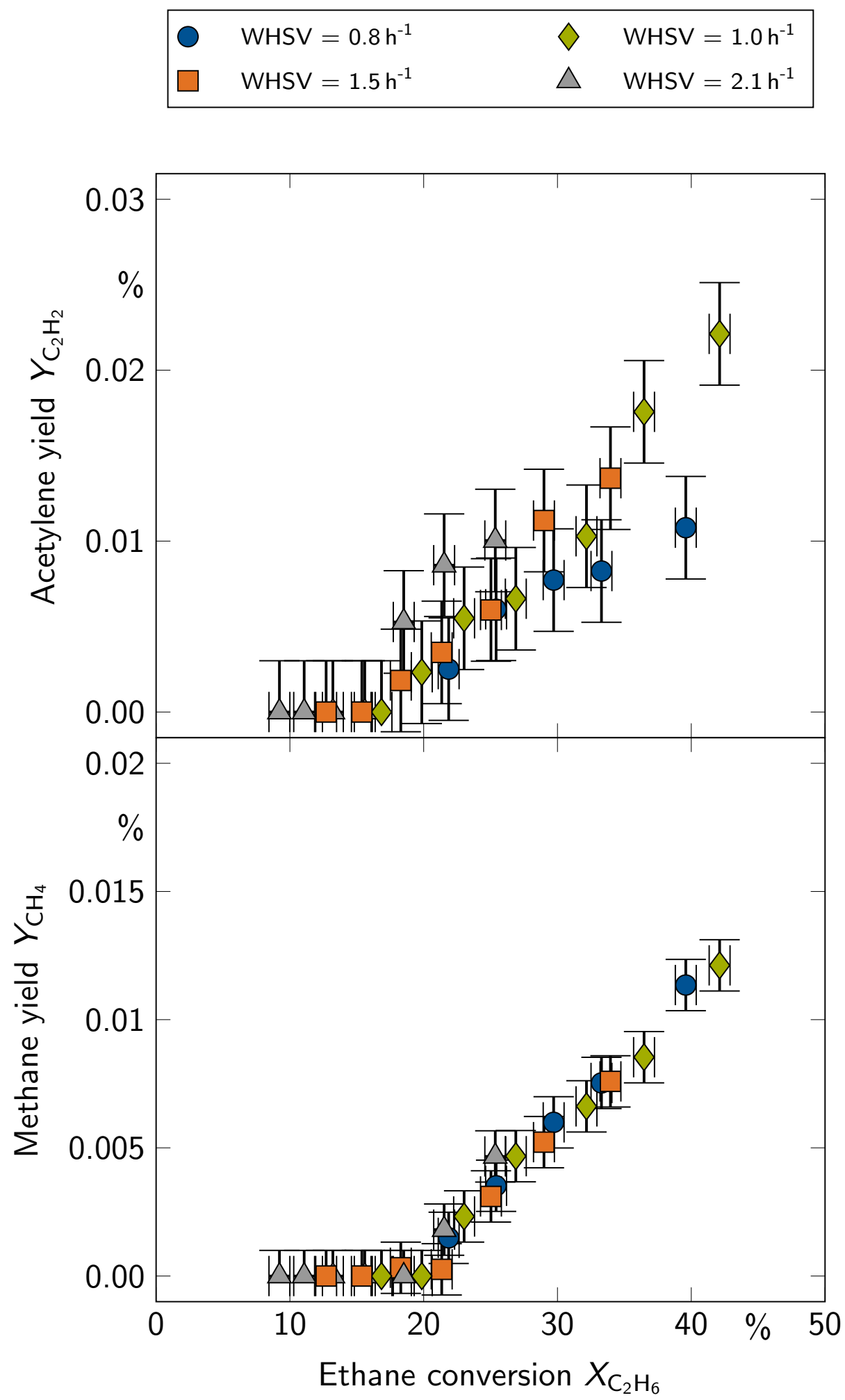

Figure S19: Dependency of Y_{CH_4} (top) and $Y_{C_2H_2}$ (bottom) on coolant temperature T_{CM} and WHSV.

Nomenclature

ΔT_{bed}	Temperature deviation over catalyst bed [K]
ΔT_{max}	Maximum temperature increase [K]
ε	Bed porosity [-]
μ_i	Dynamic viscosity of component i [Pa s]
μ_{mix}	Dynamic viscosity of gas mixture [Pa s]
ν	Stoichiometric coefficient [-]
$\nu_{\mathrm{c},i}$	Number of carbon atoms in component i [-]
ρ_i	Density of component i [kg m^{-3}]
ρ_{mix}	Density of gas mixture [kg m^{-3}]
σ	Standard deviation [-]
A_i	Apparent pre-exponential factor of product formation []
A_{R}	Reactor cross-sectional area [m^2]
d_{p}	Catalyst particle diameter [mm]
$d_{\mathrm{p,max}}$	Maximum catalyst particle diameter in sieve fraction [mm]
$d_{\mathrm{p,min}}$	Minimum catalyst particle diameter in sieve fraction [mm]
D_{R}	Reactor inner diameter [mm]
$E_{\mathrm{A,app},i}$	Apparent activation energy of formation of product i [kJ mol^{-1}]
$\mathrm{GHSV}_{\mathrm{mod}}$	Modified gas hourly space velocity [$\mathrm{L_{gas}\,s^{-1}\,kg_{act}^{-1}}$]
k	Apparent reaction rate constant [mol bar^{-1} kg^{-1} h^{-1}]
k'	Apparent activity parameter [mol kg^{-1} h^{-1}]
k_i	Product-specific apparent reaction rate constant [mol bar^{-1} kg^{-1} h^{-1}]
k'_i	Product-specific apparent activity parameter [mol kg^{-1} h^{-1}]
L_{bed}	Catalyst bed length [cm]
M_{cat}	Catalyst mass [kg]
$M_{\mathrm{cat,act}}$	Active catalyst mass [kg]
M_{cond}	Condensate mass [g]
$\dot{M}_{\mathrm{DPG}}$	Mass flow rate of dry product gas [g h^{-1}]
$\dot{N}_i$	Molar flow rate of component i [mol h^{-1}]
$\dot{N}_{\mathrm{feed}}$	Total feed molar flow rate [mol h^{-1}]
$\dot{N}_{\mathrm{C_2H_6,conv}}$	Molar flow rate of converted ethane [mol h^{-1}]
p_i	Partial pressure of component i [bar]
p_{in}	Reactor inlet pressure [bar]
p_{out}	Reactor outlet pressure [bar]
R	Universal gas constant [J mol^{-1} K^{-1}]
r	Reaction rate [mol kg^{-1} h^{-1}]
$r_{i/j}$	Molar ratio of components i and j [-]
Re_{p}	Particle Reynolds number [-]
S_i	Selectivity of product i [-]
T_{bed}	Average catalyst bed temperature [K]
T_{CM}	Coolant temperature [°C]
u	Superficial gas velocity [m s^{-1}]
$\dot{V}_{\mathrm{feed}}$	Volumetric feed flow rate [$\mathrm{L_{gas}\,s^{-1}}$]
WHSV	Weight hourly space velocity [$\mathrm{g_{feed}\,g_{cat}^{-1}\,h^{-1}}$]
x	Reaction order with respect to ethane [-]
x_i	Molar fraction of component i [-]
X_i	Conversion of reactant i [-]
y	Reaction order with respect to oxygen [-]
Y_i	Yield of product i [-]
Z_1	Carbon balance term for ethane oxidation [-]
Z_2	Carbon balance term for ethylene oxidation [-]
φ_i	Volume fraction of component i [-]

References

(1) Lemmon, E. W.; Bell, I. H.; Huber, M. L.; McLinden, M. O., Thermophysical Properties of Fluid Systems, National Institute of Standards and Technology. `https://webbook.nist.gov/chemistry/fluid/` (accessed 07/15/2026).

(2) Von Seckendorff, J.; Hinrichsen, O. Review on the Structure of Random Packed-Beds. *Can. J. Chem. Eng.* **2021**, *99*, 161–189. DOI: `10.1002/cjce.23959`.

(3) Wu, C. C.; Hwang, G. J. Flow and Heat Transfer Characteristics Inside Packed and Fluidized Beds. *J. Heat Transf.* **Aug. 1998**, *120*, 667–673. DOI: `10.1115/1.2824335`.

(4) Huo, G.; Guo, X. Numerical Analyses of Heterogeneous CLC Reaction and Transport Processes in Large Oxygen Carrier Particles. *Processes* **2021**, *9*, 125. DOI: `10.3390/pr9010125`.

(5) Chu, C. F.; Ng, K. M. Flow in Packed Tubes with a Small Tube to Particle Diameter Ratio. *AIChE Journal* **1989**, *35*, 148–158. DOI: `10.1002/aic.690350116`.

(6) Linke, D.; Wolf, D.; Baerns, M.; Zeyß, S.; Dingerdissen, U. Catalytic Partial Oxidation of Ethane to Acetic Acid over Mo1V0.25Nb0.12Pd0.0005Ox: II. Kinetic Modelling. *J. Catal.* **2002**, *205*, 32–43. DOI: `10.1006/jcat.2001.3368`.

(7) Li, X.; Iglesia, E. Kinetics and Mechanism of Ethane Oxidation to Acetic Acid on Catalysts Based on Mo−V−Nb Oxides. *J. Phys. Chem. C.* **2008**, *112*, 15001–15008. DOI: `10.1021/jp801488y`.
